# Genome-wide association mapping identifies novel SNPs for root nodulation and agronomic traits in chickpea

**DOI:** 10.3389/fpls.2024.1395938

**Published:** 2024-10-15

**Authors:** B. S. Chandana, Rohit Kumar Mahto, Rajesh Kumar Singh, Aditi Bhandari, Gitanjali Tandon, K. K. Singh, Sunita Kushwah, Gera Roopa Lavanya, Mir Asif Iquebal, Neelu Jain, Himabindu Kudapa, H. D. Upadhyaya, Aladdin Hamwieh, Rajendra Kumar

**Affiliations:** ^1^ Division of Genetics, Indian Agricultural Statistics Research Institute (ICAR)-Indian Agricultural Research Institute, New Delhi, India; ^2^ School of Biotechnology, Institute of Science, Banaras Hindu University (BHU), Varanasi, India; ^3^ International Crops Research Institute for the Semi-Arid Tropics, Patancheru, Telangana, India; ^4^ Division of Bioinformatics, ICAR-IASRI, New Delhi, India; ^5^ ICAR-Indian Agricultural Research Institute Regional Station, Samstipur, Bihar, India; ^6^ Krishi Vigyan Kendra (KVK), Vaishali, Dr. Rajendra Prasad Central Agriculture University-Pusa, Hajipur, Bihar, India; ^7^ Department of Genetics & Plant Breeding, Sam Higginbottom University of Agriculture, Technology and Sciences (SHUATS), Prayagraj, India; ^8^ Plant Genome Mapping Laboratory, University of Georgia, Athens, Athens, GA, United States; ^9^ International Center for Agriculture Research in the Dry Areas (ICARDA), Giza, Egypt

**Keywords:** association mapping, chickpea, GWAS, nitrogen fixation, nodulation, PVE, pleiotropic

## Abstract

**Introduction:**

The chickpea (Cicer arietinum L.) is well-known for having climate resilience and atmospheric nitrogen fixation ability. Global demand for nitrogenous fertilizer is predicted to increase by 1.4% annually, and the loss of billions of dollars in farm profit has drawn attention to the need for alternative sources of nitrogen. The ability of chickpea to obtain sufficient nitrogen via its symbiotic relationship with *Mesorhizobium ciceri* is of critical importance in determining the growth and production of chickpea.

**Methods:**

To support findings on nodule formation in chickpea and to map the genomic regions for nodulation, an association panel consisting of 271 genotypes, selected from the global chickpea germplasm including four checks at four locations, was evaluated, and data were recorded for nodulation and 12 yield-related traits. A genome-wide association study (GWAS) was conducted using phenotypic data and genotypic data was extracted from whole-genome resequencing data of chickpea by creating a hap map file consisting of 602,344 single-nucleotide polymorphisms (SNPs) in the working set with best-fit models of association mapping.

**Results and Discussion:**

The GWAS panel was found to be structured with sufficient diversity among the genotypes. Linkage disequilibrium (LD) analysis showed an LD decay value of 37.3 MB, indicating that SNPs within this distance behave as inheritance blocks. A total of 450 and 632 stringent marker–trait associations (MTAs) were identified from the BLINK and FarmCPU models, respectively, for all the traits under study. The 75 novel MTAs identified for nodulation traits were found to be stable. SNP annotations of associated markers were found to be related to various genes including a few auxins encoding as well as nod factor transporter genes. The identified significant MTAs, candidate genes, and associated markers have the potential for use in marker-assisted selection for developing high-nodulation cultivars after validation in the breeding populations.

## 1 Introduction

Chickpea (*Cicer arietinum* L.) is a self-pollinated diploid crop with a chromosome number of 2n = 2x = 16, which is grown as an annual crop mainly during the winter season and is the third most important pulse crop globally with a cultivated area of 15.00 million hectares, production of 15.87 million tons, and average productivity of 1.06 t/ha (FAOSTAT, 2023). Chickpea along with other legumes can transform nitrogen from the atmosphere into ammonia through a symbiotic relationship with a rhizobium, *Mesorhizobium ciceri*. The ability of the chickpea to acquire adequate nitrogen through its symbiotic association with *M. ciceri* is essential for promoting growth and facilitating grain yield. Farmers exploit this mutually beneficial interaction with rhizobia to overcome nutrient deficiencies in soils, as these bacteria can supply as much as 97% of a plant’s total nitrogen demand (Peoples and Craswell, 1992). In addition, these symbiotic relationships play a crucial role in replenishing substantial amounts of nitrogen in agricultural soils and thereby decreasing the reliance on expensive fertilizer treatments worldwide (Herridge et al., 2008). Gaining a greater understanding of the aspects that could enhance the advantages of this mutually beneficial relationship would be rewarding in the field of agriculture. Comprehending the relationship between genotype and nodulation in chickpea is crucial for optimizing the advantages of nitrogen fixation and minimizing the need for nitrogenous fertilizers. The whole process of symbiosis and nodulation is quite complex and tightly regulated and still has not been explored at the molecular level in chickpea. Nevertheless, a considerable number of genes associated with the process of nodulation at various stages have been identified in model legumes like *Medicago truncatula* and *Lotus japonicus*, employing a mix of forward and reverse genetics investigations (Roy et al., 2020). Several genes implicated in nodulation were initially discovered as nodulin genes that have elevated expression levels in nodules as compared to other plant tissues. Reverse genetics tests demonstrated that a significant number of these genes encoded proteins that played a role in nodulation (Combier et al., 2006). Precise improvement of complex quantitative traits like root nodulation traits needs the identification of related genomic regions rather than the identification of genes and quantitative trait locus (QTL) mapping, a robust technique that requires either bi-parental mapping populations, which is time-consuming (Edae et al., 2014), or genome-wide association study (GWAS) based on the linkage disequilibrium (LD) for the identification of genes/QTLs. Despite that chickpea is the most important food legume, nodulation studies in chickpea have been limited. Hence, a high-throughput, in-depth analysis of the chickpea root nodule is crucial for gaining deeper insights into the complexities of nodulation events. Identification of genotypes as resources for high nodulation and establishing an association between the nodulation traits and molecular markers can produce a higher yield per unit area. So far, the chickpea germplasm including the global core collection has not been fully utilized for the purpose. Thus, we conducted a systematic evaluation of conserved germplasm to facilitate the identification of high-nodulation genotypes with the objectives of phenotyping of nodulation and yield traits as resources, and we conducted a genome-wide association study to establish the association between the nodulation traits and molecular markers/genomic regions in chickpea.

## 2 Materials and methods

### 2.1 Plant material

A set of 2,094 diverse germplasms including a global core set of 1,950 genotypes and Indian Agricultural Research Institute (IARI) breeding materials (144) of chickpea was evaluated for the number of nodules and yield per se traits. The core germplasms collected from 28 different countries across the world were obtained from the gene bank at the International Crops Research Institute for the Semi-Arid Tropics (ICRISAT), Patancheru, Telangana, India. The plant materials were grown and evaluated for two consecutive crop seasons in 2018–2019 and 2019–2020 at IARI, New Delhi. Phenotypic data for nodulation and yield per se traits were recorded. Data were subjected to core hunter3 in R (De Brucellae et al., 2018) and descriptive statistics for the construction of four association panels (APs) focusing on nodulation (two APs), root (one AP), and plant architecture traits (one AP). The association panel under study consists of 271 diverse germplasm inclusive of BG 372, BG 3022, BG 547, and BG 1105 as four checks (**Supplementary Table 1**). The experimental trials for the association panel were conducted at four environmental locations in 2020–2021, as follows: IARI, New Delhi, location 1 (28°38′24.0252″N latitude, 77°10′26.328″E longitude, and 228.6 m AMSL) having sandy clay loam soils; Sam Higginbottom University of Agriculture, Technology and Sciences (SHUATS), Naini, Prayagraj, location 2 (25°24′41.27″N latitude, 81°51′3.42″E longitude, and 98 m AMSL) with clay loam to sandy loam soil; Dr. Rajendra Prasad Central Agricultural University (RPCAU), Samastipur (KVK, Vaishali), location 3 (25°86′29.679″ latitude, 85°78′10.263″ longitude, and 52 m AMSL) with sandy loam soil; and IARI Regional Station, Pusa, Bihar, location 4 (25°54′56.16″ latitude, 85°40′24.9564″ longitude, and 52 m AMSL) with alluvial soils. The layout embodied an augmented randomized block design with four blocks and a spacing of 60 cm between rows and 10 cm between plants. Each block consisted of 72 lines including repeated check rows. The observations recorded on randomly selected five plants for each genotype for 12 traits included days to 50% flowering (DFF), plant height (PH) in cm, number of pods (NOP) per plant, number of seeds (NOS) per plant and yield (SY) per plant in grams, number of nodules (NON) per plant, nodule fresh weight (NFW) in grams, root fresh weight (RFW) in grams, root dry weight (RDW) in grams, stem fresh weight (SFW) in grams, and stem dry weight (SDW) in grams.

### 2.2 Phenotyping and data analysis

**Figure 1:**
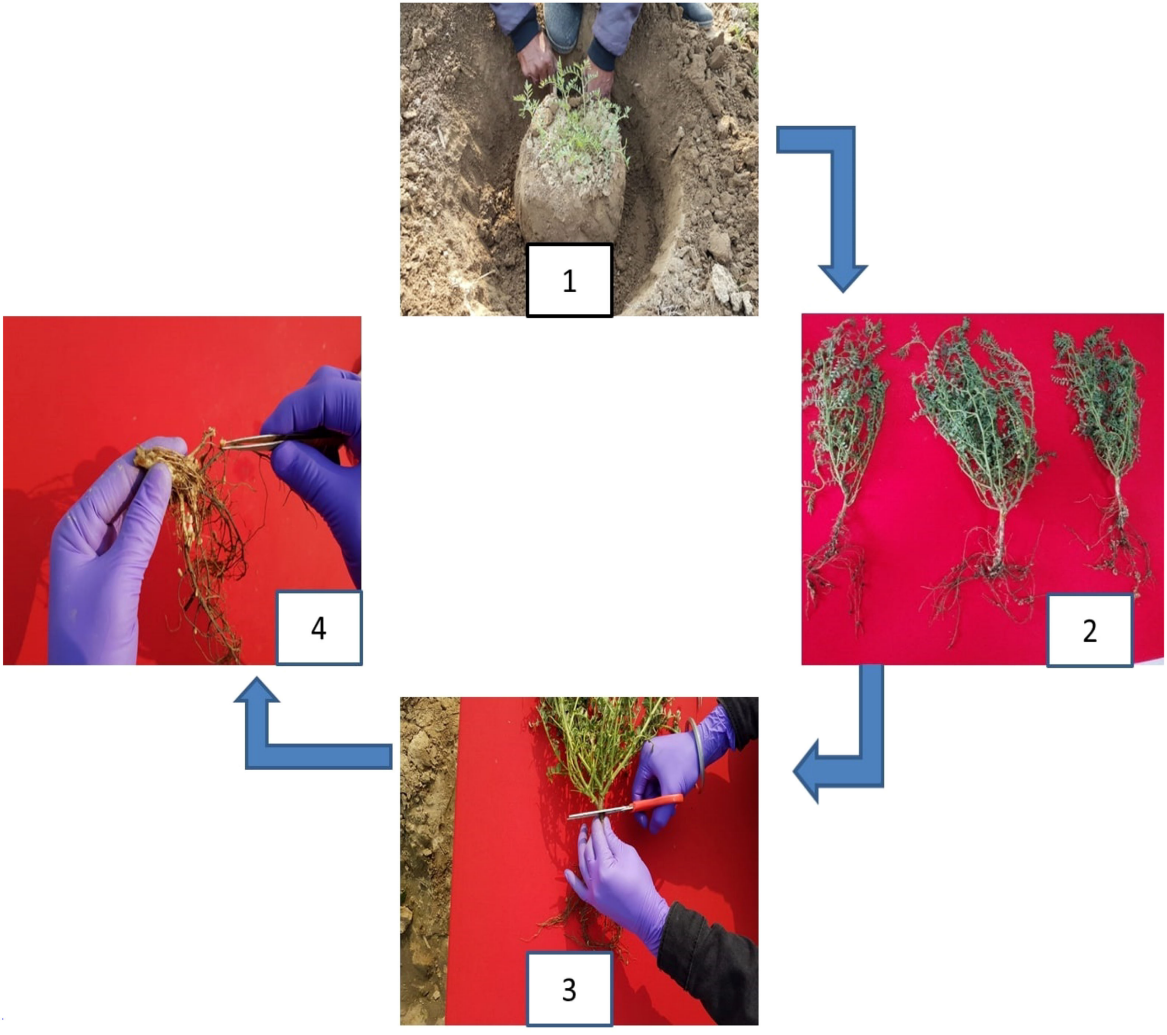
Phenotyping steps for number of nodules.

The nodule phenotyping pipeline includes mainly two parameters: counting the number of nodules per plant and taking nodule fresh weight as explained further. Phenotyping for the number of nodules was conducted 60 days after sowing; the optimum stage in legumes to fix maximum biological nitrogen was as reported earlier (Yuan et al., 2022) and followed the steps shown in **Figure 1**. Randomly selected five plants from each genotype were uprooted from the adhered soil mass using a hand hoe by digging 20 cm or even deeper into the soil (step 1). Particular care was taken not to disturb the root nodule system during sampling, and adhering soil was removed carefully (step 2). Root and shoot systems were separated (step 3). Roots with intact nodules were washed, and the number of nodules was counted (step 4). The intact cleaned roots were stored in butter paper bags to further obtain nodule and root fresh weight. The shoots were also kept in polythene packets. Phenotyping for root, shoot, and nodule fresh weight was conducted on the same day followed by their storage in the oven at 55°C for 1 week to obtain their dry weight. The explained procedure was followed for all the locations, the observations for all the traits were taken for five plants, and the mean of five plants per genotype was taken into consideration for analysis. Phenotypic data analysis including frequency distribution and correlation for all four locations was conducted using the R software (https://www.R-project.org/).

### 2.3 Genotyping of the association panel

Genotypic data for the association panel were successfully obtained from whole-genome resequencing of chickpea (Varshney et al., 2019). For single-nucleotide polymorphism (SNP), called clean reads were mapped on the reference genome of the chickpea genotype CDC Frontier (Varshney et al., 2013). To filter out low-quality variants, the loci with sequencing depth higher than 10,000 and lower than 400, mapping times higher than 1.5, or quality scores lower than 20 were used. The loci with estimated allele frequency not equal to 0 or 1 were determined as SNPs. The raw genotypic data extracted from the database contained 1,198,121 SNPs distributed on eight pseudomolecules. The filtering for missing data (≤20%) and minor allele frequency (MAF) ≥2% was performed using vcf tools (Vogt et al., 2021); an additional filter for the rate of heterozygosity (Ho) ≤ 0.5%, MAF ≥ 5%, and Ho ≤ 5% led to a working set of 602,344 SNPs (referred as 602K), which were used for genome-wide association mapping analysis.

### 2.4 Association analysis

The generated genotyping data were integrated with phenotypic data of multi-location observations recorded for the traits under study. Phenotypic data were used for the calculation of best linear unbiased predictions (BLUPs). Individual BLUPs across the environments were estimated using the ACBD-R software (Rodríguez et al., 2017) with the following model:


Yij=μ+Genj+Envi+Envi×Genj+Bloc(Envi)+eij

where Genj and Checkj correspond to the effects of the identifier of checks, the un-replicated genotypes, and checks that are repeated in each block (Blocki); Envi is the effect of ith environment, µ is the mean, and e is the error component (as described in ACBD-R User Manual; Rodríguez et al., 2017). The population structure was assessed using a neighbor-joining phylogenetic tree (constructed through the TASSEL software and visualized through the ITOL software) and principal component analysis (PCA). PCA was performed using a function dedicated to assessing the genetic relatedness among accessions and generating the principal components (PCs) from the genotypic data. The first three principal components were considered as covariates in GAPIT using the high-performance computing R tool. The r^2^ values for SNP markers were computed and then filtered focusing on pairs within each chromosome, and a linkage disequilibrium heat map was created to identify significant LD block and its size, which falls diagonally in the heat map at a p-value of 0.001. A whole genome was generated and sorted for individual chromosomes by utilizing TASSEL version 5. Subsequently, these files were used to generate LD decay curves for all eight chromosomes individually and for the entire genome. To estimate the sizes of LD blocks, the r^2^ values were plotted against the distance in base pairs (bp) while setting a threshold at r^2^ = 0.2.

GWAS was performed using the general linear model (GLM), mixed linear model (MLM), multi-locus mixed model (MLMM), FarmCPU model, and BLINK model using the R/GAPIT 3.0 package. Further, in this study, the Bonferroni correction threshold value of −log10 > 7.0 (p-value) was used as the cutoff. The SNPs with the above values were declared as significant marker–trait associations (MTAs). The Manhattan and Q-Q plots were generated through qqman version 0.1.8 (Turner, 2014). The percent phenotypic variance (PV) explained by all significant detected SNPs was generated from all used models and calculated as the squared correlation between the phenotype and genotype of the SNP. Stable MTAs obtained more than twice across the location were found. Pleotropic SNPs having an association with more than one trait were also identified.

### 2.5 Identification of associated SNPs and candidate genes

The genes involving significant SNP markers were aligned against the National Center for Biotechnology Information (NCBI) non-redundant (nr) protein database using BLASTX to obtain functional annotations (https://blast.ncbi.nlm.nih.gov). The stable and pleiotropic SNPs were subjected to a basic local alignment search tool (BLAST) search using the sequence information of the markers. A BLAST search was carried out using a data web service. Putative candidate transcripts (with transcript IDs) within and 20-kb flanking region of SNPs were identified in the NCBI chickpea database, and the function of the gene was determined using the UniProt database (https://www.uniprot.org/).

## 3 Results

### 3.1 Distribution and correlation among the nodulation traits

**Figure 2:**
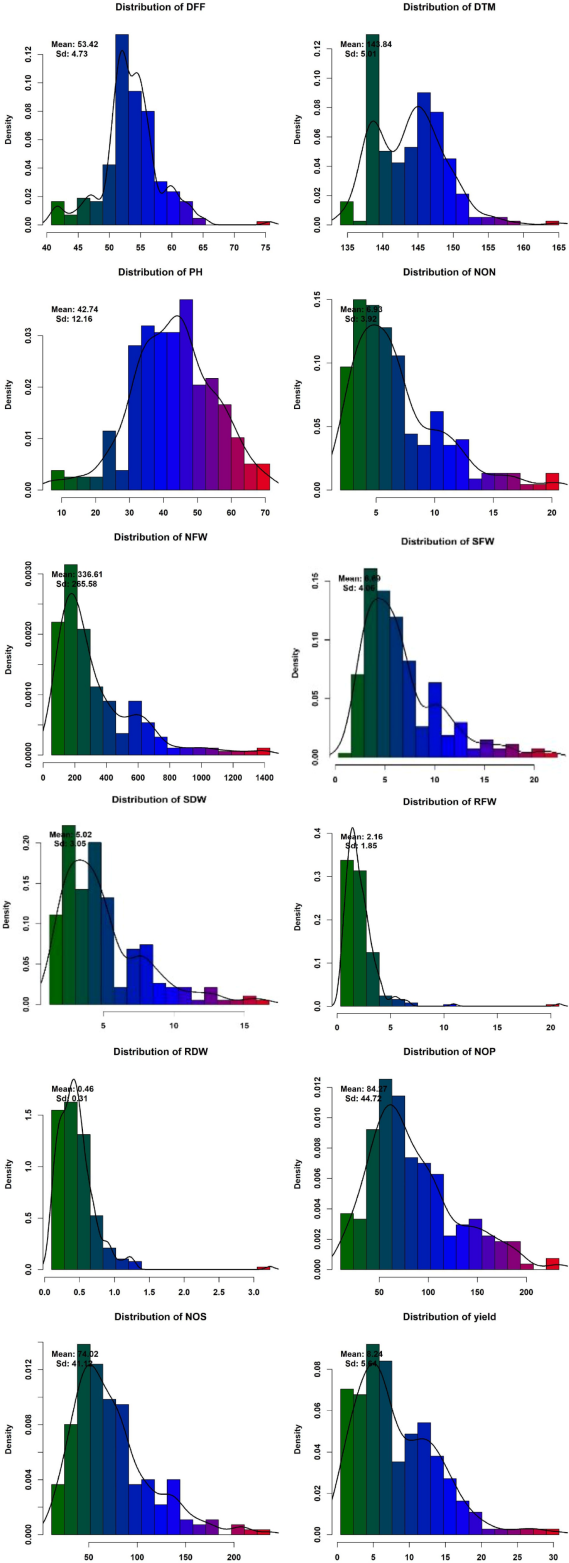
Phenotypic variation for traits assayed within the chickpea reference set.

**Figure 3:**
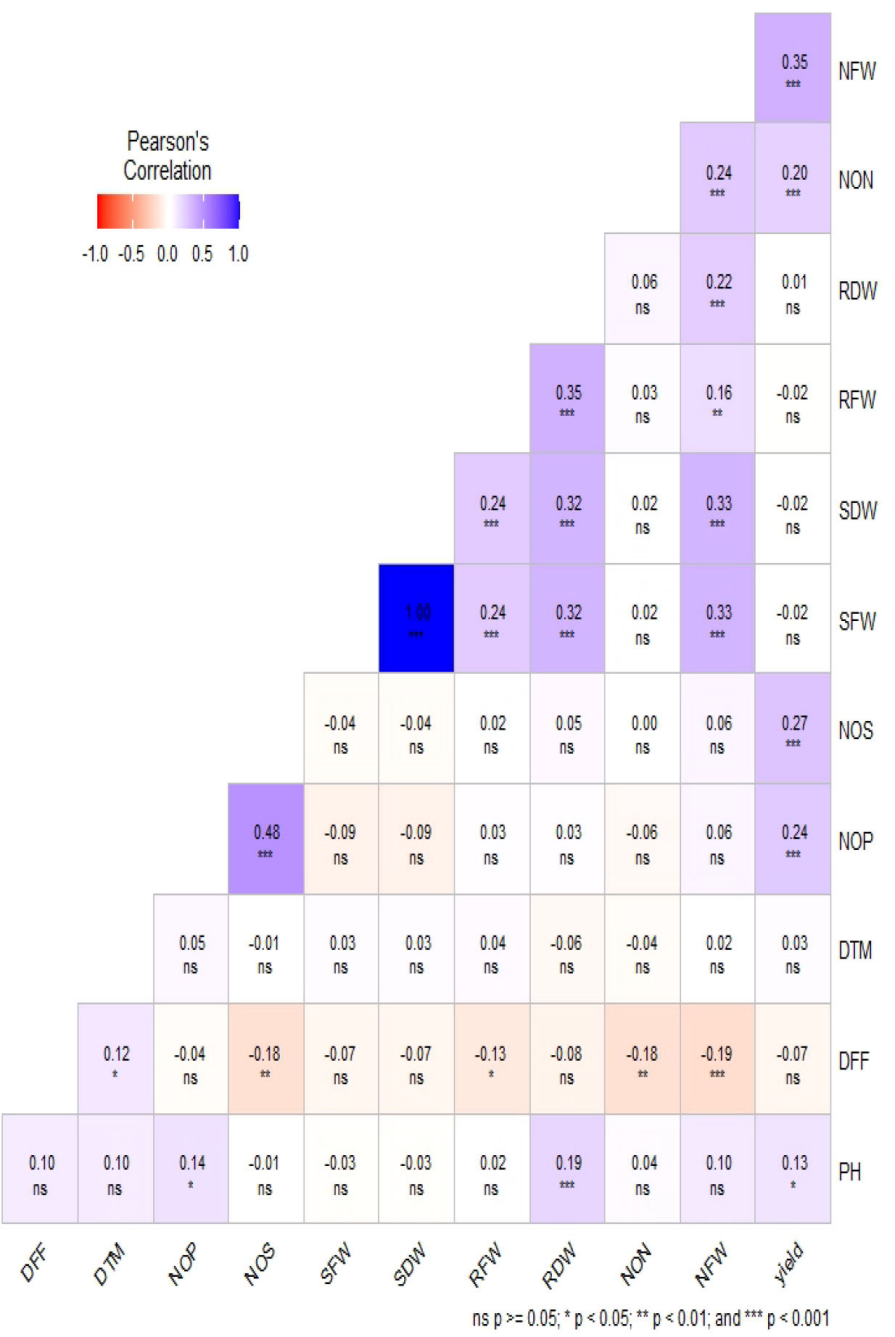
Estimation of Pearson’s correlation coefficients for the chickpea association panel.

Phenotypic data collected under all the environments for the association panel were statically analyzed, and the results are presented further. The mean and distributions for 12 phenotypic variables in 271 accessions chosen from the chickpea reference set are presented in **Figure 2**. The traits under study exhibited normal and near-normal to skewed distributions. The mean of the DFF (53.42), Days to maturity (DTM) (143.84), PH (42.74), NOP (84.27), NOS (74.32), yield per plant (8.24), NON (8.83), NFW (336.61), RFW (2.16), RDW (0.46), SFW (6.69), and SDW (4.23) were recorded. The results of correlation coefficients revealed that nodule fresh weight and nodule dry weight were positively and significantly correlated with yield plant^−1^ at genotypic and phenotypic levels (**Figure 3**).

The results of the correlation coefficients evidenced that the genotypic correlations for most of the traits were slightly higher than their corresponding phenotypic correlations, which would be beneficial in the selection of traits because they exclude the environmental influence. It also revealed significant and positive correlation values for seed yield with the number of pods per plant, seeds per plant, shoot fresh weight, days to flowering, and days to maturity. However, negative correlation values for seed yield with shoot dry weight and nodule fresh weight were observed. Significant and positive correlations were observed for the trait SY with NPB, NSB, and NPP, indicating that the seed yield may be enhanced through an increase in Number of primary branches (NPB), Number of secondary branches (NSB), and Number of pods per plant (NPP).

### 3.2 Assessment of population structure and linkage disequilibrium block

**Figure 4:**
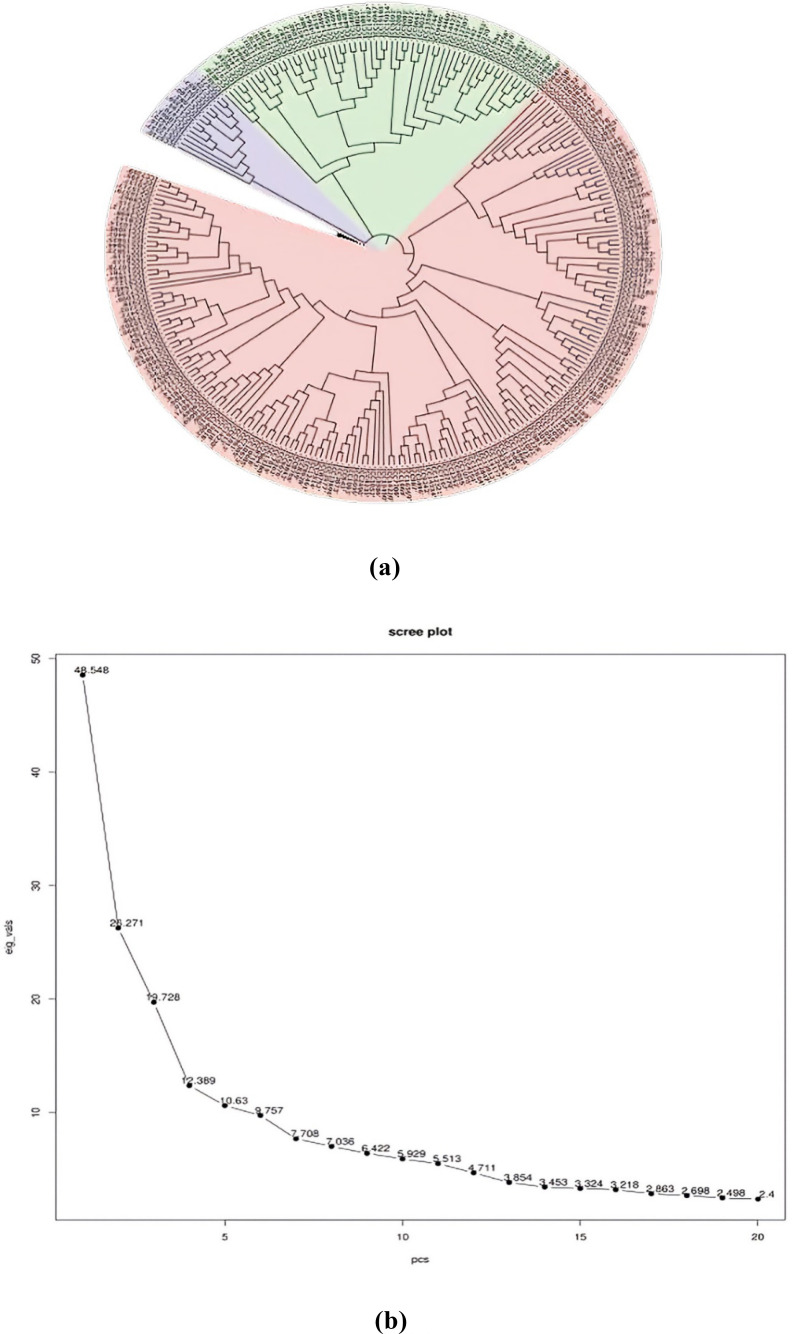
**(A)** Phylogenetic tree. **(B)** PCA scree plot. PCA, principal component analysis.

In order to assess the number of subpopulations, a phylogenetic tree utilizing phenotypic and marker data through the neighbor-joining method was constructed (**Figure 4A**). The phylogenetic tree revealed the presence of three subpopulations/subclusters, which were further confirmed by the generated PCA scree plot (**Figure 4B**). Subcluster 3 was the largest one, containing 202 inclusive of all checks, followed by subcluster 2 containing 55 and subcluster 1 containing 14 genotypes. Subclusters 1 and 2 remained confined to ICC series germplasm lines.

**Figure 5:**
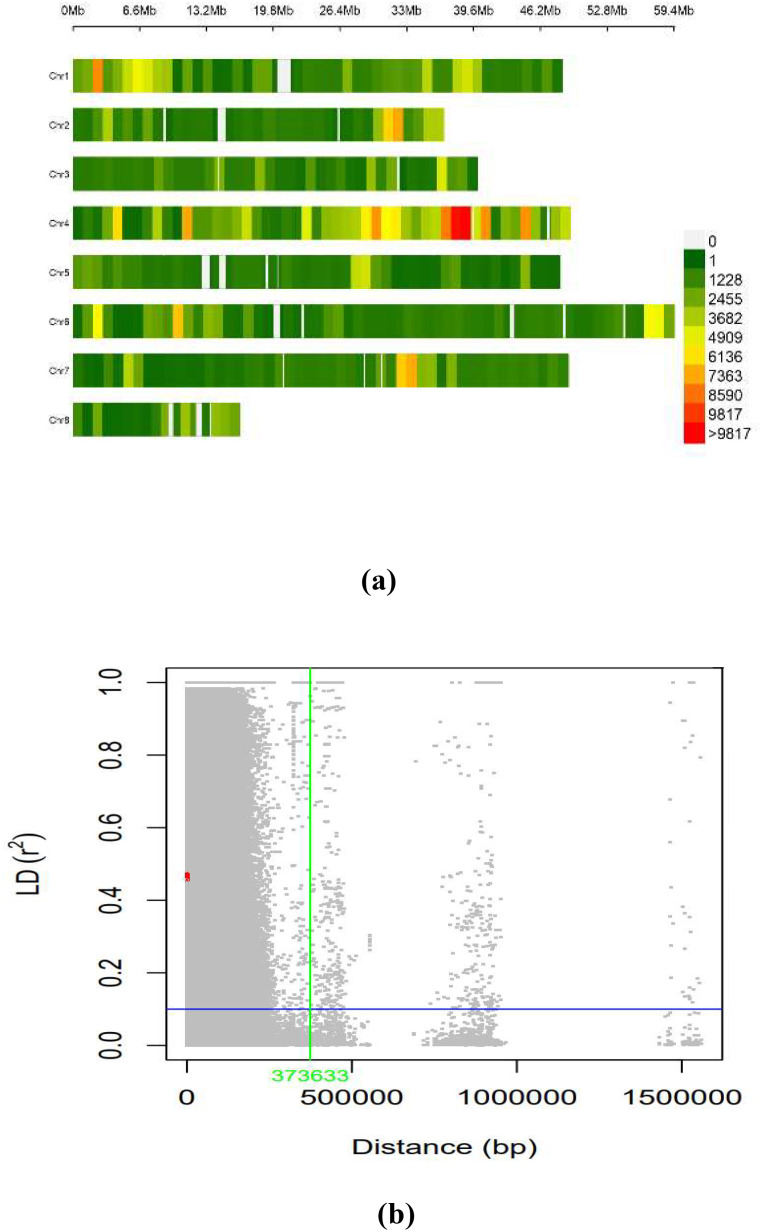
**(A)** SNP density plot indicating distribution of filtered SNPs across the chromosomes. **(B)** Linkage disequilibrium measured r^2^ plotting *vs*. physical distance between pairs of markers (Plink 1.9). SNP, single-nucleotide polymorphism.

The LD across the genome was estimated using 603,100 SNPs from the working set through the TASSEL software for the whole genome. The average LD across the genome was 635.9 kb. The distribution of SNPs across the chromosome was on eight different pseudomolecules in chickpea, as presented in **Figure 5A**. The number of SNPs available on each pseudomolecule and the number of SNPs used for conducting marker–trait association are represented in **Figure 5B**. The genomic regions represented in dark red on the chromosome were found to have a high density of SNPs, and the genomic regions represented in green had low SNP density. Ca 4 had the highest number of SNPs, and Ca 8 contained the least number of SNPs.

### 3.3 Genome-wide association study for identification of MTAs for nodulation and agronomic traits

A GWAS analysis was carried out to identify SNPs associated with investigated variables in chickpeas, including nodulation, morphological, and yield traits. The GWAS identified total SNPs through BLINK (643), FarmCPU (720), and MLM (439) models; the number of significant identified SNPs for each trait under different models in four different conditions are listed in **Table 1**; additional SNPs identified for each trait, along with their chromosome, position and Phenotypic variance explained (PVE) identified through the Blink model, FarmCPU model, and MLM are given in **Supplementary Tables 2**-**11**. As the current study focused on nodulation traits number of nodules and nodule fresh weight, their stable SNPs are presented in **Table 2**, and Manhattan and Q-Q plots are represented in **Supplementary Figures 1** and **2**. We mainly considered SNPs that had a p-value threshold of −log10 p-value ≥ 6 and a false discovery rate (FDR) below 0.1. The GWAS for the trait NON found eight SNPs that are stably expressible at locations 1 and 2; one SNP that is stably expressible at locations 1, 2, and 3; and one SNP that is stably expressible at locations 1, 2, and 4 (**Table 2**). Among these identified MTAs, SNP 2_825902 had 27.33% of PVE. Marker–trait association of nodule fresh weight resulted in the identification of SNP markers as 20 in the FarmCPU model; among the significant identified markers, seven SNP markers were found above the threshold of −log10 p-value. SNPs Ca5pos20514758.1 and Ca7pos21461047.1 presented on chromosome numbers 5 and 7 had 42.28% and 9.31% of PVE, respectively, through FarmCPU.

**Table 1:** List of the significant SNP markers identified using different models.

Trait	Blink		FarmCPU		MLM	
	SNP	-Log 10 (p)	SNP	-Log 10 (p)	SNP	-Log 10 (p)
**DFF**	88	4 to 8	76	4 to 8	57	4 to 8
**DTM**	76	4 to 8	80	4 to 8	60	4 to 8
**PH**	57	4 to 6	72	4 to 6	29	4 to 6
**NON**	12	7 to 14	2	7 to 14	3	7 to 14
**NFW**	15	7 to 14	15	7 to 14	4	7 to 14
**RFW**	60	4 to 8	80	4 to 8	75	4 to 8
**RDW**	58	4 to 8	78	4 to 8	72	4 to 8
**SFW**	35	4 to 8	37	4 to 8	20	4 to 8
**SDW**	22	4 to 8	45	4 to 8	19	4 to 8
**NPP**	70	4 to 8	80	4 to 8	35	4 to 8
**NSP**	80	4 to 8	80	4 to 8	30	4 to 8
**SY**	70	4 to 8	75	4 to 8	35	4 to 8
**Total**	643		720		439	

The list of all the identified markers along with chromosome location, PVE, and position of the markers are given in **Supplementary Tables 2**-**11**, and Manhattan plots are given in **Supplementary Figures 1**-**10**.

DFF, days to 50% flowering; PH, plant height (cm); PB, primary branches; SB, secondary branches; NOP, number of pods per plant; NOS, number of seeds per plant; Yield, yield per plant (g); NON, number of nodules per plant; NFW, nodule fresh weight (g); RFW, root fresh weight (g); RDW, root dry weight (g); SFW, stem fresh weight (g); SDW, stem dry weight (g); SNP, single-nucleotide polymorphism.

**Table 2:** Significant marker–trait associations at Bonferroni correction p-value for traits under study at different locations/environments.

Trait	SNP ID	Chromosome	Position (bp)	p-Value	MAF	Location
NON	Ca2pos2169123.1	2	2169123	4.22E−08	0.00369	1
	Ca2pos28892511.1	2	2.90E+07	6.33E−15	0.09963	1 and 2
	Ca2pos28892511.1	2	2.90E+07	5.96E−09	0.09963	1 and 2
	Ca3pos36960027.1	3	3.70E+07	2.64E−08	0.04982	1 and 2
	Ca4pos43172963.1	4	4.30E+07	5.61E−12	0.02768	1 and 2
	Ca4pos43172963.1	4	4.30E+07	1.63E−09	0.02768	1, 2, and 4
	Ca5pos2026241.1	5	2026241	5.86E−15	0.49631	1 and 2
	Ca5pos21727560.1	5	2.20E+07	3.26E−14	0.49815	1, 2, and 3
	Ca5pos21727560.1	5	2.20E+07	9.49E−18	0.49815	1 and 2
	Ca5pos38432936.1	5	3.80E+07	4.22E−08	0.49815	1 and 2
	Ca7pos32196399.1	7	3.20E+07	3.26E−10	0.04428	1 and 2
	Ca7pos47087116.1	7	4.70E+07	7.67E−08	0.19202	2
NFW	Ca7pos33694339.1	7	3.40E+07	6.25E−14	0.15926	1 and 3
FarmCPU	Ca3pos8244189.1	3	8244189	8.44E−11	0.13889	1 and 3
	Ca8pos11345025.1	8	1.10E+07	1.51E−08	0.09815	1 and 3
	Ca6pos56503783.1	6	5.70E+07	4.85E−08	0.0037	1 and 3
	Ca2pos34684339.1	2	3.50E+07	5.23E−08	0.02222	1 and 3
	Ca4pos40834901.1	4	4.10E+07	5.52E−08	0.21482	1 and 3
	Ca6pos43064226.1	6	4.30E+07	1.34E−07	0.20185	1 and 3
	Ca4pos4851043.1	4	4851043	3.65E−07	0.04259	1 and 3
	Ca3pos13147329.1	3	1.30E+07	5.01E−07	0.40185	1 and 3
	Ca8pos12988095.1	8	1.30E+07	1.55E−06	0.4963	1 and 4
	Ca8pos11435804.1	8	1.10E+07	2.94E−06	0.04815	1 and 4
	Ca7pos21461047.1	7	2.10E+07	3.85E−06	0.39815	1, 2, and 4
	Ca4pos2034216.1	4	2034216	4.22E−06	0.49259	1, 2, and 4
	Ca5pos20514758	5	3.30E+07	7.71E−06	0.33704	1, 2, and 4
	Ca4pos29978493.1	4	3.00E+07	9.61E−06	0.09074	1 and 4
	Ca3pos8244095.1	3	8244095	9.64E−06	0.05926	1 and 3

MAF, minor allele frequency; SNP, single-nucleotide polymorphism.

The list of the stable SNPs and their location along with their trait are in **Table 3**. Stable SNPs are SNPs that were identified in more than one environment. Most of the SNPs were stably expressive and common for location 3 *vs*. 4 and location 1 *vs.* 3. For plant height, we found 57, 72, and 29 SNPs in the BLINK model, FarmCPU model, and MLMM, respectively. Among the identified markers, we report 12 stable SNPs. SNP Ca6pos1492432.1 (FarmCPU for location 1) found on chromosome number 6 had 25.98% of PVE, and SNP Ca2pos6782498.2 (BLINK, location 3) present on chromosome number 2 had 27.24% of PVE (**Supplementary Tables 2**-**10**). SNP Ca8pos11994085.1 present on chromosome 8 had 14.56% of PVE (the SNPs along with PVE% are given in **Supplementary Tables 2**-**11**). We identified 14 stable SNPs each for DFF, DTM, and PH. The stable SNPs for DFF were identified only from the MLM; however, for DTM and plant height, stable SNPs were common across the models (MLM, BLINK, and FarmCPU), and for all three traits, we observed stable SNPs at locations 3 and 4. Among these MTAs, the stable SNP for the trait DFF was Ca6pos47821883.1, present on chromosome number 6 and had 48.43% of PVE (MLM and GLM). SNP Ca1pos4393831.1 present on chromosome number 1 had 8.82% of PVE. SNP Ca5pos30670011.1 present on chromosome number 5 had 9.15% of Phenotypic variance explained (PVE). The trait DTM contained SNPs Ca1pos9168435.1 and Ca1pos11296743.1 present on chromosome number 1, which had 42.37% and 33.81% of PVE, respectively; SNPs Ca5pos13855141.1 and Ca5pos30670011.1 present on chromosome 5 number had 8.05% and 9.15% of PVE, respectively. For the traits SFW and NOS, we identified 20 stable SNPs. SNPs Ca1pos33781183.1 and Ca1pos35026875.1 identified for trait SFW had 8.91% and 3.7% of PVE, respectively. SNPs Ca5pos30670011.1, Ca3pos19977000.1, and Ca4pos27656718.1 identified for NOS had 9.15%, 7.04%, and 3.74% of PVE, respectively.

**Table 3:** Stable SNPs associated with more than one environment.

DFF					
SNP	Linkage group	Position	p-Value	Model	Location
Ca1pos24610010.1	1	24610010	7.50E−05	MLM	3 and 4
Ca2pos1198412.1	2	1198412	1.35E−05	MLM	3 and 4
Ca2pos8664225.1	2	8664225	8.93E−05	MLM	3 and 4
Ca3pos12432325.1	3	12432325	8.59E−05	MLM	3 and 4
Ca3pos30297117.1	3	30297117	7.05E−05	MLM	3 and 4
Ca6pos16026122.1	6	16026122	8.45E−05	MLM	3 and 4
Ca6pos41345425.1	6	41345425	9.11E−05	MLM	3 and 4
Ca6pos41972856.1	6	41972856	7.30E−05	MLM	3 and 4
Ca6pos41972896.1	6	41972896	8.50E−06	MLM	3 and 4
Ca6pos48048350.1	6	48048350	8.38E−05	MLM	3 and 4
Ca7pos13030083.1	7	13030083	5.33E−05	MLM	3 and 4
Ca7pos19732025.1	7	19732025	3.66−05	MLM	3 and 4
Ca7pos38107103.1	7	38107103	4.55E−05	MLM	3 and 4
Ca7pos43994019.1	7	43994019	9.52E−05	MLM	3 and 4

DTM					
SNP	Chromosome	Position	p-Value	Model	Location
Ca1pos29737724.1	1	29737724	1.55E−05	MLM, BLINK, and FarmCPU.	3 and 4
Ca1pos29737735.1	1	29737735	1.83E−05		3 and 4
Ca3pos433121.1	3	433121	0.00010207		3 and 4
Ca3pos10956124.1	3	10956124	0.00011024		3 and 4
Ca3pos13147329.1	3	13147329	3.76E−05		3 and 4
Ca5pos29910669.1	5	29910669	7.19E−05		3 and 4
Ca5pos29910672.1	5	29910672	1.21E−05		3 and 4
Ca6pos32512792.1	6	32512792	6.62E−05		3 and 4
Ca6pos34164845.1	6	34164845	6.28E−05		3 and 4
Ca6pos49267149.1	6	49267149	6.13E−05		3 and 4
Ca6pos56003699.1	6	56003699	9.10E−05		3 and 4
Ca6pos58002384.1	6	58002384	2.65E−07		3 and 4
Ca7pos30208448.1	7	30208448	9.94E−05		3 and 4
Ca7pos37819116.1	7	37819116	2.06E−05		3 and 4

Plant height					
SNP	Chromosome	Position	p-Value	Model	Location
Ca1pos27773061.1	1	27773061	8.25E−05	MLM and FarmCPU	3 and 4
Ca2pos20166203.1	2	20166203	9.11E−05	MLM and FarmCPU	3 and 4
Ca3pos2766368.1	3	2766368	6.92E−05	MLM	3 and 4
Ca4pos8732683.1	4	8732683	7.41E−05	MLM	3 and 4
Ca4pos8818268.1	4	8818268	2.51E−05	MLM and FarmCPU	3 and 4
Ca4pos9195726.1	4	9195726	2.20E−05	MLM and FarmCPU	3 and 4
Ca4pos35809754.1	4	35809754	6.44E−05	MLM	3 and 4
Ca5pos1941712.1	5	1941712	6.74E−05	MLM	3 and 4
Ca5pos5415849.1	5	5415849	3.72E−05	MLM	3 and 4
Ca6pos42565470.1	6	42565470	5.02E−05	MLM	3 and 4
Ca6pos47675915.1	6	47675915	1.36E−05	MLM	3 and 4
Ca6pos47675923.1	6	47675923	3.74E−05	MLM and FarmCPU	3 and 4
Ca6pos52464995.1	6	52464995	5.38E−05	MLM and FarmCPU	3 and 4
Ca7pos30981168.1	7	30981168	9.33E−05	MLM	3 and 4
Ca7pos40493709.1	7	40493709	4.56E−05	MLM and FarmCPU	3 and 4

SFW					
Ca1pos3076296.1	1	3076296	2.81E−05	MLM	3 and 4
Ca1pos3105289.1	1	3105289	4.73E−05	MLM	3 and 4
Ca1pos37122468.1	1	37122468	2.91E−05	MLM	3 and 4
Ca1pos39647846.1	1	39647846	1.54E−05	MLM	3 and 4
Ca2pos28823139.1	2	28823139	4.73E−05	MLM	3 and 4
Ca3pos19707707.1	3	19707707	3.93E−05	MLM	3 and 4
Ca3pos25805689.1	3	25805689	6.09E−05	MLM	3 and 4
Ca3pos25805690.1	3	25805690	6.09E−05	MLM	3 and 4
Ca4pos32772485.1	4	32772485	2.04E−05	MLM	3 and 4
Ca5pos33032029.1	5	33032029	3.79E−05	MLM	3 and 4
Ca5pos41523197.1	5	41523197	6.74E−05	MLM	3 and 4
Ca5pos42012947.1	5	42012947	3.52E−05	MLM	3 and 4
Ca5pos42074441.1	5	42074441	1.16E−05	MLM	3 and 4
Ca5pos42272729.1	5	42272729	2.09E−05	MLM	3 and 4
Ca6pos14512673.1	6	14512673	2.23E−05	MLM	3 and 4
Ca6pos27009414.1	6	27009414	5.12E−05	BLINK and FarmCPU	3 and 4
Ca7pos15917660.1	7	15917660	2.39E−05	BLINK and FarmCPU	3 and 4
Ca7pos30666345.1	7	30666345	2.04E−05	BLINK and FarmCPU	3 and 4
Ca8pos12985289.1	8	12985289	4.32E−05	BLINK and FarmCPU	3 and 4

NOS					
Ca1pos8737037.1	1	8737037	1.46E−05	BLINK and FarmCPU	1 and 3
Ca1pos8820515.1	1	8820515	4.41E−05	BLINK and FarmCPU	1 and 3
Ca1pos14062422.1	1	14062422	3.50E−06	BLINK and FarmCPU	1 and 3
Ca1pos14070458.1	1	14070458	5.92E−06	BLINK and FarmCPU	1 and 3
Ca1pos46718466.1	1	46718466	3.87E−05	BLINK and FarmCPU	1 and 3
Ca2pos11422406.1	2	11422406	2.13E−05	BLINK, FarmCPU, and MLM	1 and 3
Ca2pos11422414.1	2	11422414	3.55E−05	BLINK, FarmCPU, and MLM	1 and 3
Ca3pos3042321.1	3	3042321	3.86E−05	BLINK and FarmCPU	1 and 3
Ca3pos39844991.1	3	39844991	9.40E−06	BLINK and FarmCPU	1 and 3
Ca3pos39885397.1	3	39885397	1.26E−05	BLINK and FarmCPU	1 and 3
Ca4pos19410981.1	4	19410981	8.00E−06	BLINK and FarmCPU	1 and 3
Ca5pos3834038.1	5	3834038	7.49E−06	BLINK and FarmCPU	1 and 3
Ca5pos46549190.1	5	46549190	8.46E−06	BLINK, FarmCPU, and MLM	1 and 3
Ca6pos24093514.1	6	24093514	2.61E−05	BLINK and FarmCPU	1 and 3
Ca6pos28350598.1	6	28350598	3.44E−05	BLINK and FarmCPU	1 and 3
Ca6pos53644742.1	6	53644742	3.20E−05	BLINK and FarmCPU	1 and 3
Ca7pos27650319.1	7	27650319	4.45E−05	BLINK, FarmCPU, and MLM	1 and 3
Ca7pos47164247.1	7	47164247	1.35E−05	BLINK and FarmCPU	1 and 3
Ca8pos13311157.1	8	13311157	1.15E−05	BLINK and FarmCPU	1 and 3

SNP, single-nucleotide polymorphism; DFF, days to 50% flowering; SFW, stem fresh weight (g); NOS, number of seeds per plant.

### 3.5 SNP markers associated with two or more different traits

An important initial step in the process of revealing pleiotropic loci associated with complex phenotypes is to examine SNPs that have already been independently associated with one or more different traits using the statistically stringent GWAS framework. The GWAS results were fully examined in order to identify markers that were common between traits (**Table 4**). A total of 43 SNPs were found common, with notable p-values and statistically significant FDRs that reduce the chances of false associations and increase the chances of true association discovered in this study. There were four markers found common for RFW *vs*. RDW and 16 markers for SFW *vs*. SDW, and 13 markers were present on chromosome 3 between genomic regions 37990876 and 38114728. Nineteen markers were common for NPP *vs*. Number of seeds per plant (NSP) and were distributed across all eight chromosomes of the chickpea.

**Table 4:** List of the common SNP markers found for more than one trait (pleiotropic SNPs).

	SNP	Chromosome	Position	p-Value	Location	Traits
1	Ca1pos34151789.1	1	34151789	6.68E−05	3 and 4	RFW and RDW filtering criteria
2	Ca6pos38012786.1	6	38012786	4.43E−05	3 and 4	RFW and RDW
3	Ca7pos19898936.1	7	19898936	6.01E−05	3 and 4	RFW and RDW
4	Ca7pos22479713.1	7	22479713	3.20E−06	3 and 4	RFW and RDW
5	Ca1pos2169207.1	1	2474918	1.23E−06	3 and 4	SFW and SDW
6	Ca1pos2474918.1	1	40820287	1.69E−06	3 and 4	SFW and SDW
7	Ca1pos40820287.1	1	37940720	3.46E−06	3 and 4	SFW and SDW
8	Ca3pos37940720.1	3	37990876	9.19E−06	3 and 4	SFW and SDW
9	Ca3pos37990876.1	3	38008999	5.25E−06	3 and 4	SFW and SDW
10	Ca3pos38057183.1	3	38057193	1.85E−06	3 and 4	SFW and SDW
11	Ca3pos38057193.1	3	38057228	7.16E−06	3 and 4	SFW and SDW
12	Ca3pos38057228.1	3	38063432	8.30E−06	3 and 4	SFW and SDW
13	Ca3pos38063432.1	3	38063441	4.21E−06	3 and 4	SFW and SDW
14	Ca3pos38063441.1	3	38068021	7.06E−06	3 and 4	SFW and SDW
15	Ca3pos38068021.1	3	38080272	4.01E−06	3 and 4	SFW and SDW
16	Ca3pos38080272.1	3	38100180	2.29E−06	3 and 4	SFW and SDW
17	Ca3pos38100180.1	3	38100212	5.60E−06	3 and 4	SFW and SDW
18	Ca3pos38100212.1	3	38106616	7.44E−06	1, 3, and 4	SFW and SDW
19	Ca3pos38106616.1	3	38114728	7.45E−07	1, 3, and 4	SFW and SDW
20	Ca3pos38114728.1	3	6667809	8.90E−06	1, 3, and 4	SFW and SDW
21	Ca7pos6667809.1	7	46119789	4.42E−06	1, 3, and 4	SFW and SDW
22	Ca1pos8737036.1	1	8737036	1.46E−05	1 and 3	NSP and NPP
23	Ca1pos8737037.1	1	8737037	1.46E−05	1 and 3	NSP and NPP
24	Ca1pos8820515.1	1	8820515	4.41E−05	1 and 3	NSP and NPP
25	Ca1pos14062422.1	1	14062422	3.50E−06	1 and 3	NSP and NPP
26	Ca1pos14070458.1	1	14070458	5.92E−06	1 and 3	NSP and NPP
27	Ca1pos46718466.1	1	46718466	3.87E−05	1 and 3	NSP and NPP
28	Ca2pos11422406.1	2	11422406	2.13E−05	1, 2, and 4	NSP and NPP
29	Ca2pos11422414.1	2	11422414	3.55E−05	1, 2, and 4	NSP and NPP
30	Ca3pos3042321.1	3	3042321	3.86E−05	1, 2, and 4	NSP and NPP
31	Ca3pos39844991.1	3	39844991	9.40E−06	1, 2, and 4	NSP and NPP
32	Ca3pos39885397.1	3	39885397	1.26E−05	1, 2, and 4	NSP and NPP
33	Ca4pos19410981.1	4	19410981	8.00E−06	1, 2, and 4	NSP and NPP
34	Ca5pos3834038.1	5	3834038	7.49E−06	1, 2, and 4	NSP and NPP
35	Ca5pos46549190.1	5	46549190	8.46E−06	1, 2, and 4	NSP and NPP
36	Ca6pos24093514.1	6	24093514	2.61E−05	3 and 4	NSP and NPP
37	Ca6pos28350598.1	6	28350598	3.44E−05	3 and 4	NSP and NPP
38	Ca6pos53644742.1	6	53644742	3.20E−05	3 and 4	NSP and NPP
39	Ca7pos27650319.1	7	27650319	4.45E−05	3 and 4	NSP and NPP
40	Ca7pos47164247.1	7	47164247	1.35E−05	3 and 4	NSP and NPP
41	Ca8pos13311157.1	8	13311157	1.15E−05	3 and 4	NSP and NPP
	SNP	Chromosome	Position	p-Value	Location	Traits
1	Ca1pos34151789.1	1	34151789	6.68E−05	3 and 4	RFW and RDW
2	Ca6pos38012786.1	6	38012786	4.43E−05	3 and 4	RFW and RDW
3	Ca7pos19898936.1	7	19898936	6.01E−05	3 and 4	RFW and RDW
4	Ca7pos22479713.1	7	22479713	3.20E−06	3 and 4	RFW and RDW
5	Ca1pos2169207.1	1	2474918	1.23E−06	3 and 4	SFW and SDW
6	Ca1pos2474918.1	1	40820287	1.69E−06	3 and 4	SFW and SDW
7	Ca1pos40820287.1	1	37940720	3.46E−06	3 and 4	SFW and SDW
8	Ca3pos37940720.1	3	37990876	9.19E−06	3 and 4	SFW and SDW
9	Ca3pos37990876.1	3	38008999	5.25E−06	3 and 4	SFW and SDW
10	Ca3pos38057183.1	3	38057193	1.85E−06	3 and 4	SFW and SDW
11	Ca3pos38057193.1	3	38057228	7.16E−06	3 and 4	SFW and SDW
12	Ca3pos38057228.1	3	38063432	8.30E−06	3 and 4	SFW and SDW
13	Ca3pos38063432.1	3	38063441	4.21E−06	3 and 4	SFW and SDW
14	Ca3pos38063441.1	3	38068021	7.06E−06	3 and 4	SFW and SDW
15	Ca3pos38068021.1	3	38080272	4.01E−06	3 and 4	SFW and SDW
16	Ca3pos38080272.1	3	38100180	2.29E−06	3 and 4	SFW and SDW
17	Ca3pos38100180.1	3	38100212	5.60E−06	3 and 4	SFW and SDW
18	Ca3pos38100212.1	3	38106616	7.44E−06	1, 3, and 4	SFW and SDW
19	Ca3pos38106616.1	3	38114728	7.45E−07	1, 3, and 4	SFW and SDW
20	Ca3pos38114728.1	3	6667809	8.90E−06	1, 3, and 4	SFW and SDW
21	Ca7pos6667809.1	7	46119789	4.42E−06	1, 3, and 4	SFW and SDW
22	Ca1pos8737036.1	1	8737036	1.46E−05	1 and 3	NSP and NPP
23	Ca1pos8737037.1	1	8737037	1.46E−05	1 and 3	NSP and NPP
24	Ca1pos8820515.1	1	8820515	4.41E−05	1 and 3	NSP and NPP
25	Ca1pos14062422.1	1	14062422	3.50E−06	1 and 3	NSP and NPP
26	Ca1pos14070458.1	1	14070458	5.92E−06	1 and 3	NSP and NPP
27	Ca1pos46718466.1	1	46718466	3.87E−05	1 and 3	NSP and NPP
28	Ca2pos11422406.1	2	11422406	2.13E−05	1, 2 and 4	NSP and NPP
29	Ca2pos11422414.1	2	11422414	3.55E−05	1, 2 and 4	NSP and NPP
30	Ca3pos3042321.1	3	3042321	3.86E−05	1, 2 and 4	NSP and NPP
31	Ca3pos39844991.1	3	39844991	9.40E−06	1, 2 and 4	NSP and NPP
32	Ca3pos39885397.1	3	39885397	1.26E−05	1, 2 and 4	NSP and NPP
33	Ca4pos19410981.1	4	19410981	8.00E−06	1, 2 and 4	NSP and NPP
34	Ca5pos3834038.1	5	3834038	7.49E−06	1, 2 and 4	NSP and NPP
35	Ca5pos46549190.1	5	46549190	8.46E−06	1, 2 and 4	NSP and NPP
36	Ca6pos24093514.1	6	24093514	2.61E−05	3 and 4	NSP and NPP
37	Ca6pos28350598.1	6	28350598	3.44E−05	3 and 4	NSP and NPP
38	Ca6pos53644742.1	6	53644742	3.20E−05	3 and 4	NSP and NPP
39	Ca7pos27650319.1	7	27650319	4.45E−05	3 and 4	NSP and NPP
40	Ca7pos47164247.1	7	47164247	1.35E−05	3 and 4	NSP and NPP
41	Ca8pos13311157.1	8	13311157	1.15E−05	3 and 4	NSP and NPP

SNP, single-nucleotide polymorphism; RFW, root fresh weight (g); RDW, root dry weight (g); SFW, stem fresh weight (g); SDW, stem dry weight (g).

### 3.6 Identification of putative candidate genes for associated SNPs

A BLAST search of significant SNPs identified from the current association panel was aligned against the CDC Frontier reference genome of chickpea, revealing the location of SNPs near the gene-rich region of the genome but present in intergenic regions. Irrespective of being in intron regions, the majority of the SNPs were near one or the other transcripts coding for some proteins or transcription factors. Many SNPs were located near the genes coding for general proteins well known for their metabolic function in growth and development like membrane proteins, DNA/RNA recognition/binding protein, ABC transporter, and protein kinase. SNP markers like 1_10074058 and 1_28905467 were linked to the genes governing the traits that are indirectly involved in root nodulation like auxin-responsive protein, environmental stress, and plant–microbe interaction candidate genes near the location of identified MTAs; their transcript IDs, proteins produced from them, and role of those proteins in plants based on previous reports are given in **Tables 5A** and **Table 5B**.

**Table 5A:** Putative candidate genes identified for NON at the 10-kb region of linked SNPs along with their molecular functions.

SNP	Start	End	Sequence description	Biological function
1_10074058	10071267	10077948	Auxin-responsive protein IAA26-like	Regulation of transcription, auxin-activated signaling pathway, main hormone for root nodule initiation
1_19310421	19307421	19312421	SNF1-related protein kinase regulatory subunit beta-3	Regulation of protein kinase activity, cellular response to nitrogen levels, response to sucrose
2_825900	805900	845900	Transmembrane protein, putative	
2_12931949	12913554	12916946	3392	Emp24/gp25L/p24 family protein
	12920791	12923570	2779	Hypothetical protein VIGAN_08277400
4_2035604	2031504	2036704	Adenosine kinase 2	Purine ribonucleoside salvage, AMP biosynthetic process, phosphorylation
6_16019517	16042593	16042985	Thermospermine synthase ACAULIS5-like	
6_16019517	16062478	16063311	RuBisCO-associated protein-like	Carbohydrate metabolic process
6_16019517	16075075	16075869	Probable UDP-arabinopyranose mutase 5	Plant-type cell wall organization or biogenesis

**Table 5B:** Putative candidate genes identified for NFW at the 10-kb region of linked SNPs along with their molecular functions.

SNP	Protein	Function	References
**1_29852482**	FT-interacting protein 7-like	Regulators of plant immunity	Ashraf et al., 2018
	FT-interacting protein 7-like	Plant immunity	
**1_28905467**	BZIP transcription factor TGA10-like isoform X2	Environmental stress	Li et al., 2020
	FT-interacting protein 7-like	Vernalization response	Gretsova et al., 2023
**6_3345569**	60S ribosomal protein L34	Alternate spicing	Denise et al., 2019
	PsbP-like protein 1, chloroplast isoform X2	Assembly of the PSII SCs is required for adaptation to changing light intensity	Che et al., 2020
**8_11345025**	50S ribosomal protein L23, chloroplast	Support translation under stress	
**8_11435804**	F-box protein At2g39490-like	Disease resistance	Zhou et al., 2013
**8_12988095**	Transcription initiation factor TFIID subunit 6	Plant–microbe interactions	Ning et al., 2021

NFW, nodule fresh weight (g); SNPs, single-nucleotide polymorphisms.

## 4 Discussions

### 4.1 Genome-wide association analysis as a competitive tool for identification of genomic regions controlling chickpea nodulation traits

The chickpea crop has a narrow genetic base (Kumar et al., 2017; Singh et al., 2022). Due to the use of a limited number of accessions/donor parents in breeding programs, the sensitivity of chickpea productivity toward biotic and abiotic stress is increased (Muehlbauer and Sarker, 2017). Developing high-nodulation genotypes will help in the augmentation of chickpea potentiality. In this investigation, on the basis of frequency distribution, it may be concluded that a wide range of variability existed for NON, NFW, RFW, RDW, and other yield-contributing traits. Further, correlation coefficients of our association panel revealed that nodule fresh weight and nodule dry weight were positively and significantly correlated with yield plant^−1^ at genotypic and phenotypic levels. These results are in accordance with those of several studies (Chandana et al., 2023; Elias, 2009; Mohamed and Hassan, 2015). This indicated that the development of effective and promising nodules helps in increasing yield. This was probably due to the uptake of atmospheric nitrogen through the process of biological nitrogen fixation. To accelerate the breeding programs, conventional breeding efforts need to be augmented using genomics-assisted breeding (Varshney et al., 2007, 2013). In our study, we carried out resequencing by whole-genome resequencing, which resulted in the identification of 1,198,121 SNPs. The sequencing cost was curtailed as an advance in the next-generation sequencing technology (Varshney et al., 2009), and the availability of chickpea draft genome (Jain et al., 2013; Varshney et al., 2013; Gupta et al., 2017) led to the identification of several millions of SNPs and other molecular markers. Further integrating whole-genome resequencing information with precise phenotyping provides detailed information about all genetic variants and enables the discovery of novel variations.

### 4.2 Diversity and linkage disequilibrium analysis for identifying MTAs close to the trait of interest

In GWAS analysis, population structure may be a confounding factor that must be addressed to avoid false associations. Population structure and uneven relatedness among the individuals of the population act as confounding factors and lead to spurious identification of MTAs (Zhang et al., 2010). Admixture of PCA and phylogenetic tree using the neighbor-joining method are popular approaches for accurately inferring population structure derived from the genome-wide association panels using high-density SNPs (Abraham and Inouye, 2014). The use of genotypes from various origins of the world in the study may be the reason for the three clear-cut subpopulations in the GWAS panel (Upadhyaya et al., 2001). In neighbor-joining dendrograms, the different clustering patterns are clearly visible, and similar results were reported by Thudi et al. (2021). The use of a diverse panel of genotypes can provide more valuable inference compared to bi-parental populations (Vos-Fels et al., 2017) by taking advantage of maximum allelic diversity and historic recombination events; the present study materials having diverse lines with their distant parentage ensure the required diversity for the association study. The genetic diversity of chickpea germplasm could provide important information for selecting effective parental breeding strategies as well as a better understanding of natural variations in phenotypic traits and their genetic background (Raina et al., 2019). The genome-wide LD decay estimated for the present investigation is on par with the previous association studies of chickpea for complex yield traits (Upadhyaya et al., 2016; Kujur et al., 2015). An LD value of 373 kb illustrates the string association of SNP markers with the trait of interest. The SNP density analysis revealed that SNPs are distributed across all the chromosomes, and this helps to identify all possible causal variants for traits of interest. This aligns with a previous study on Desi and Kabuli chickpea genotypes and gives the importance of geographic origin and adaptive environments in genotype clustering (Basu et al., 2018; Roorkiwal et al., 2022). The major challenge for GWAS is to control the false positives primarily caused by population structure and family relatedness (Kaler et al., 2020). To avoid such bias, a stringent selection procedure of the Bonferroni correction was applied; a total of 643, 720, and 439 SNPs were identified in the association panel through the BLINK model, FarmCPU model, and MLM, respectively. Identification of such a huge number of SNPs can provide deeper insights into the genomic regions associated with traits of interest. In this study, our main target was nodulation traits such as NON, NFW, and other associated traits like shoot and root because the association between shoot- and root-related traits indirectly contributes to the nodulation. Hence, the identification of genomic regions for root and shoot regions will also help in the simultaneous improvement of the legume crop.

### 4.3 MTAs controlling nodulation and yield contributing traits

In the marker and nodulation trait association, we obtained 12 SNPs in the MLMM for NON-above FDR or −log10 p-value of 7, which were highly significant markers for the number of nodules and SNPs Ca4pos29278891.1 and Ca4pos34659769.1, identified through FarmCPU for location 1 and had phenotypic effects of 3.27% and 4.08%, respectively. As of now, there are no reported large-scale studies on the identification of SNPs for the number of nodules in chickpea; these SNPs will serve as markers for molecular studies and marker-assisted breeding in chickpea. For NON, we found highly significant MTAs on chromosome numbers 2 (three SNPs), 3 (one SNP), 4 (four SNPs), and 7 (two SNPs) through the MLMM. These findings indicate that an improved understanding of these markers and genomic regions of chickpea is necessary to improve the benefits of rhizobial symbiosis in chickpea root nodulation. The trait NFW, an important nodulation trait, was used to measure the nodulation potential of legumes, especially in the case of chickpeas, as they produce indeterminate nodules and also have a direct association with chickpea growth attributes and biological nitrogen fixation (Hazra et al., 2021). Istanbuli et al. (2024) reported for NFW in their study, the maximum number of SNPs on chromosome number 4 in genomic regions was between 52- and 58-Mb regions. However, in our study, we report 75 novel markers for the nodulation traits. SNP Ca5pos20514758.1 reported for NFW and presented on chromosome number 5 had 42.28% of PVE. The SNP was also based on gene annotation in the NCBI of the Cicer genome and was found to be an associated gene that encodes for important proteins like auxin-induced proteins, which are involved in nod factor binding export proteins in *Medicago* (Gully et al., 2018). Plant height in chickpeas is influenced by 200 SNPs; additionally, it is consistent with earlier studies showing multiple loci controlling plant height in different crops. The multi-locus control of plant height is influenced by both genetic and environmental factors, although specific details are not fully understood (Yang et al., 2021). As provided in **Supplementary Tables 2**-**11**, traits such as DTM, DTF, RFW, RDW, SFW, SDW, NPP, NSP, and yield were controlled by more than at least 130 SNP markers. Among them for the trait DTF, the significant SNPs were Ca4pos99711.1 with 2.8% of PVE and Ca5pos30670011.1 with 9.15% of PVE identified for location 1. SNP Ca7pos16225558, which is identified in both the FarmCPU model MLM, had 5.94% of PVE. SNP Ca6pos47821883.1 identified for location 3 had 48.43% of PVE.

Through GWAS, we were able to identify highly significant markers for NFW through different models as mentioned in the Results section. Therefore, these markers serve indirectly in the improvement of nodulation in chickpea, which supports the establishment and survival of plants in adverse conditions, thereby promoting plant productivity (Gupta et al., 2015); also, our analyses provide new insights into the identities of markers and phenotypic influences on identified markers by providing causal variants for responsible markers that explained phenotypic variance. The MTAs identified in more than one environment were grouped as stable/promising ones, while MTAs with greater than 15% phenotypic variation were grouped as major MTAs. The major MTAs found in our study identify a large number of stable SNPs. Thus, the GWAS results in the identification of stable markers for all the traits such as four SNPs for RFW, seven SNPs for SFW, and 17 stable SNPs for SDW, which may be regarded as nodulation-related traits, as these traits play a role in root development and establishment. The precise phenotyping and high accuracy may lead to a greater number of stable expressions along the different locations/conditions presumed to be the real association of these markers for governing the phenotypic expression of the studied traits. Limited stable SNPs for root traits through GWAS have also been reported earlier (Thudi et al., 2014). Pleiotropy occurs when a single genetic variant is associated with multiple phenotypic outcomes. Hence, it is common to find markers to be associated with more than one trait, i.e., pleiotropic influence. Significant pleiotropic loci were detected for nodulation and yield. Thus, we can improve both traits and the productivity of the crop simultaneously and indirectly. Similarly, we also found different stable MTAs showing pleiotropic effects between different yield and nodulation traits due to their interdependence on each other. These traits are bound to share genes in common, and the results are supported by the presence of a significant positive correlation between nodulation and yield traits in our study. The PVE% explained by SNPs refers to the proportion of the total variability observed in a specific trait or phenotype that can be attributed to genetic variations in SNPs. PVE% is a measure of the contribution of genetic factors to a particular trait. The SNPs with high PVE can be considered as major and significant for that particular trait. Two SNPs were found to be stable at locations 1, 3, and 4; seven SNPs were found to be stable at locations 1 and 3 for the trait NON. SNP 2_825902, which was found on chromosome number 2, had 27.33% of the PVE. Also, for the other traits, we identified many good SNPs whose number and percentage of PVE are already explained in the Results section. Stable SNPs with high PVE can be used as fixed effects in the genomic selection pipeline.

### 4.4 Gene enrichment analysis for nodulation traits

In the current investigation, as our main focus was on nodulation traits, we tried to provide genes that are located within the identified MTAs for nodulation traits, and the investigation revealed MTAs within a prominent genomic region housing candidate genes responsible for governing diverse functions in plant growth, developmental processes, biotic and abiotic pathways, stress tolerance, and intricate nodulation pathways. Particularly noteworthy is the stably identified MTA 1_10074058 associated with the trait of interest NON. This locus encodes an auxin-responsive protein IAA26-like, a pivotal hormone instrumental in root initiation and initiation of root nodules. Furthermore, its involvement in transcriptional regulation and auxin-activated signaling pathways underscores its significance (Luo et al., 2018). Similarly, the SNP 1_19310421 associated with the NON trait is situated in a genomic region where the gene codes for an SNF1-related protein kinase regulatory subunit beta-3. This subunit is implicated in the regulation of protein kinase activity, cellular responses to nitrogen levels, and the intricate response to sucrose signaling (Jamsheer et al., 2021). These findings collectively contribute to our understanding of the genetic underpinnings of the observed traits and the multifaceted processes governing plant–nutrient interactions and developmental responses.

## 5 Conclusion

Biological nitrogen fixation (BNF) stands for a sustainable and globally applicable avenue for supplying nitrogen to agricultural systems. An effective strategy to augment BNF involves the breeding and utilization of legume varieties possessing enhanced BNF capacity. Notably, our results demonstrated the effectiveness of phenotyping with increased row-to-plant spacing in elucidating traits related to nodule formation. Leveraging association studies, we successfully identified noteworthy and stable MTAs linked to the traits of interest. A total of more than 800 MTAS have been reported for root, shoot, yield, and other morphological traits. These MTAs can be compared in future studies to identify the most probable location of causal variation for their respective traits. Subsequent *in silico* analysis unveiled that a substantial proportion of these MTAs were situated within intergenic regions, with the potential to modulate genes associated with the focal traits. The seven novel SNPs, namely, Ca2pos2169123.1, Ca2pos28892511.1, Ca3pos36960027.1, Ca4pos43172963.1, Ca5pos2026241.1, Ca5pos21727560.1, and Ca7pos47087116.1, identified for the number of nodules were highly significant and found common in different models of GWAS were considered as SNPs probably controlling the genomic regions for the trait NON, as these MTAs are present near the genes that aid in biological nitrogen fixation pathway. SNP Ca5pos20514758.1 identified for NFW in locations 1 and 3 was found with a PVE of 42.28% and could be used to construct the cleaved amplified polymorphic sequence (CAPS) or Kompetitive allele specific PCR (KASP) markers that can serve as a PCR-based marker for identifying polymorphism for nodulation traits in chickpea and other legumes. The stable SNPs characterized by a high proportion of PVE will be integrated as fixed effects within the genomic selection pipeline, accentuating their potential impact on future breeding efforts. The identified candidate genes could be exploited in marker-assisted breeding, genomic selection, and genetic engineering to improve the nodulation efficiency in legumes and other crop species.

## Data Availability

The datasets presented in this study can be found in online repositories. The names of the repository/repositories and accession number(s) can be found in the article/**Supplementary Material**.
