# Supplementary material for: Genome-wide association mapping identifies novel SNPs for root nodulation and agronomic traits in chickpea
Source: Front Plant Sci. 2024 Oct 15;15:1395938. doi: 10.3389/fpls.2024.1395938 (PMC11518797; doi:10.3389/fpls.2024.1395938)
Supplement: Supplementary file 1 [file DataSheet1.zip › Supplementary figures 6-10.pdf]

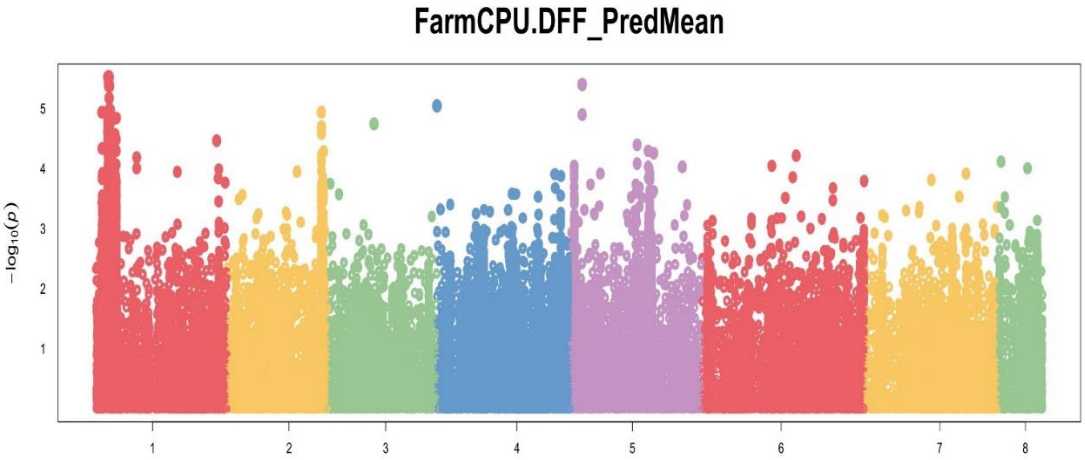

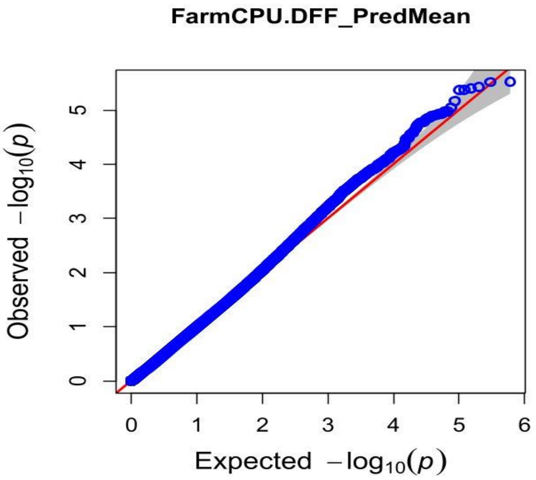

Supplementary Figure6a: Manhattan plot illustrating SNPs linked to Days fifty percentage flowering along with their corresponding statistical significance represented by Q-Q plot.

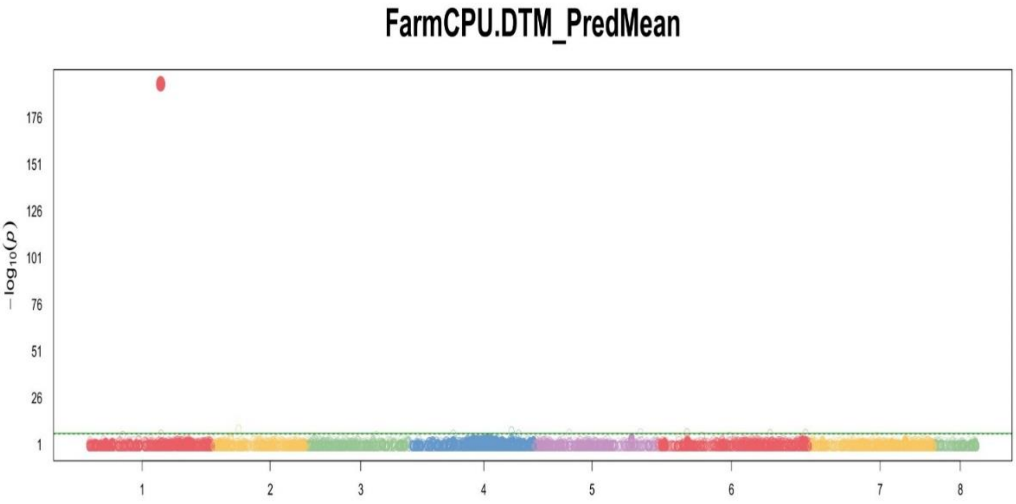

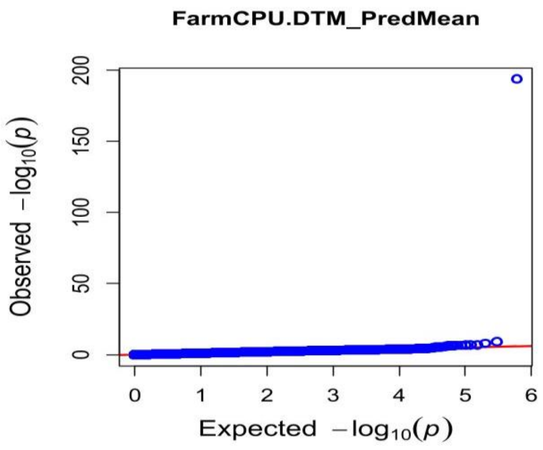

Ssupplementary figure6b: Manhattan plot illustrating SNPs linked to Days to maturity along with their corresponding statistical significance represented by Q-Q plot.

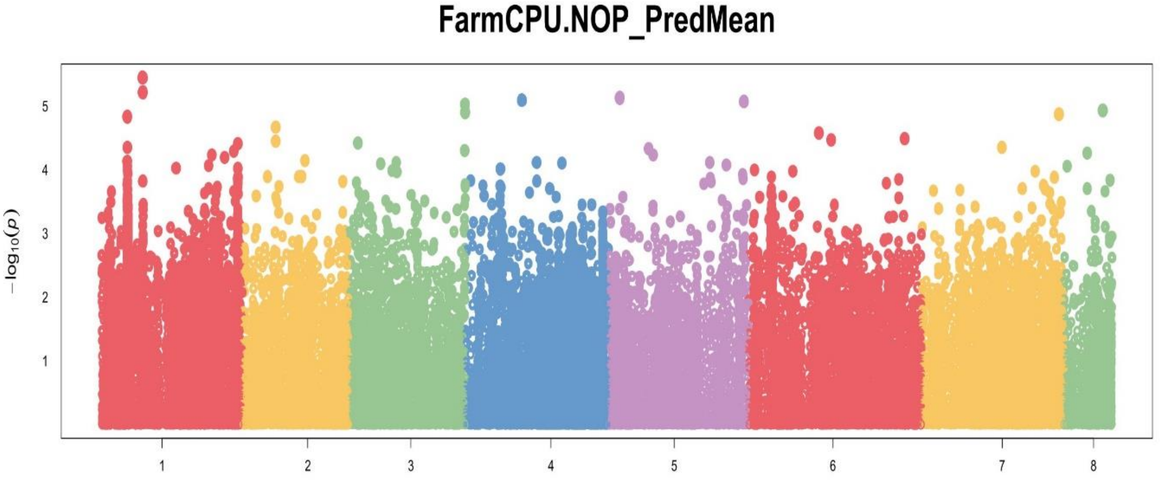

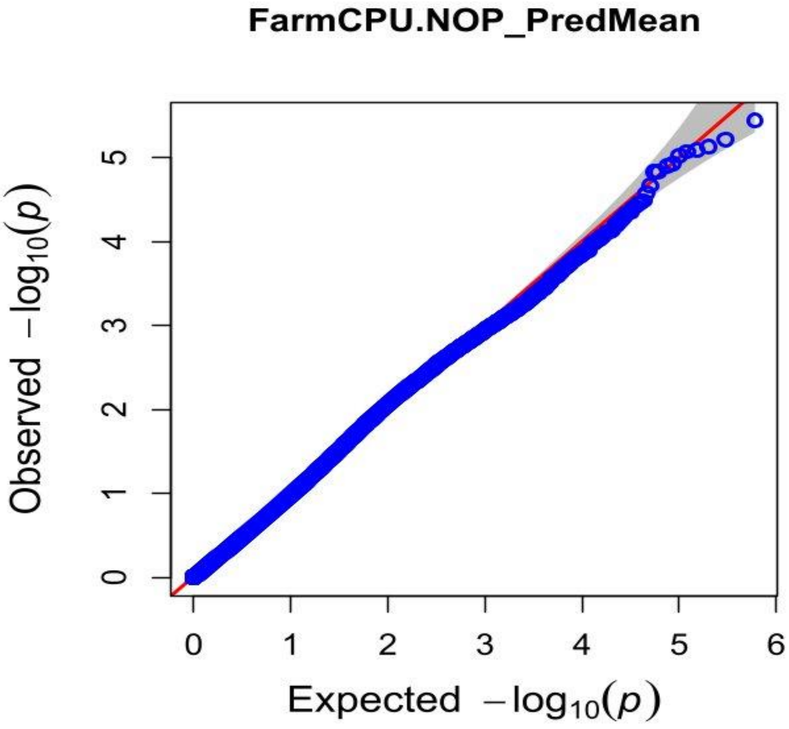

Supplementary Figure6c: Manhattan plot illustrating SNPs linked to number of pods along with their corresponding statistical significance represented by Q-Q plot.

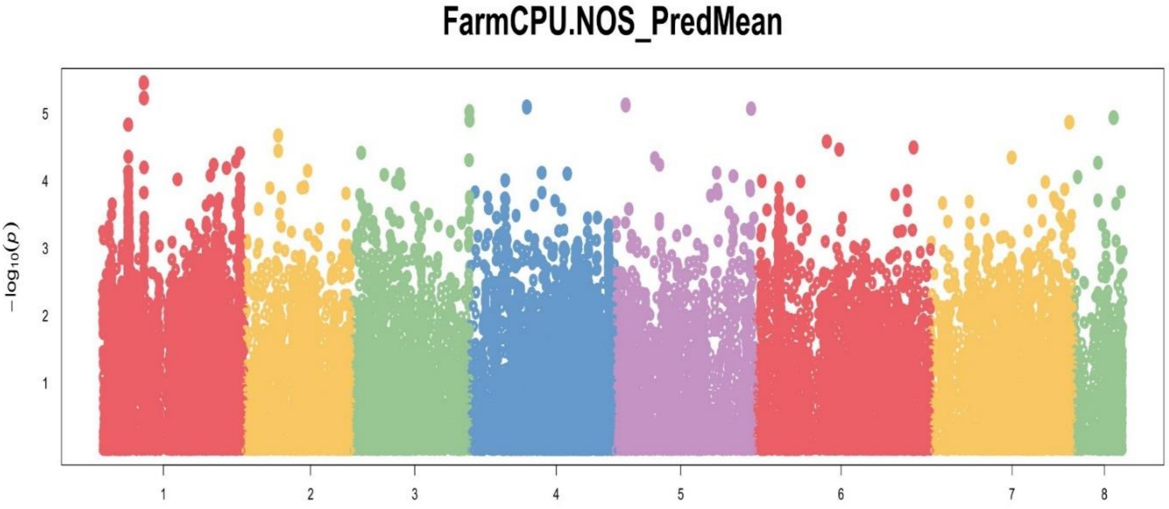

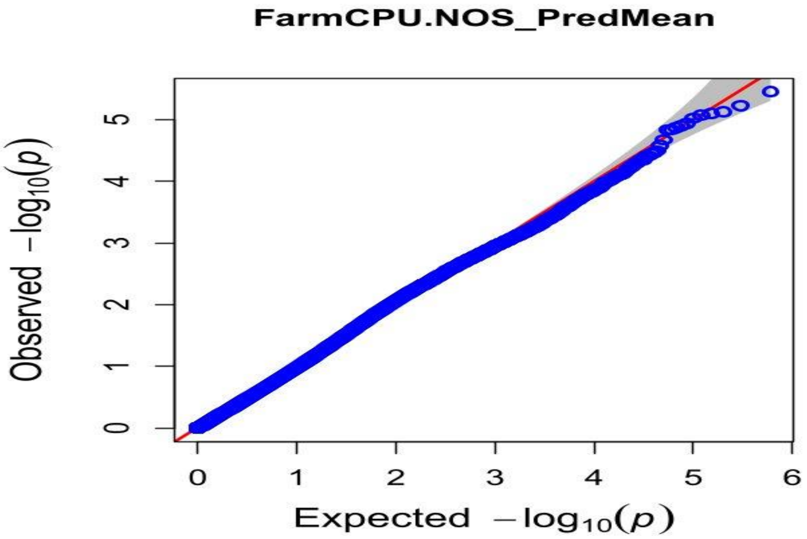

Supplementary Figure6d: Manhattan plot illustrating SNPs linked to number of seeds along with their corresponding statistical significance represented by Q-Q plot

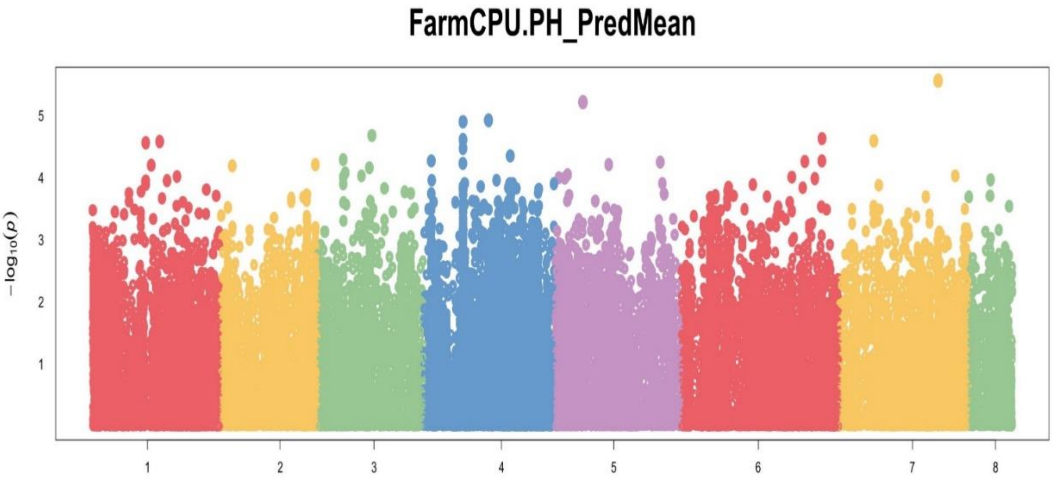

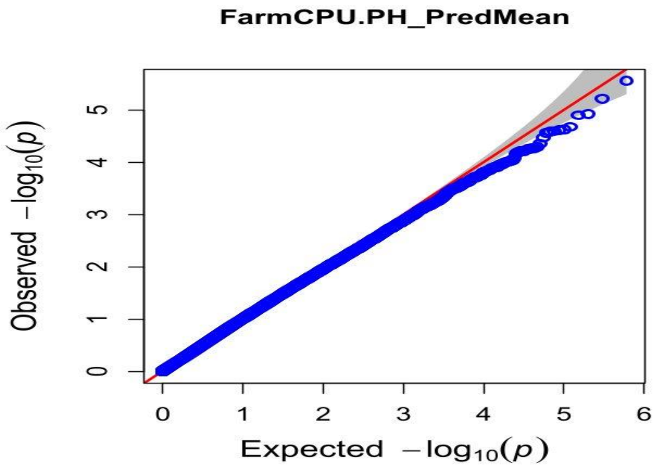

Supplementary Figure6e: Manhattan plot illustrating SNPs linked to plant height along with their corresponding statistical significance represented by Q-Q plot

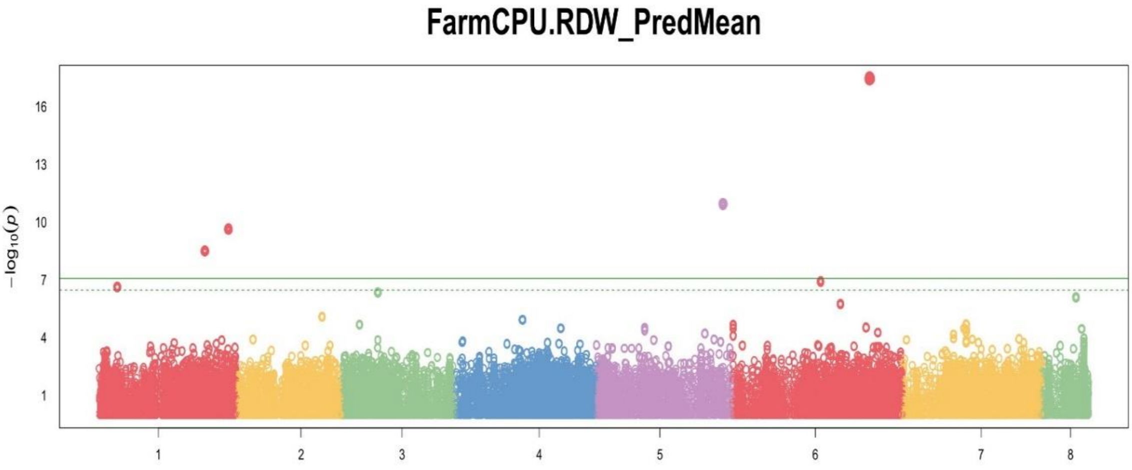

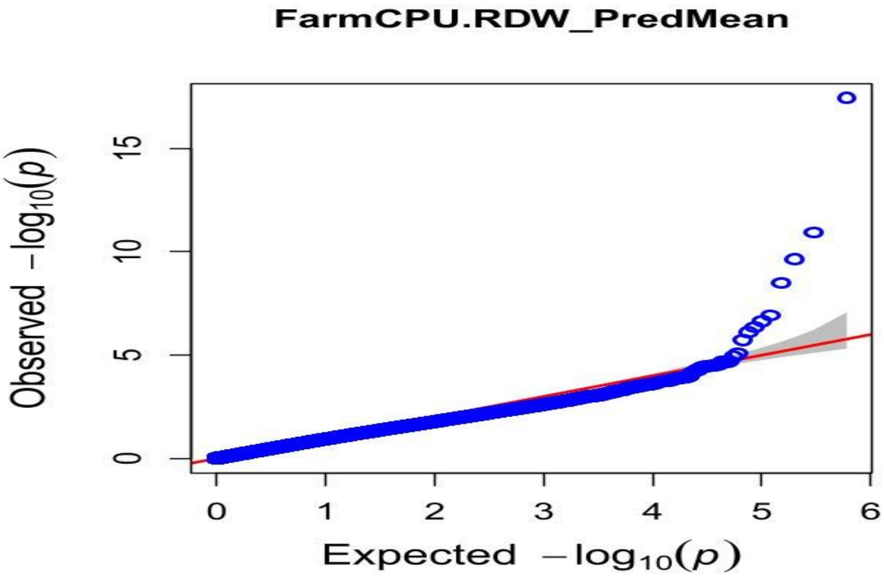

Supplementary Figure6f: Manhattan plot illustrating SNPs linked to root dry weight with their corresponding statistical significance represented by Q-Q plot

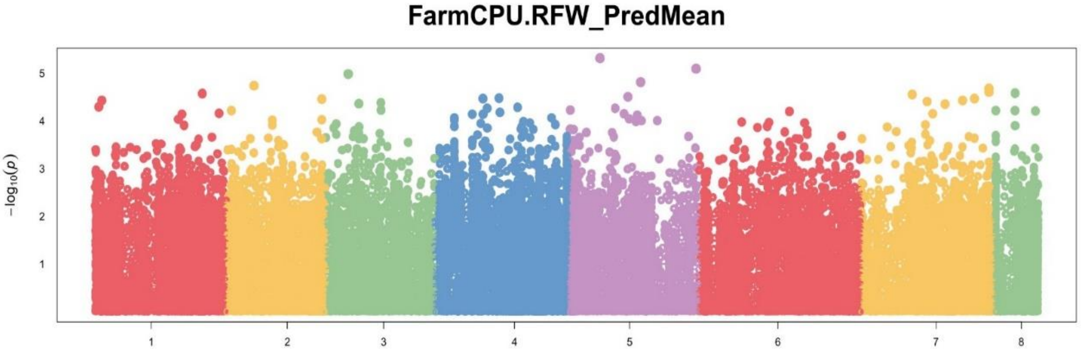

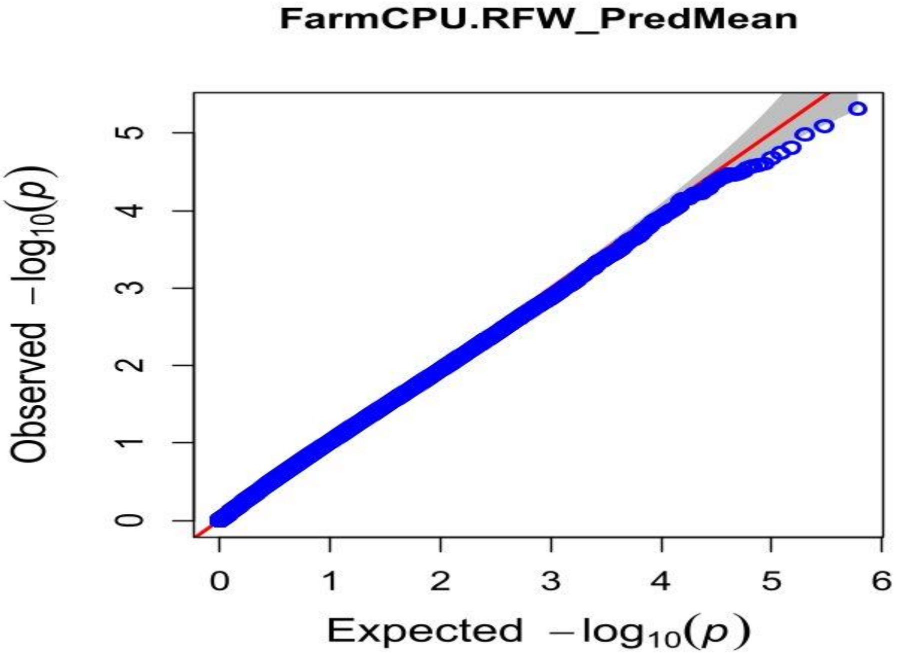

Supplementary Figure6g: Manhattan plot illustrating SNPs linked to root fresh weight with their corresponding statistical significance represented by Q-Q plot

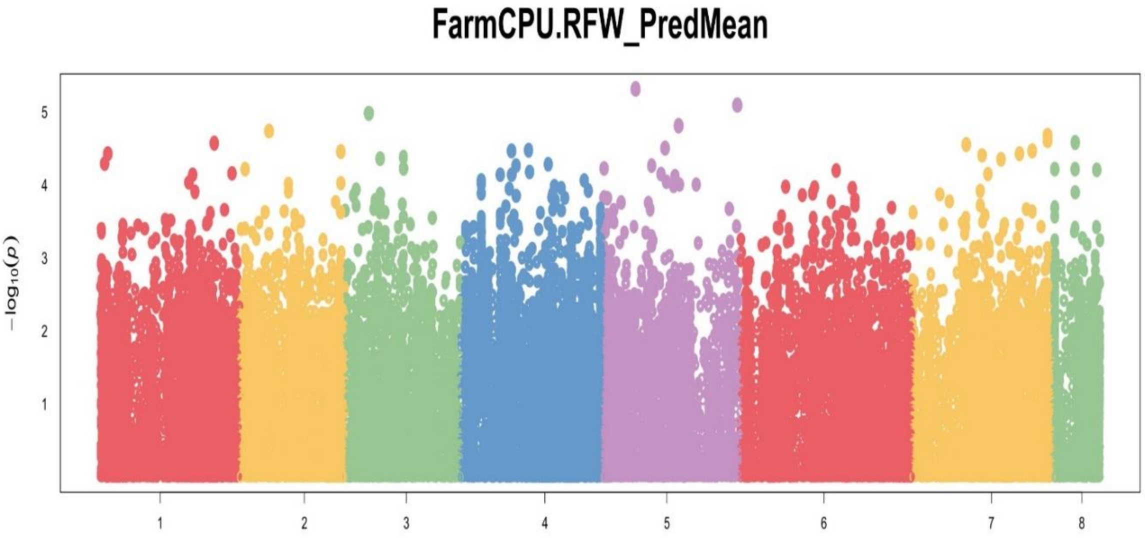

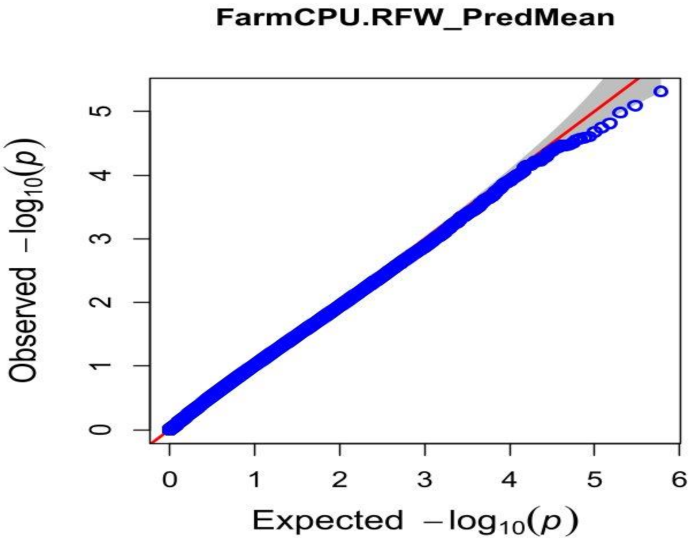

Supplementary Figure6g: Manhattan plot illustrating SNPs linked to root fresh weight with their corresponding statistical significance represented by Q-Q plot

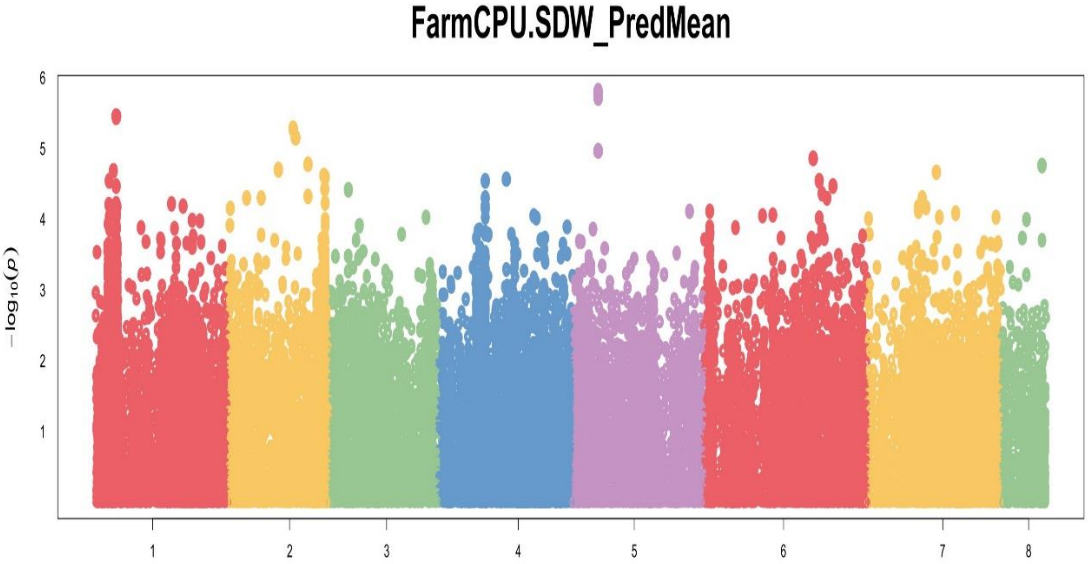

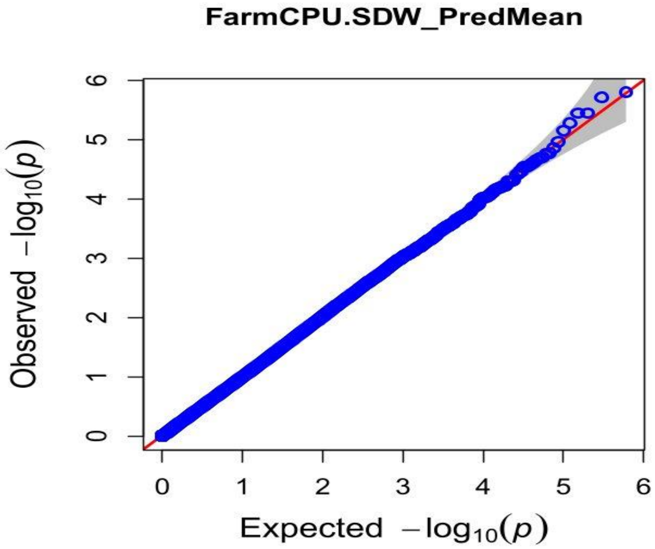

Supplementary Figure6h: Manhattan plot illustrating SNPs linked to shoot dry weight with their corresponding statistical significance represented by Q-Q plot

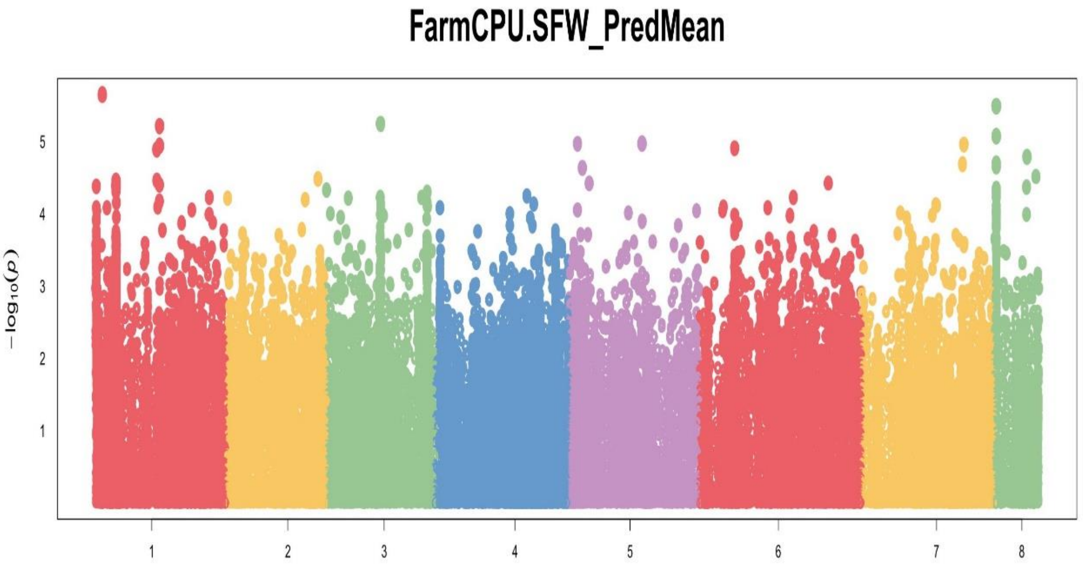

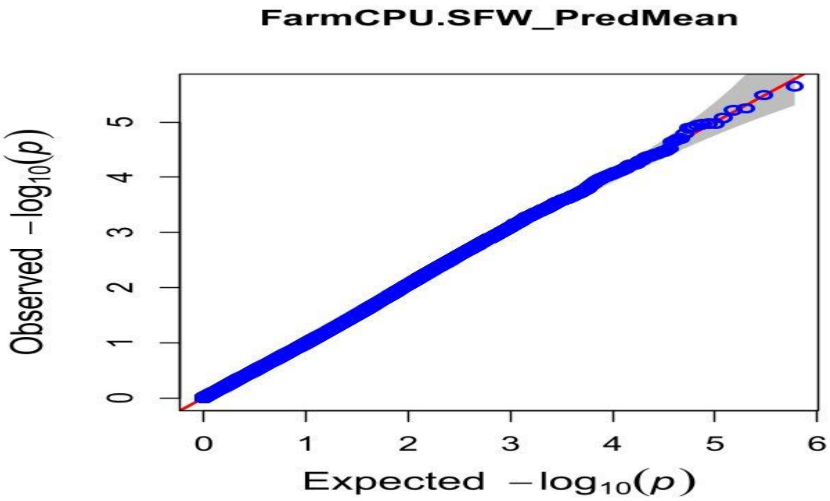

Supplementary Figure6i: Manhattan plot illustrating SNPs linked to shoot fresh weight with their corresponding statistical significance represented by Q-Q plot

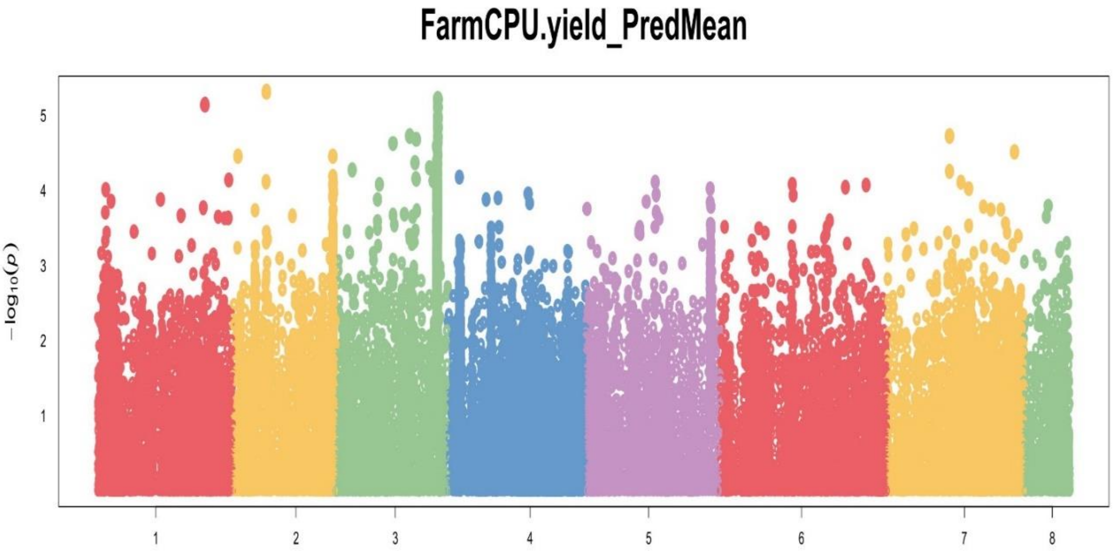

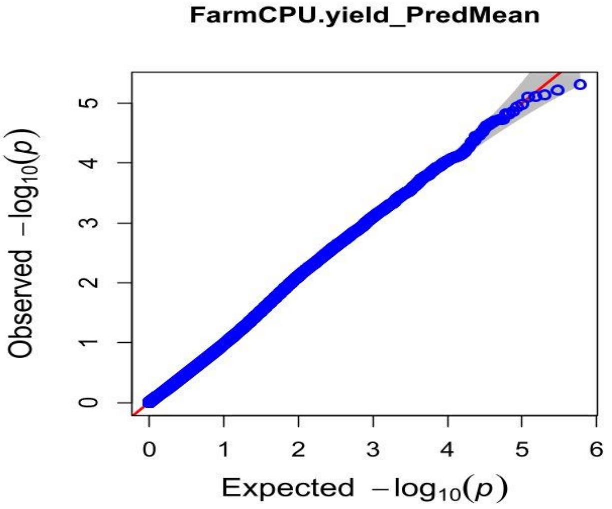

Supplementary Figure6j: Manhattan plot illustrating SNPs linked to yield with their corresponding statistical significance represented by Q-Q plot

Supplementary Figure6:Manhattan plot illustrating SNPs linked to specific chickpea traits, along with their corresponding statistical significance represented by Q-Q plot for location 2 identified from FarmCPU model

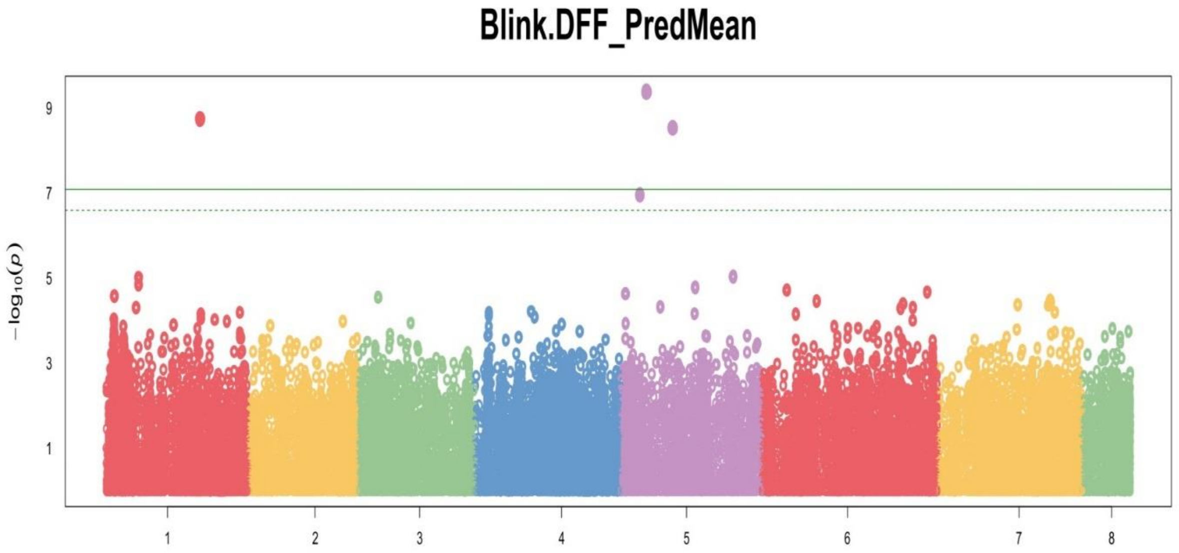

.

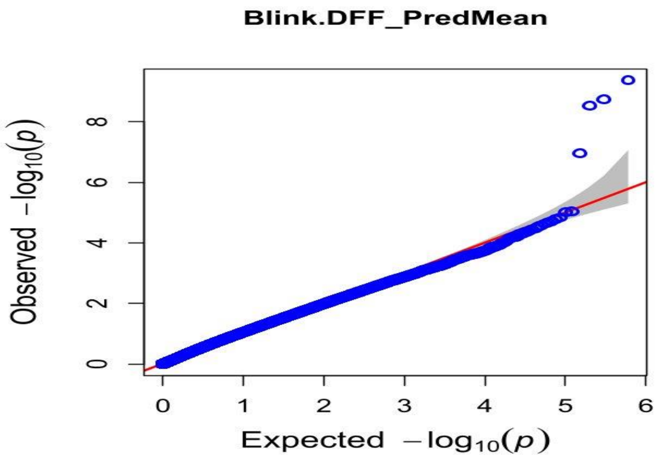

Supplementary Figure7a: Manhattan plot illustrating SNPs linked to Days fifty percentage flowering along with their corresponding statistical significance represented by Q-Q plot.

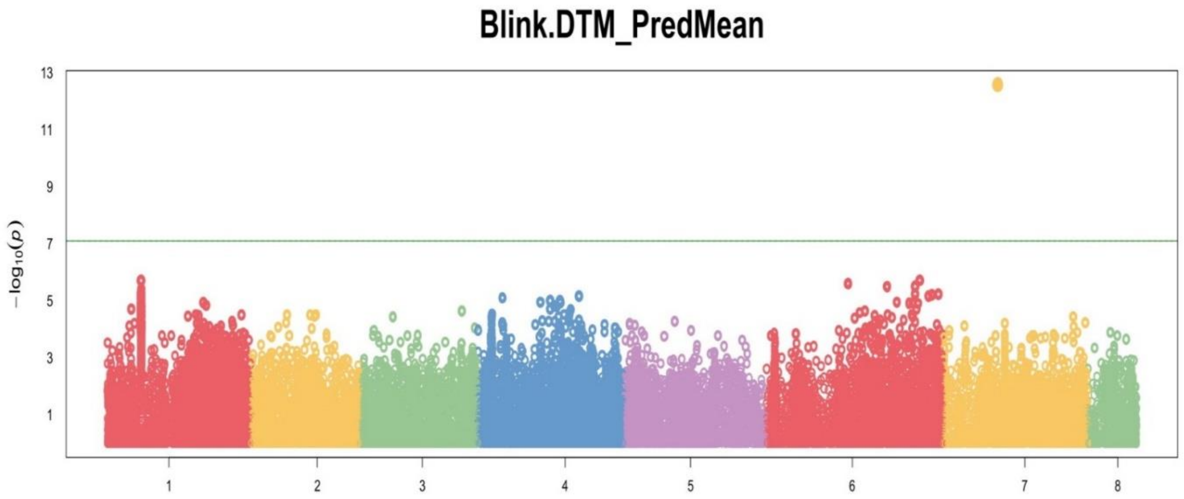

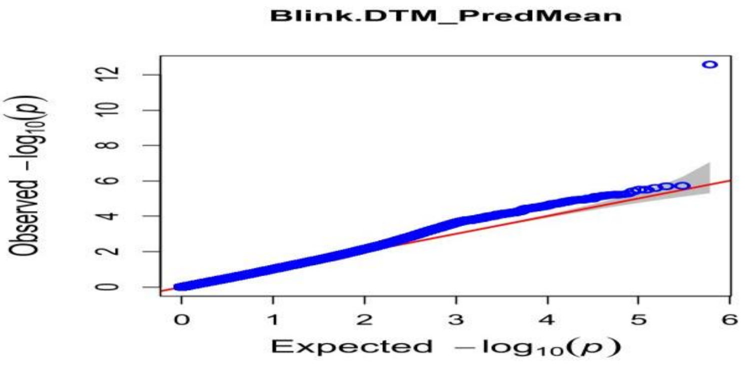

Supplementary Figure7b: Manhattan plot illustrating SNPs linked to Days to maturity along with their corresponding statistical significance represented by Q-Q plot

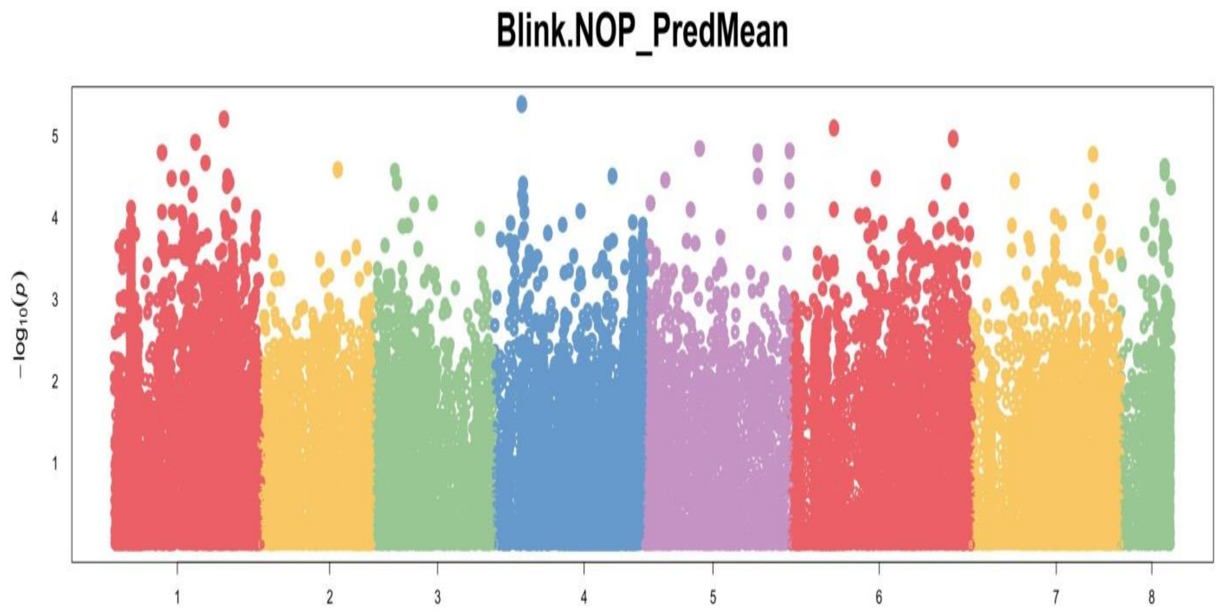

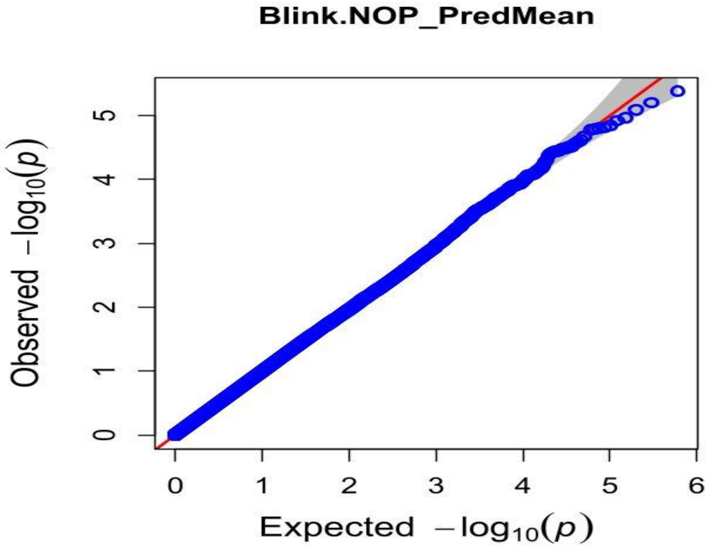

Supplementary Figure7c: Manhattan plot illustrating SNPs linked to number of pods along with their corresponding statistical significance represented by Q-Q plot.

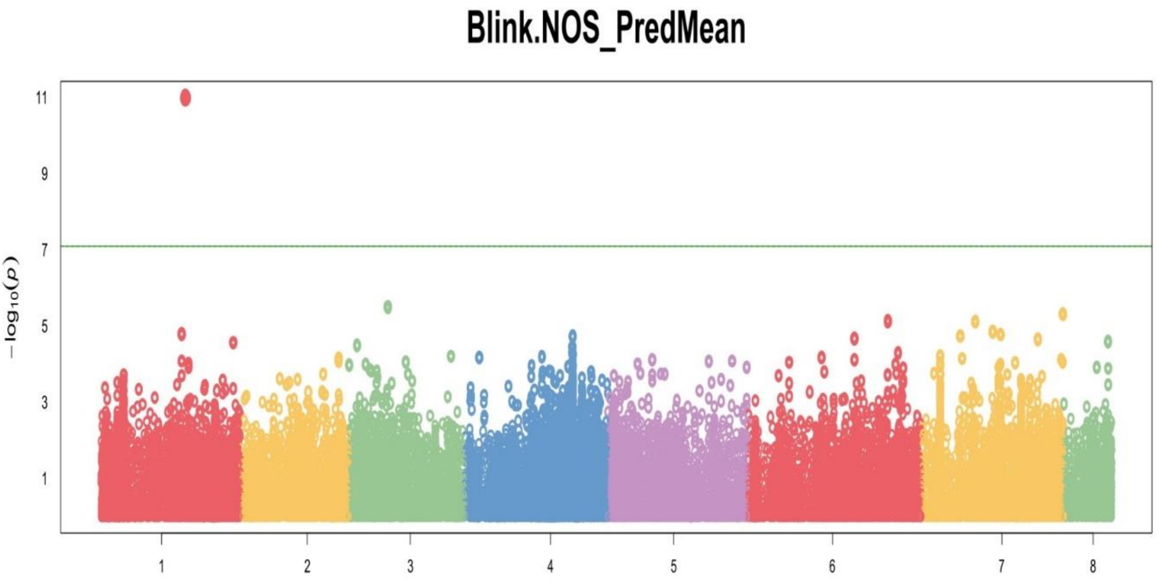

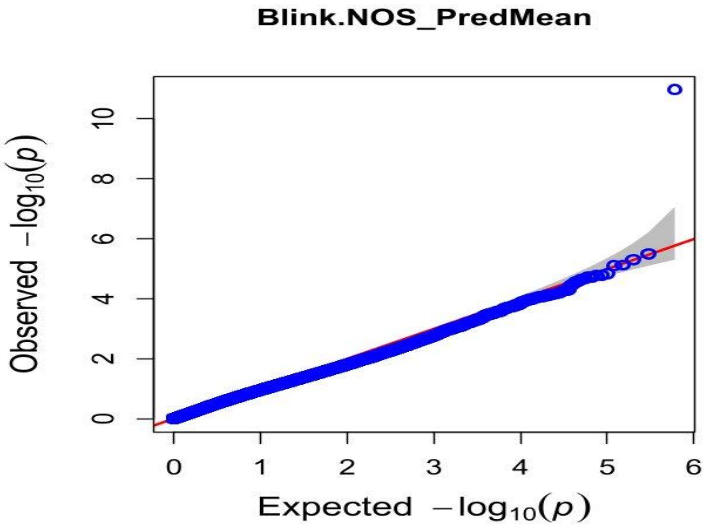

Supplementary Figure7d: Manhattan plot illustrating SNPs linked to number of seeds along with their corresponding statistical significance represented by Q-Q plot.

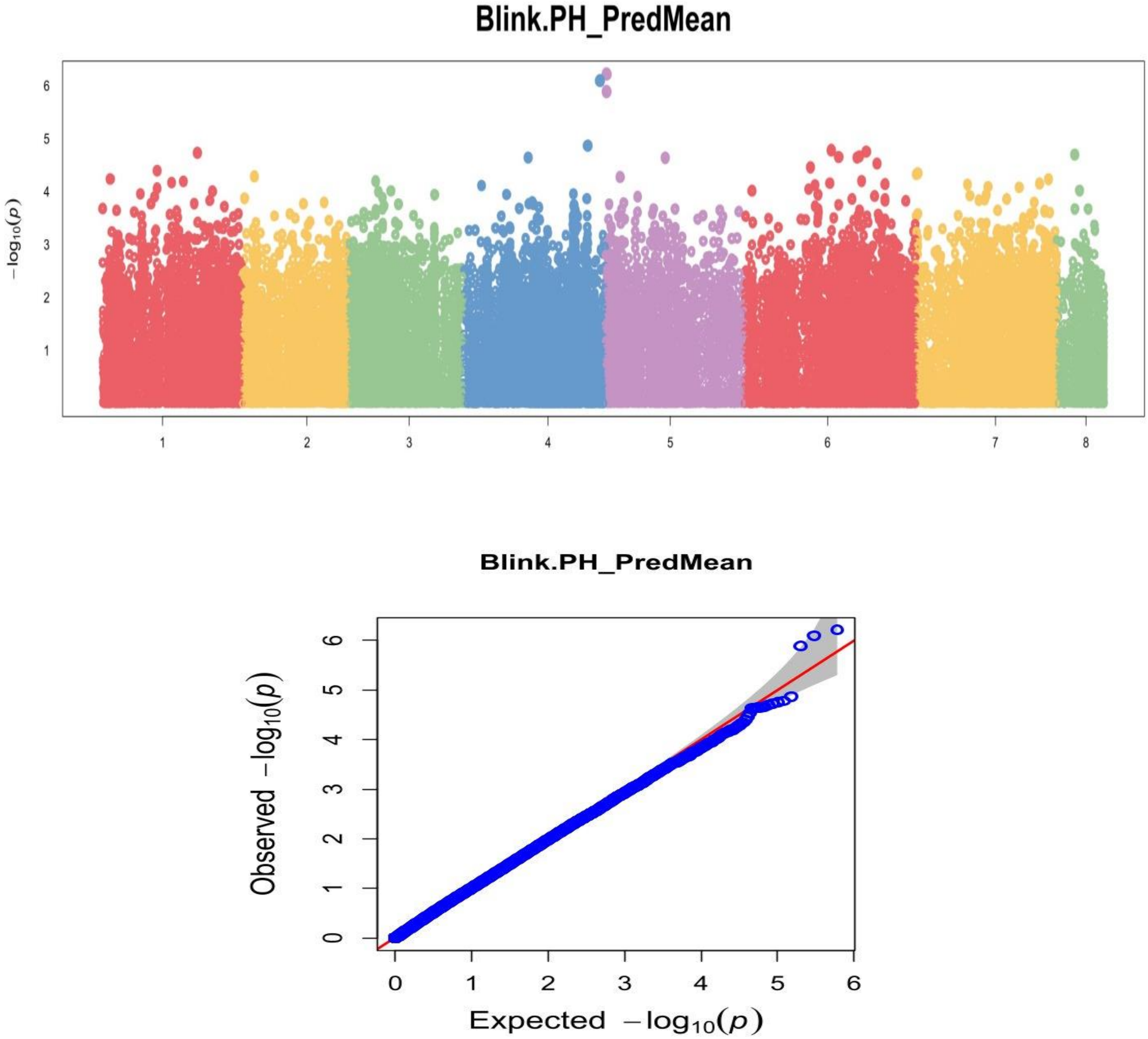

Supplementary Figure7e: Manhattan plot illustrating SNPs linked to plant height along with their corresponding statistical significance represented by Q-Q plot

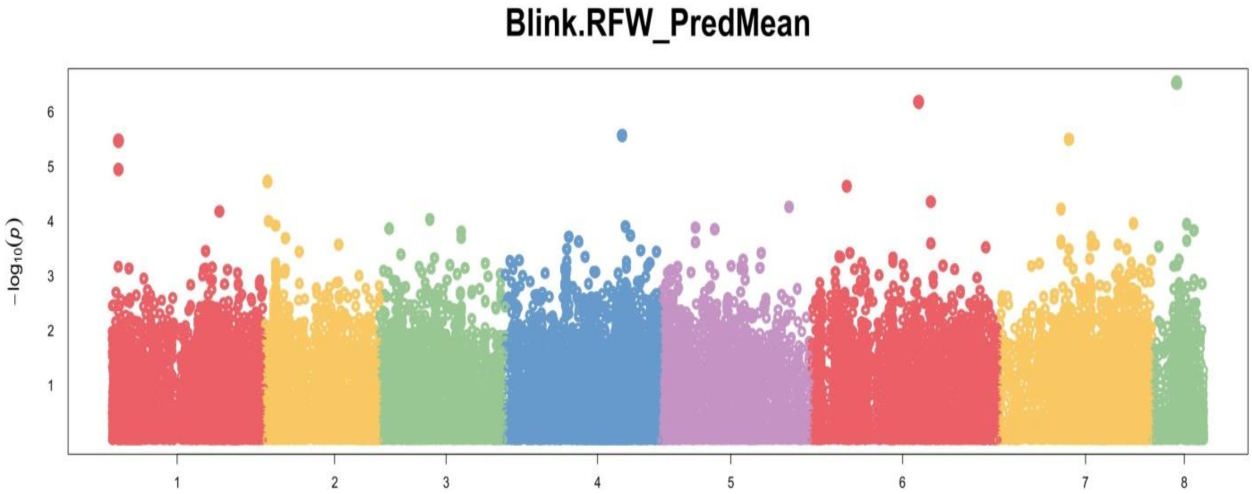

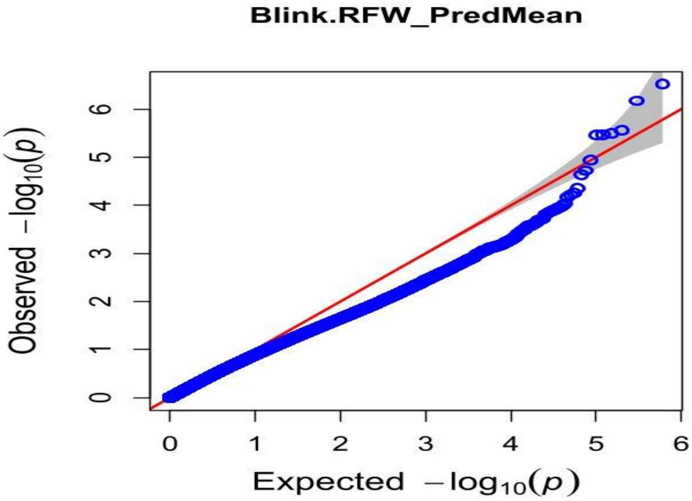

Supplementary Figure7f:Manhattan plot illustrating SNPs linked to root dry weight with their corresponding statistical significance represented by Q-Q plot

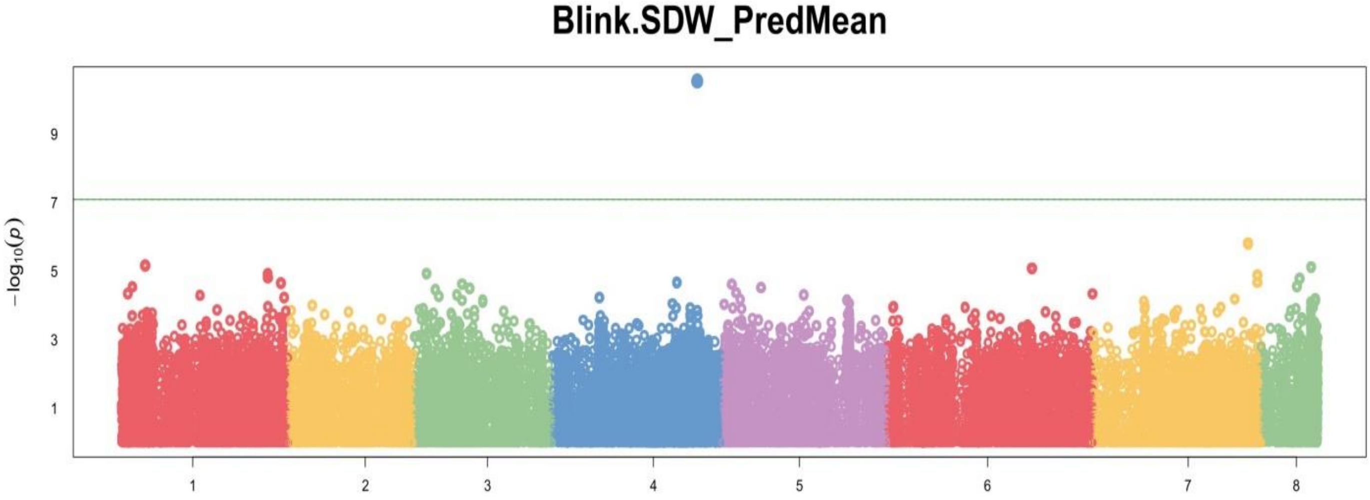

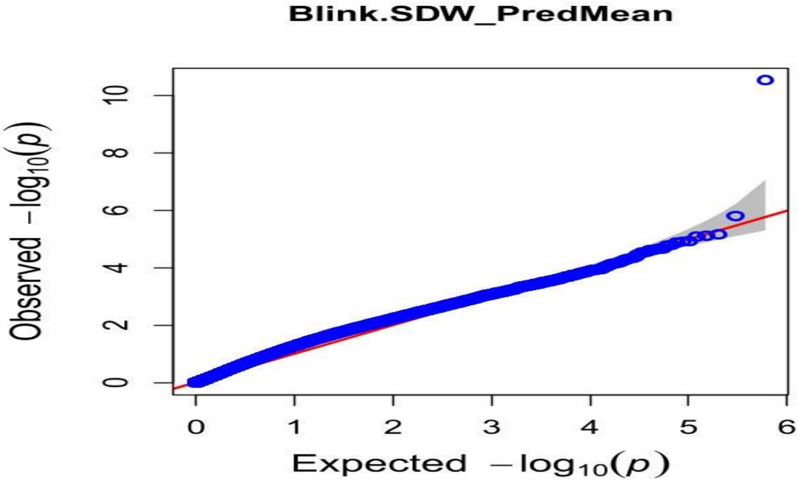

Supplementary Figure7h: Manhattan plot illustrating SNPs linked to r shoot dry weight with their corresponding statistical significance represented by Q-Q plot

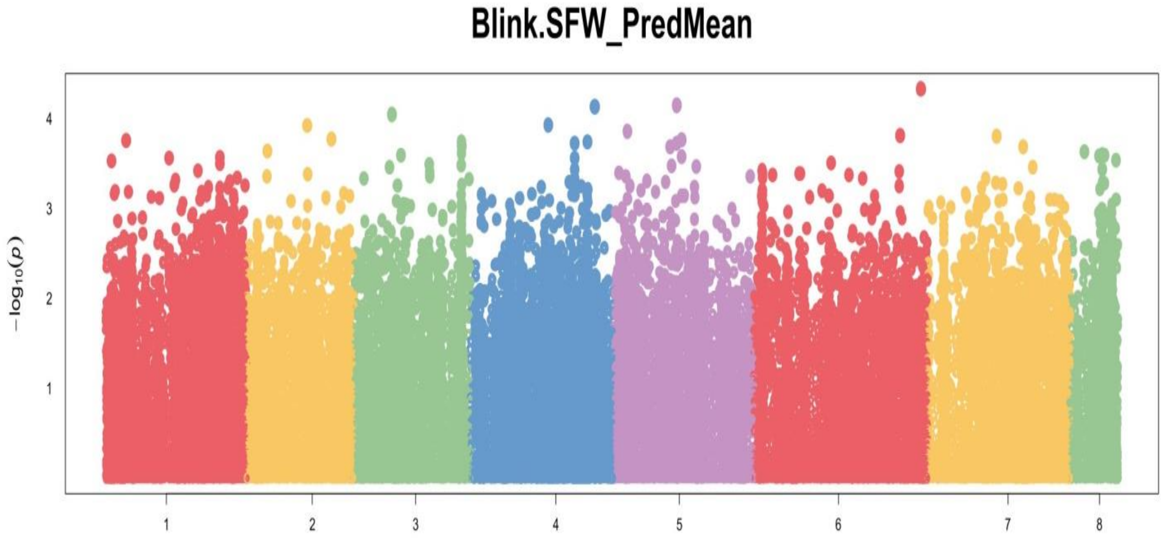

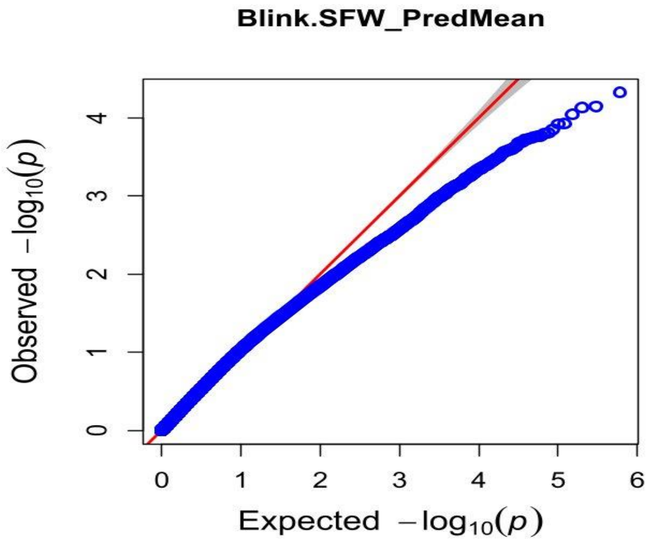

Supplementary Figure7i:Manhattan plot illustrating SNPs linked to shoot fresh weight with their corresponding statistical significance represented by Q-Q plot

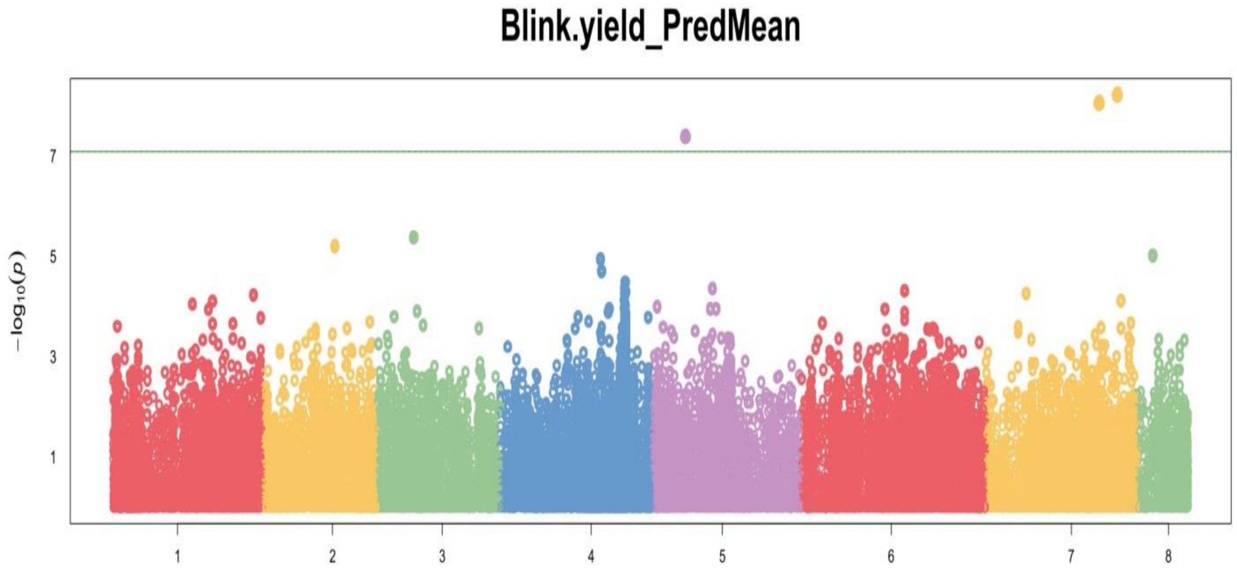

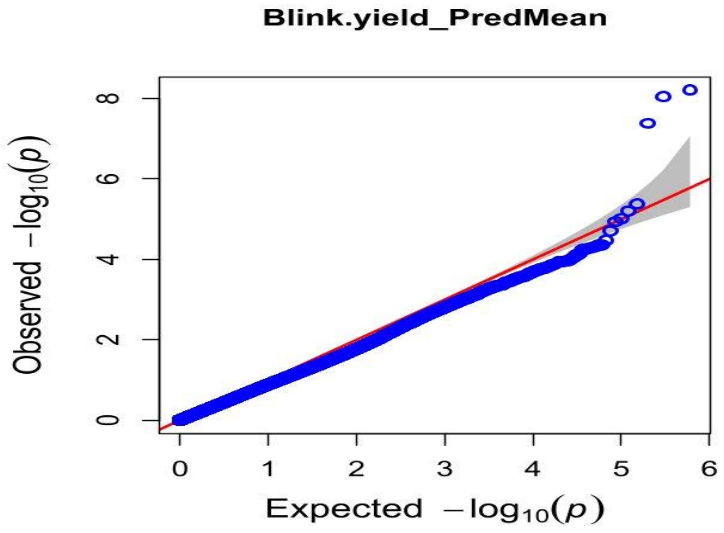

Supplementary Figure7j: Manhattan plot illustrating SNPs linked to yield with their corresponding statistical significance represented by Q-Q plot

Supplementary Figure7: Manhattan plot illustrating SNPs linked to specific chickpea traits, along with their corresponding statistical significance represented by Q-Q plot for location 3 identified from Blink model

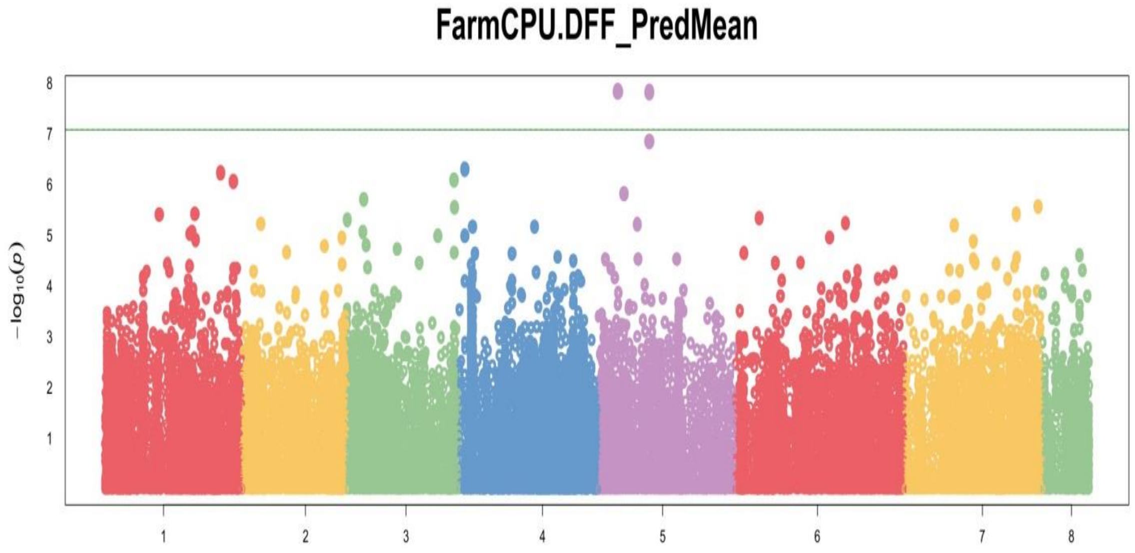

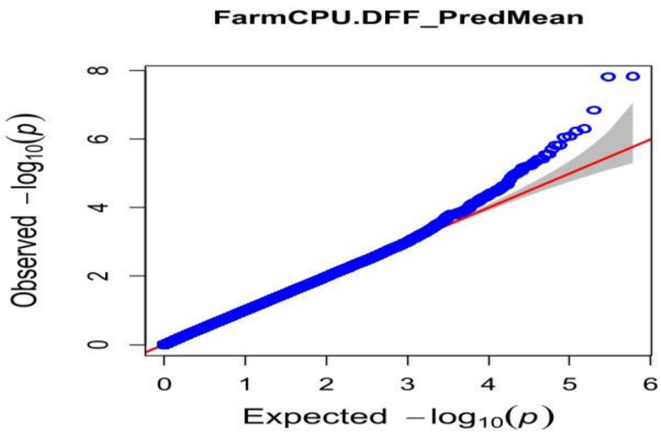

Supplementary Figure8a:Manhattan plot illustrating SNPs linked to Days fifty percentage flowering along with their corresponding statistical significance represented by Q-Q plot.

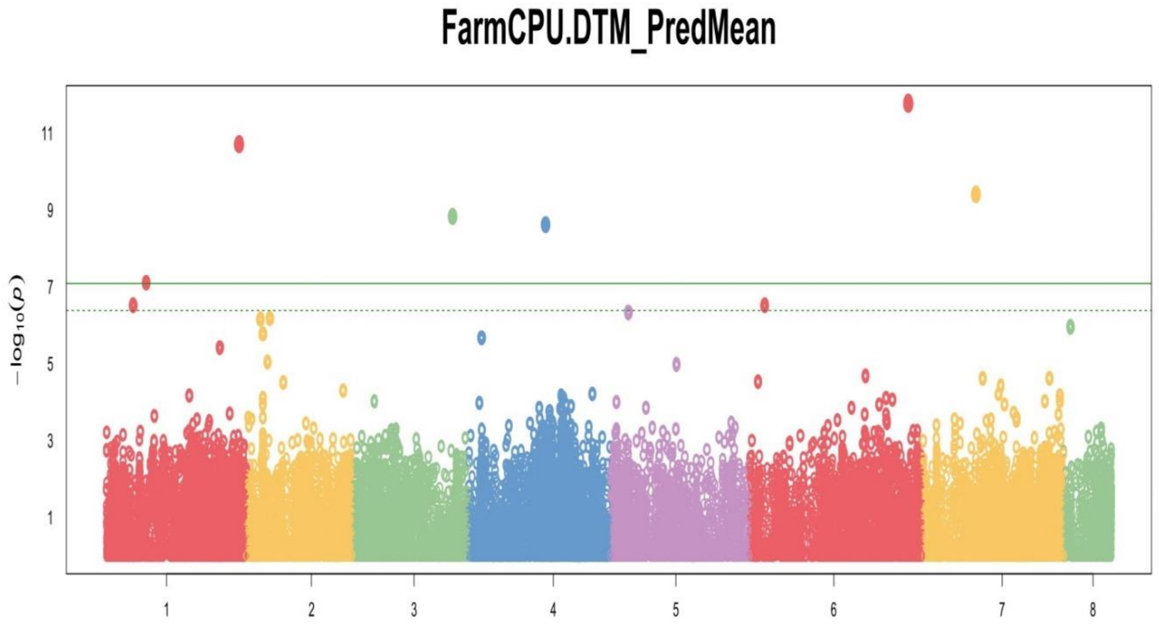

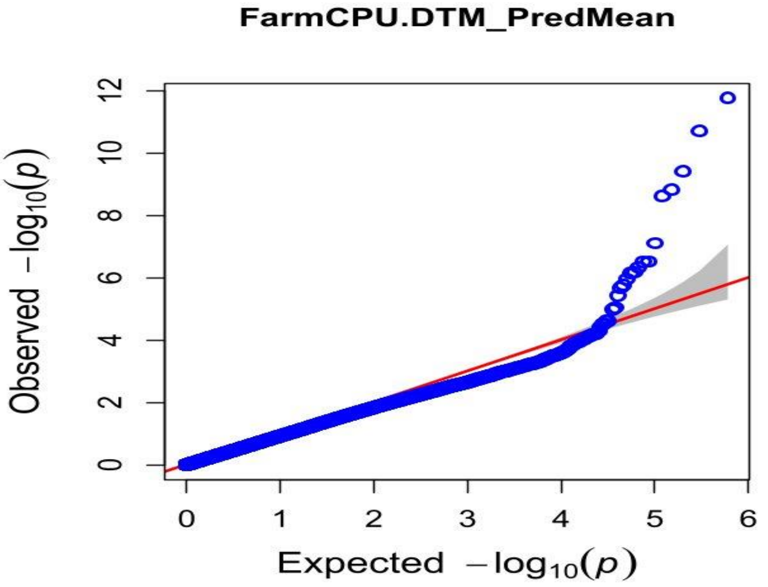

Supplementary Figure8b: Manhattan plot illustrating SNPs linked to Days to maturity along with their corresponding statistical significance represented by Q-Q plot

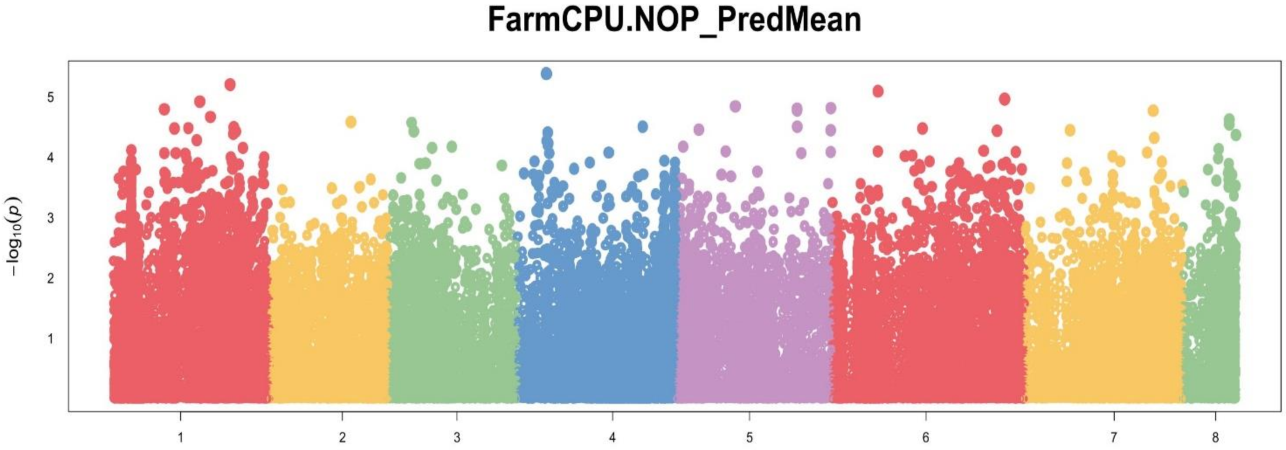

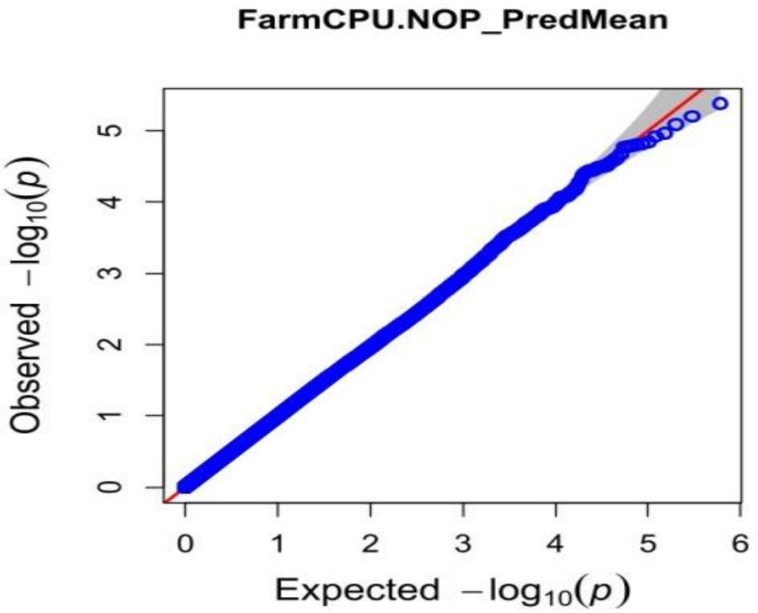

Supplementary Figure8c:Manhattan plot illustrating SNPs linked to number of pods along with their corresponding statistical significance represented by Q-Q plot

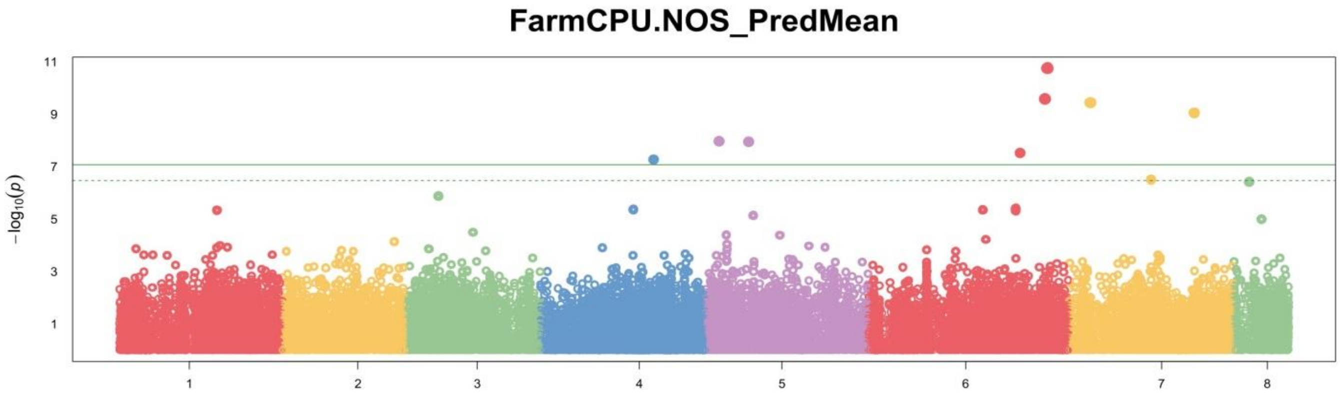

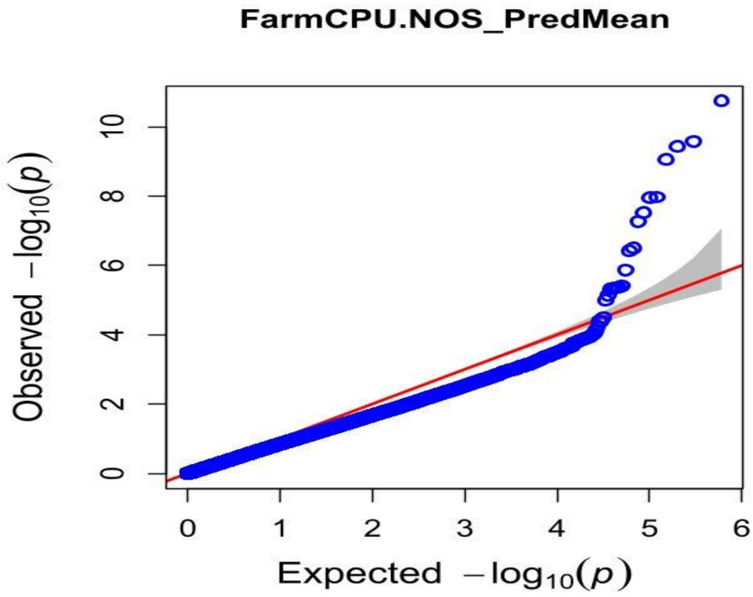

Supplementary Figure8d: Manhattan plot illustrating SNPs linked to number of seeds along with their corresponding statistical significance represented by Q-Q plot

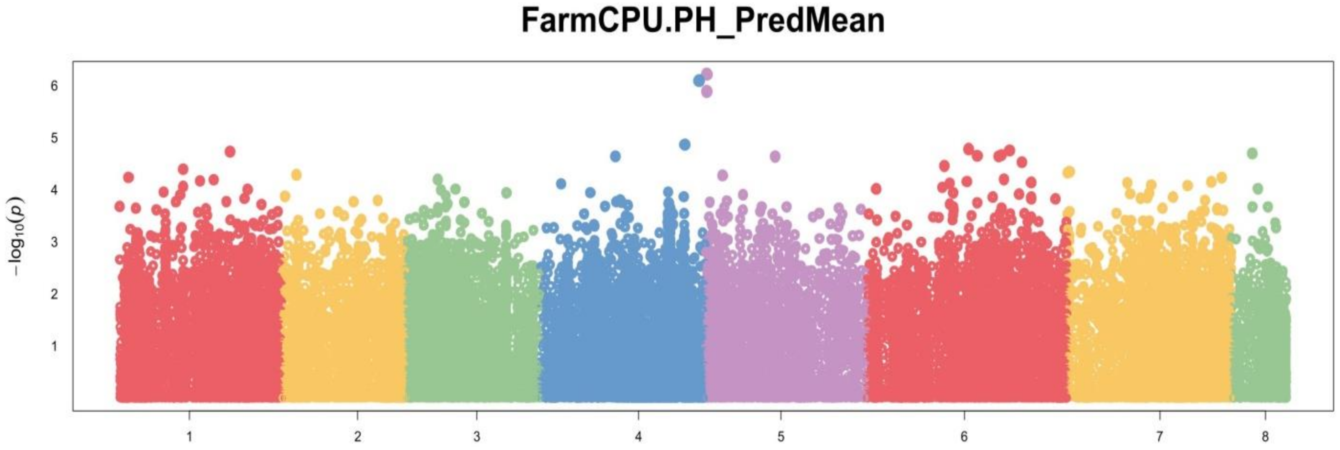

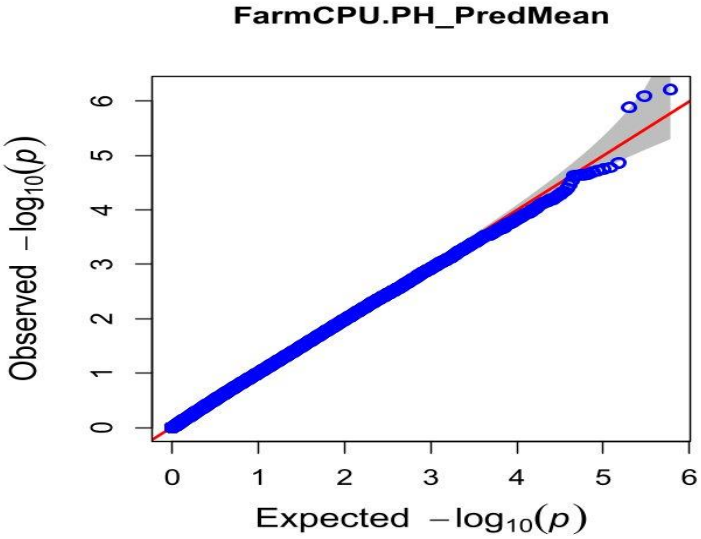

Supplementary Figure8e: Manhattan plot illustrating SNPs linked to plant height along with their corresponding statistical significance represented by Q-Q plot

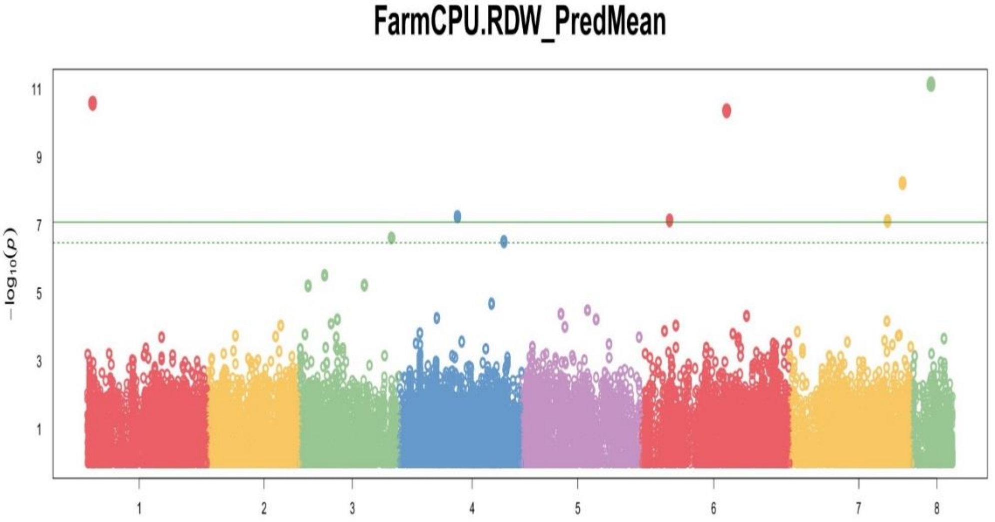

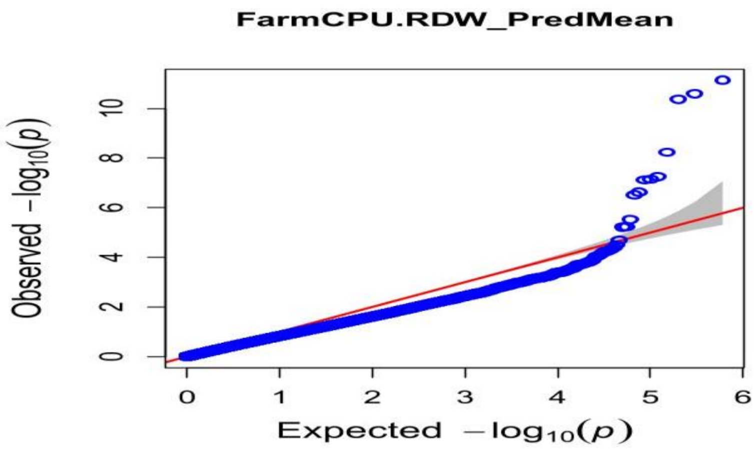

Supplementary Figure8f:Manhattan plot illustrating SNPs linked to root dry weight with their corresponding statistical significance represented by Q-Q plot

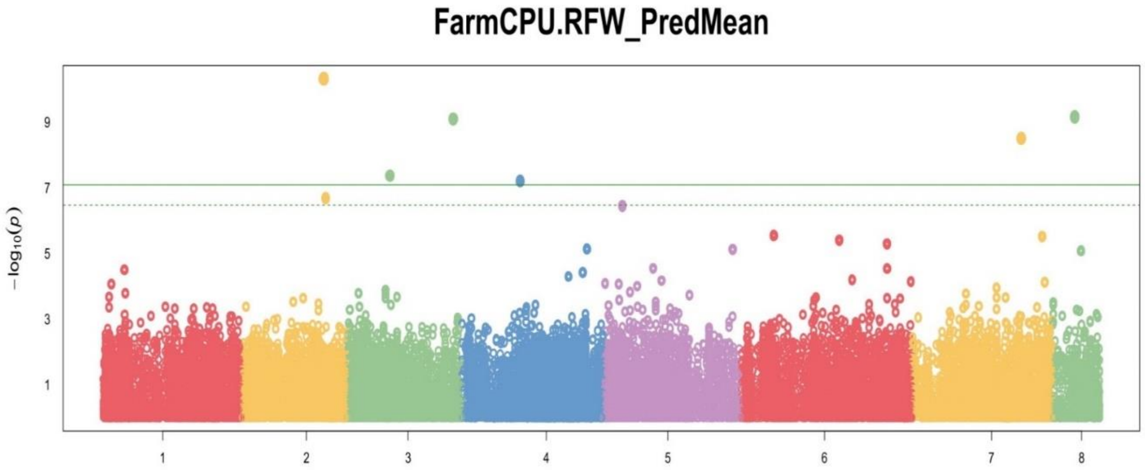

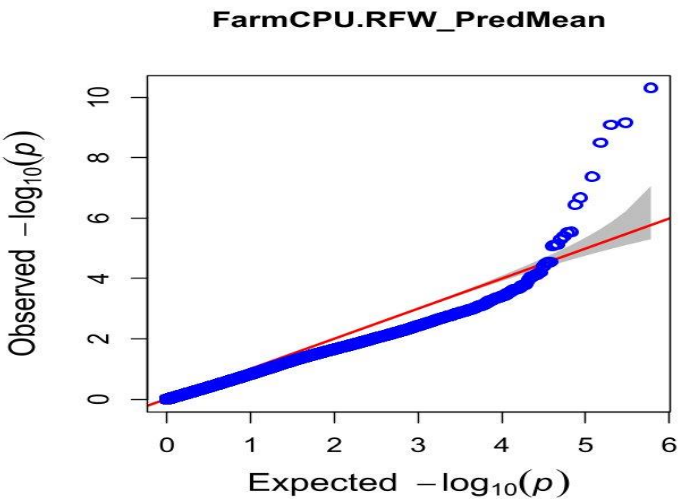

Supplementary Figure8g: Manhattan plot illustrating SNPs linked to root fresh weight with their corresponding statistical significance represented by Q-Q plot

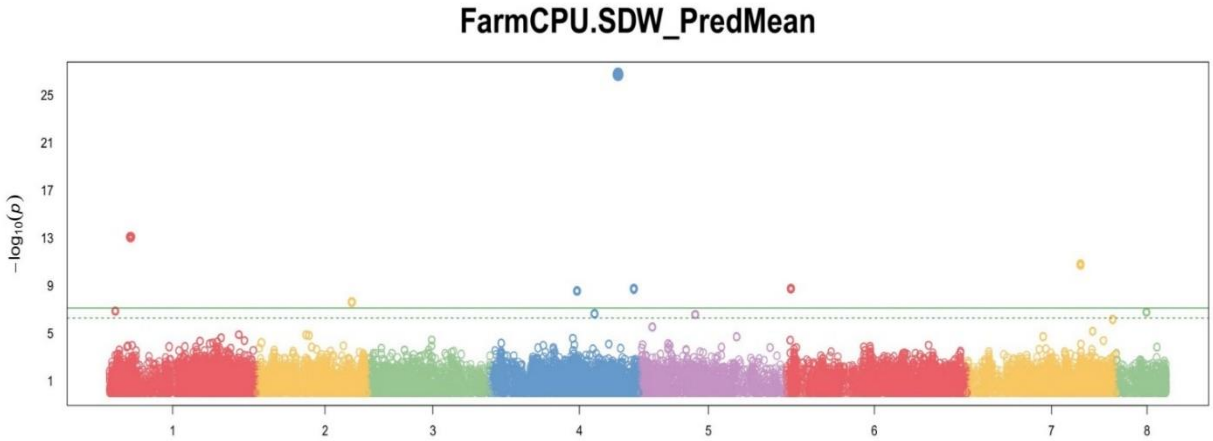

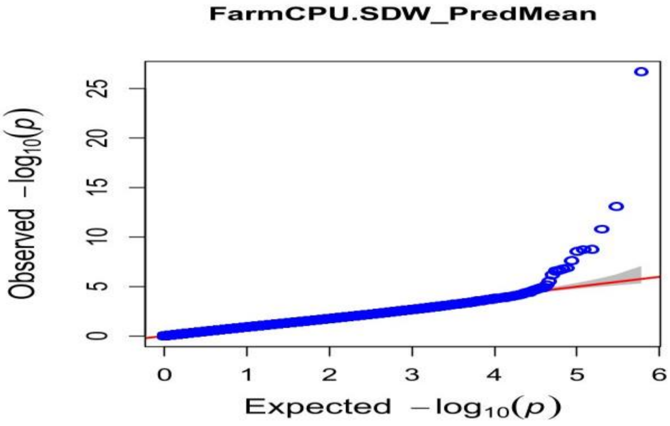

Supplementary Figure8h: Manhattan plot illustrating SNPs linked to shoot dry weight with their corresponding statistical significance represented by Q-Q plot

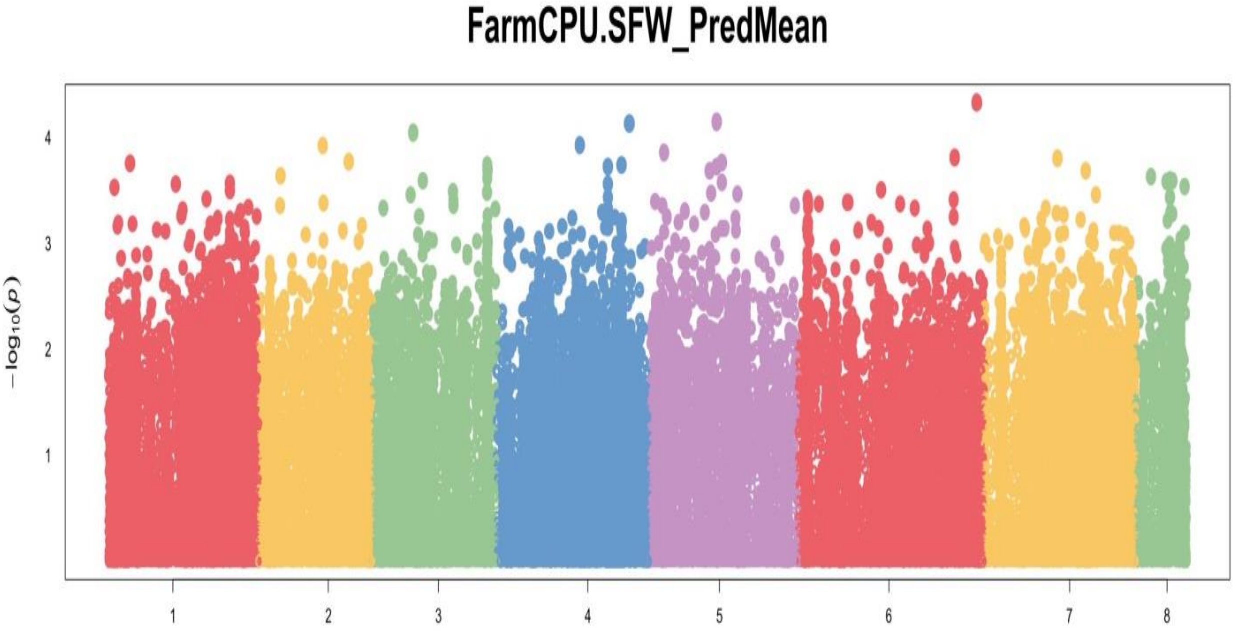

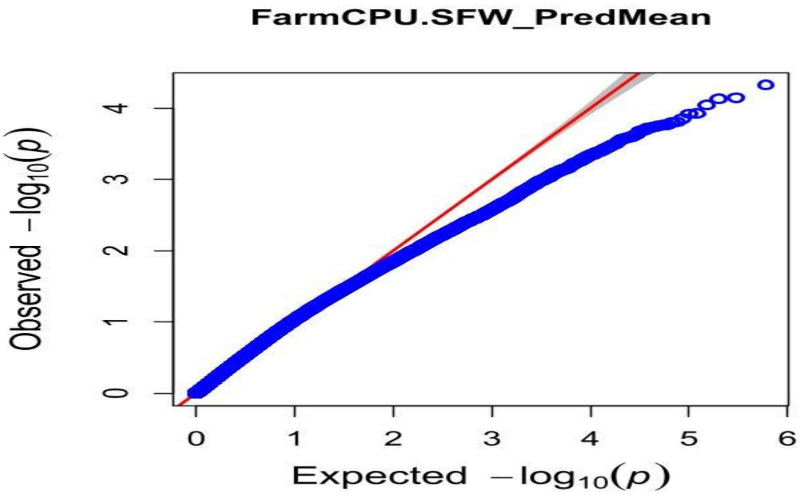

Supplementary Figure8i: Manhattan plot illustrating SNPs linked to shoot fresh weight with their corresponding statistical significance represented by Q-Q plot

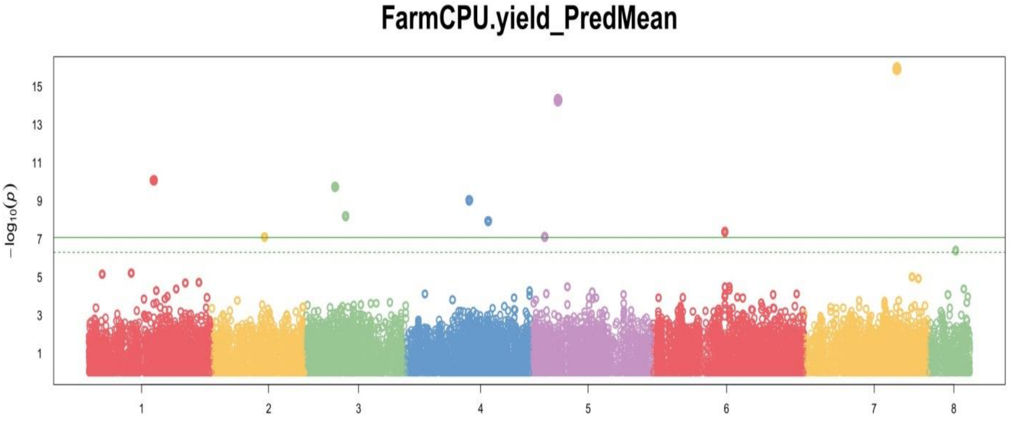

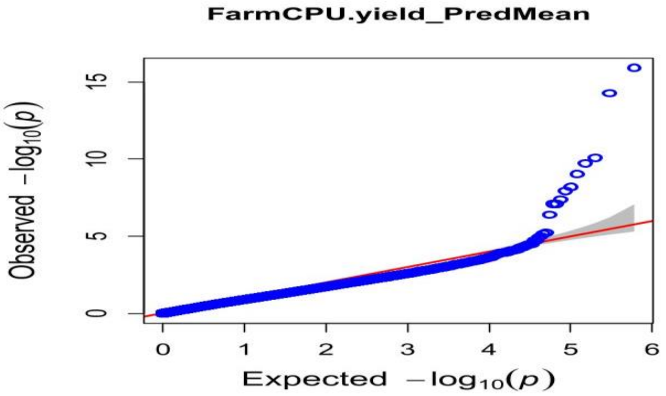

Supplementary Figure8j: Manhattan plot illustrating SNPs linked to yield with their corresponding statistical significance represented by Q-Q plot

Supplementary Figure8: Manhattan plot illustrating SNPs linked to specific chickpea traits, along with their corresponding statistical significance represented by Q-Q plot for location 3 identified from FarmCPU model

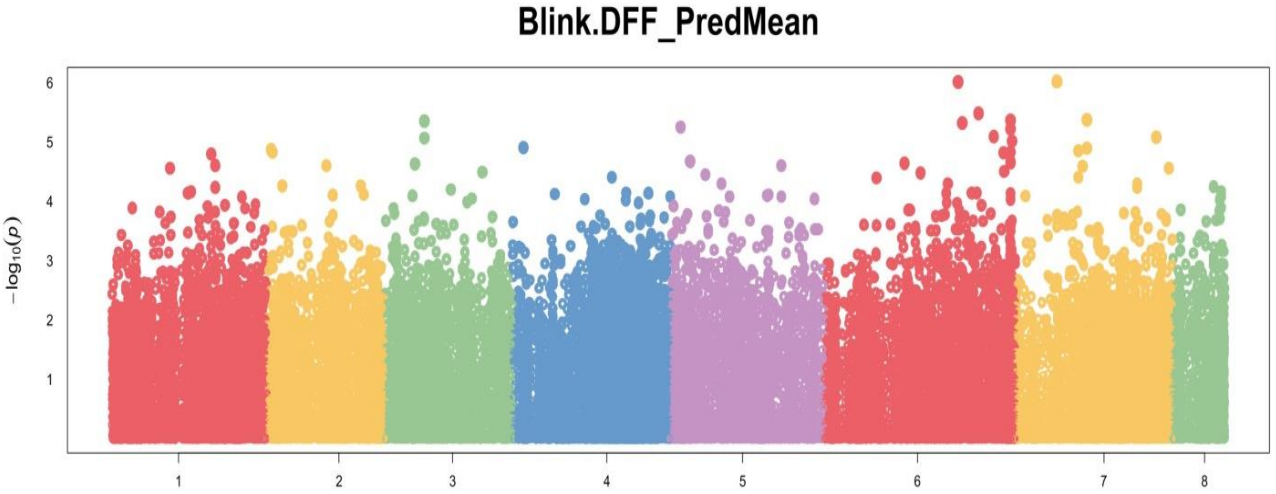

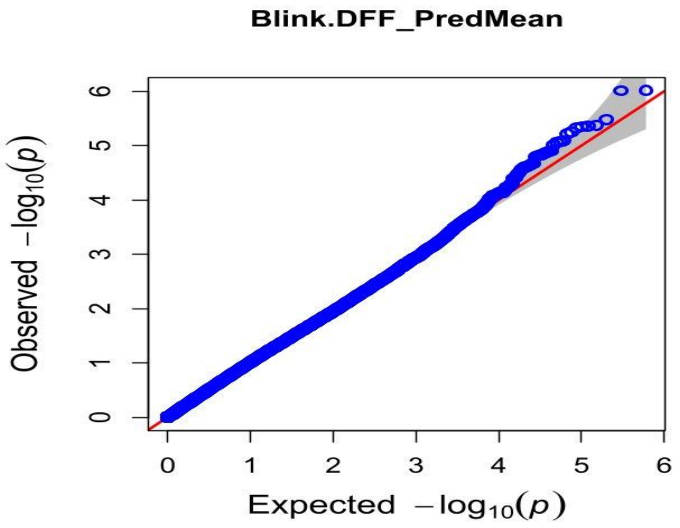

Supplementary Figure9a: Manhattan plot illustrating SNPs linked to Days fifty percentage flowering along with their corresponding statistical significance represented by Q-Q plot

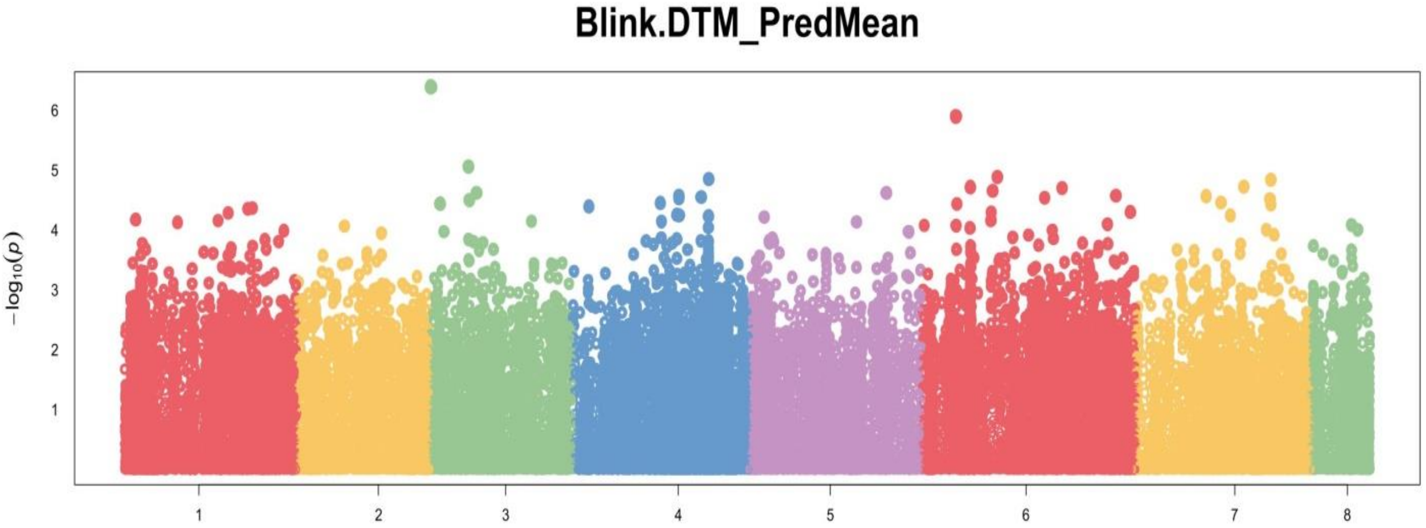

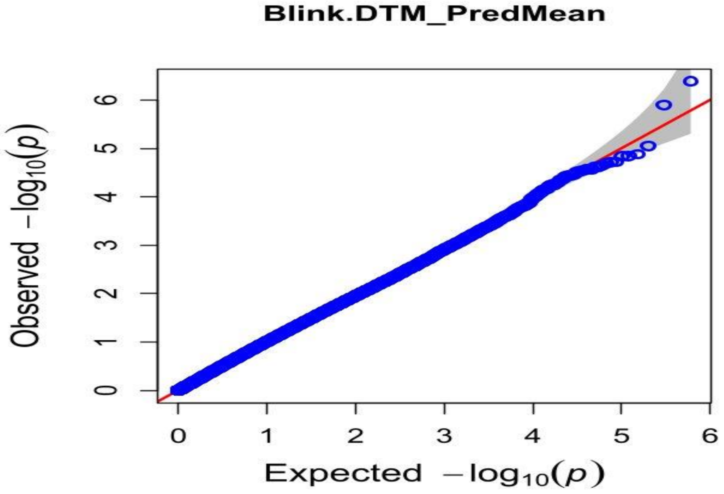

Supplementary Figure9b: Manhattan plot illustrating SNPs linked to Days to maturity along with their corresponding statistical significance represented by Q-Q plot

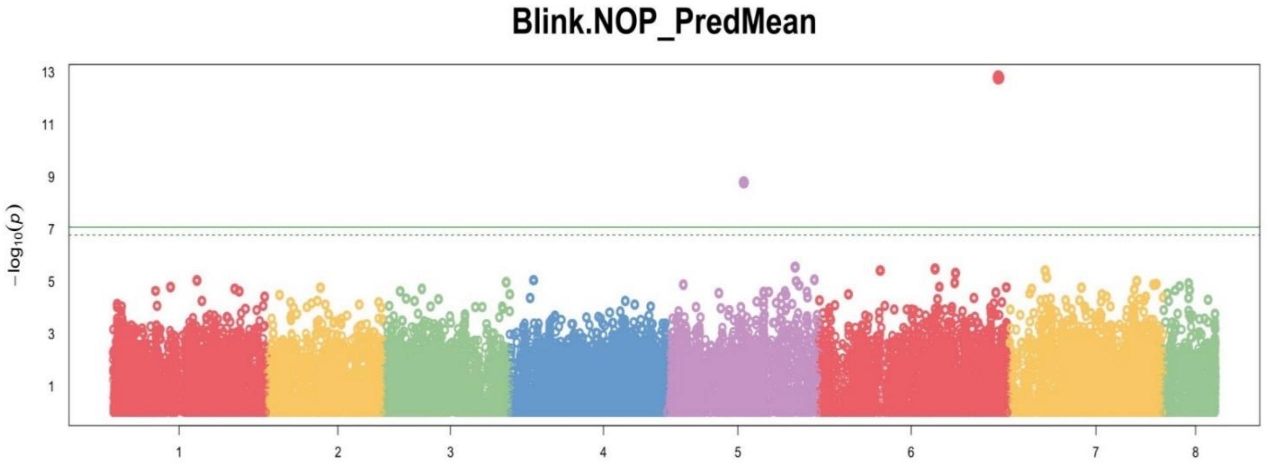

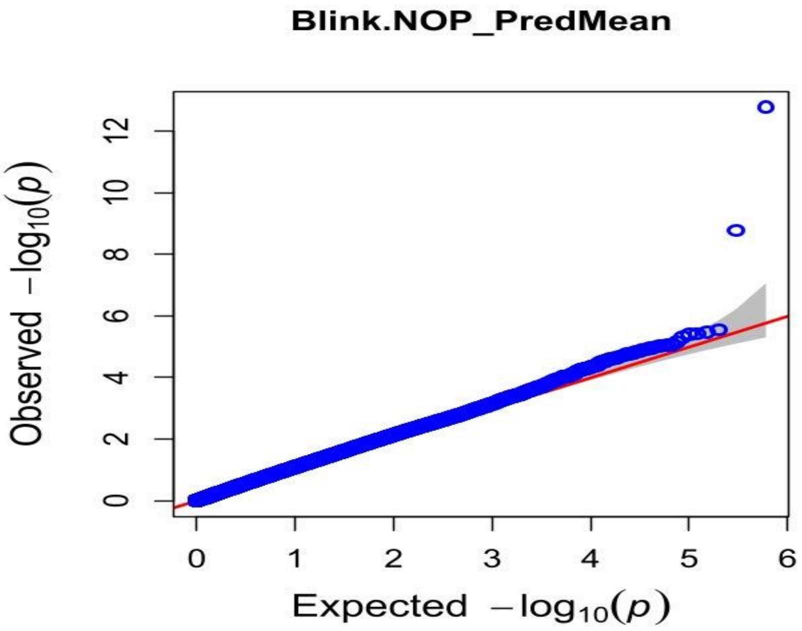

Supplementary Figure9c: Manhattan plot illustrating SNPs linked to number of pods along with their corresponding statistical significance represented by Q-Q plot

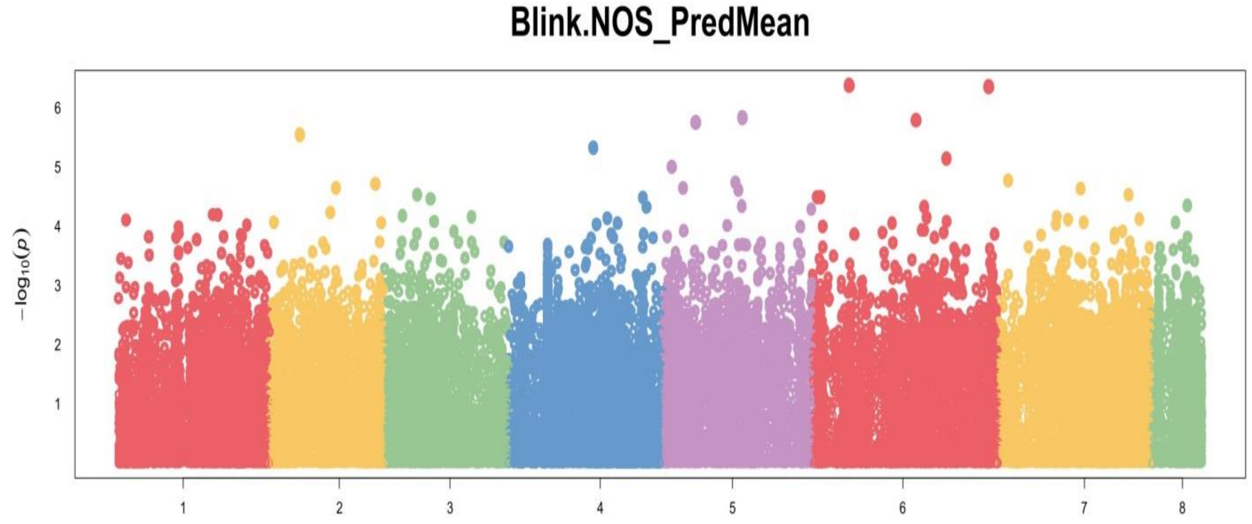

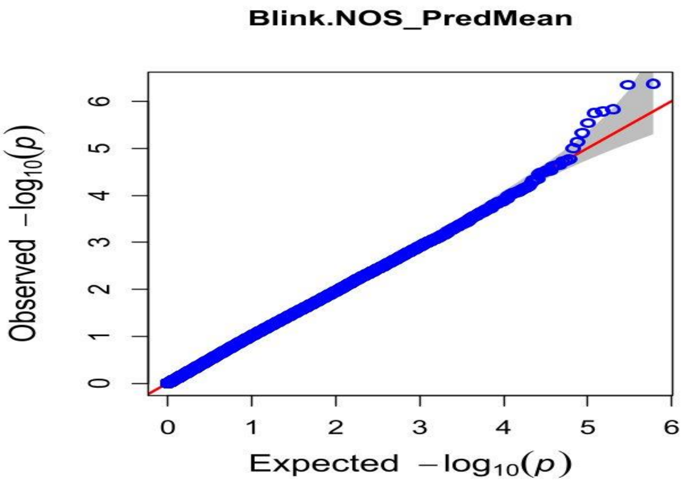

Supplementary Figure9d: Manhattan plot illustrating SNPs linked to number of seeds along with their corresponding statistical significance represented by Q-Q plot.

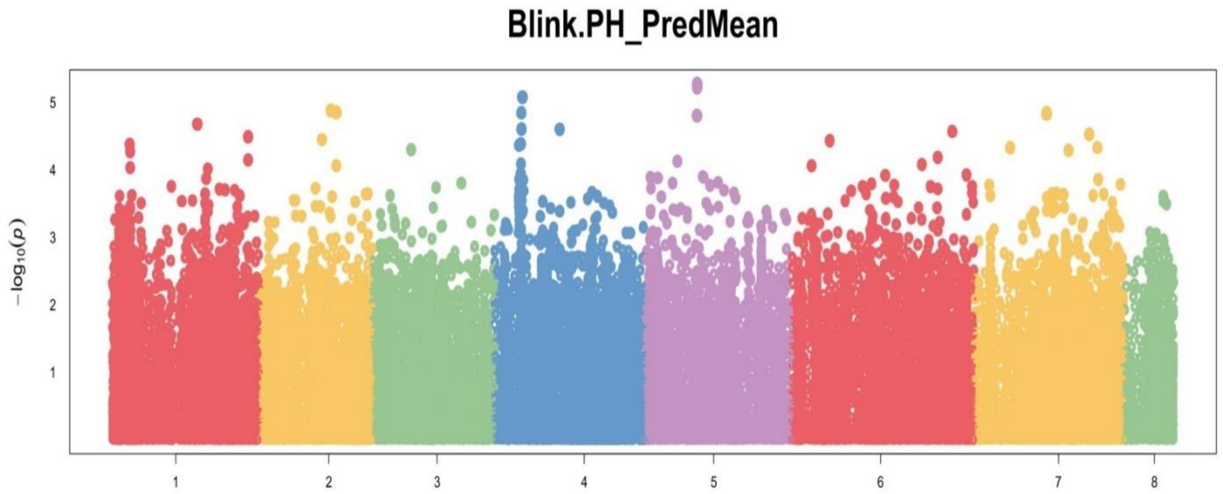

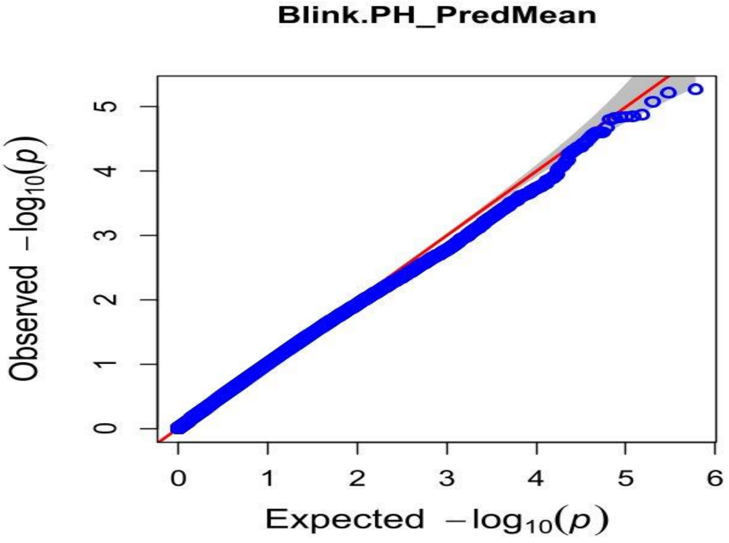

Supplementary Figure9e: Manhattan plot illustrating SNPs linked to plant height along with their corresponding statistical significance represented by Q-Q plot

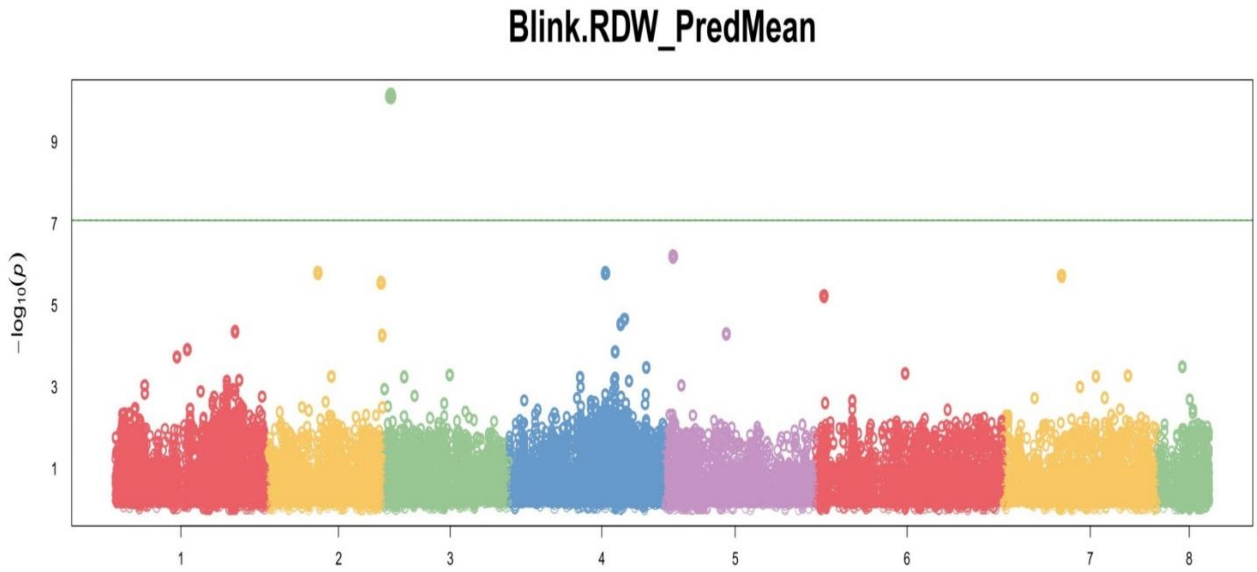

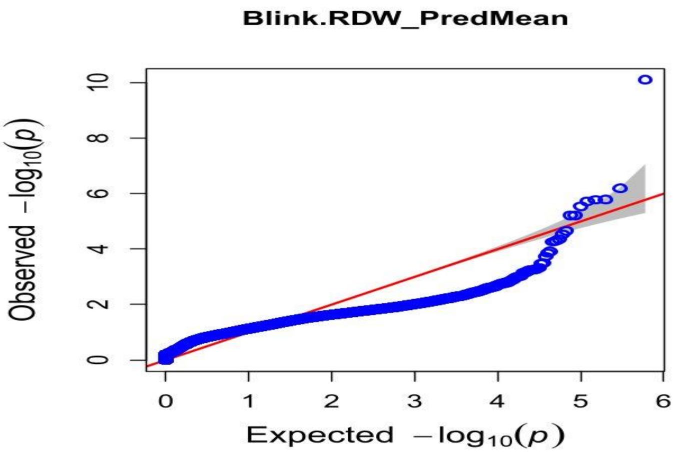

Supplementary Figure9f: Manhattan plot illustrating SNPs linked to root dry weight with their corresponding statistical significance represented by Q-Q plot

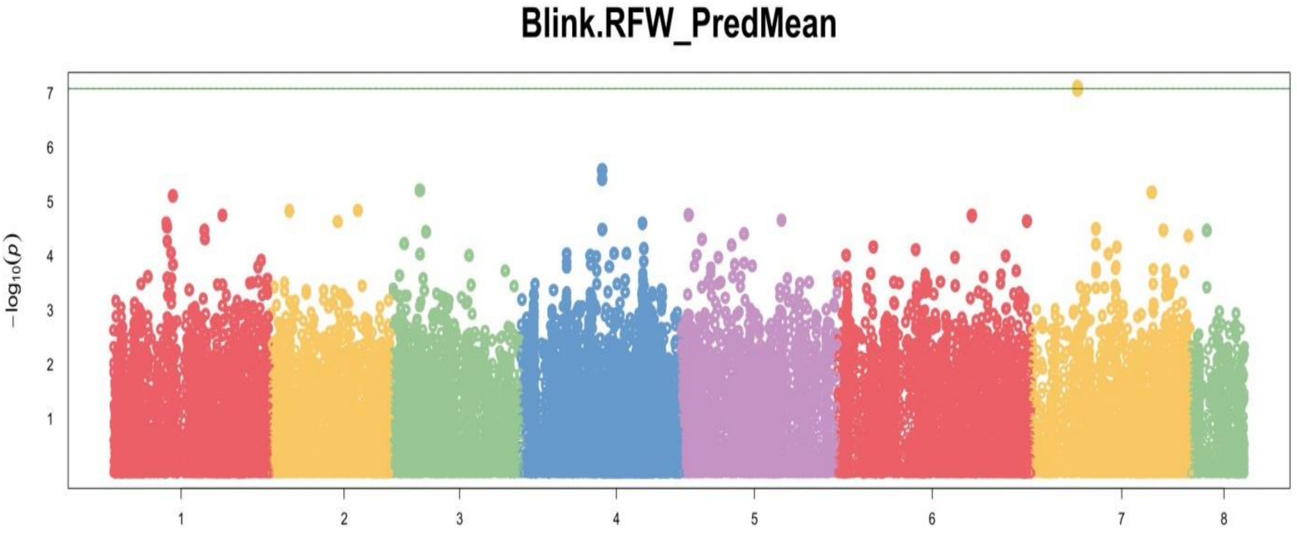

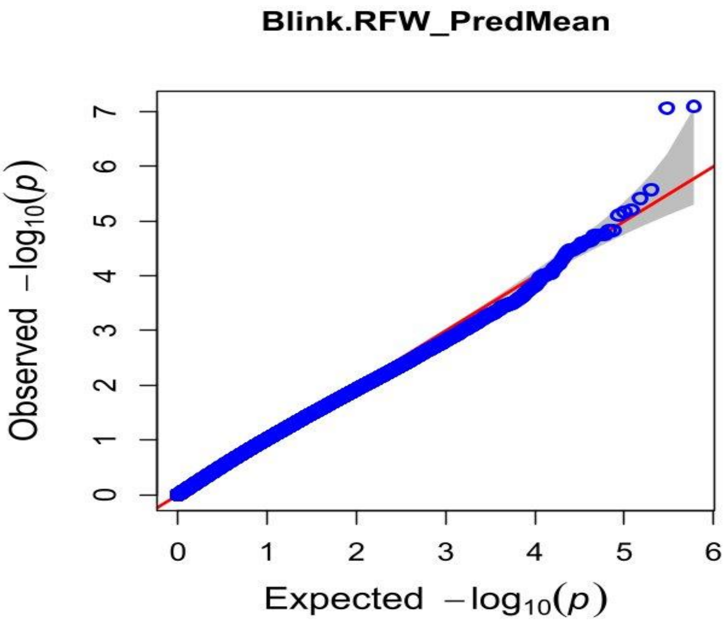

Supplementary Figure9f: Manhattan plot illustrating SNPs linked to root fresh weight with their corresponding statistical significance represented by Q-Q plot

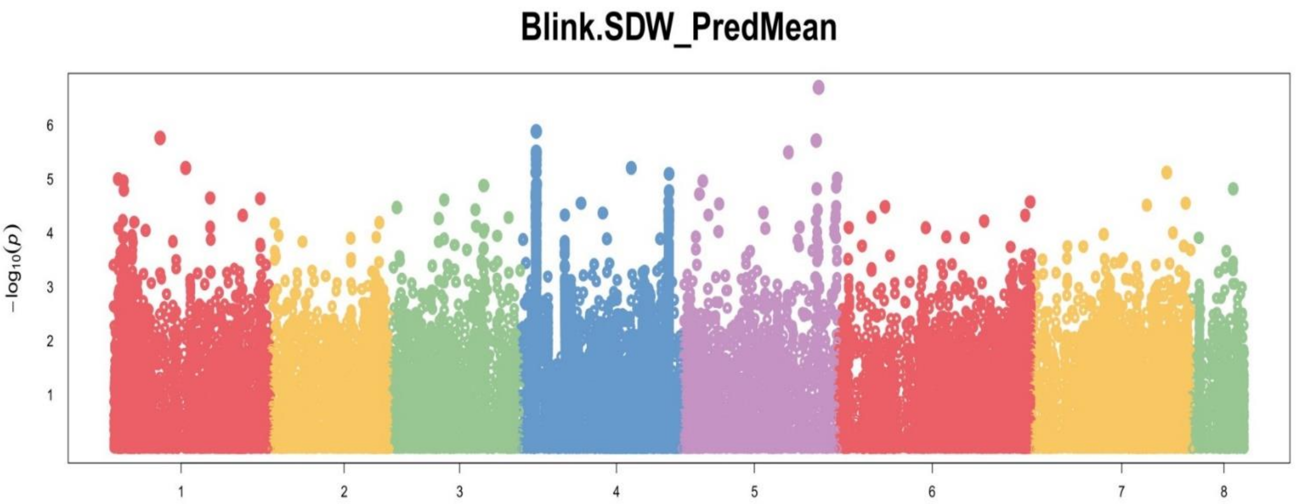

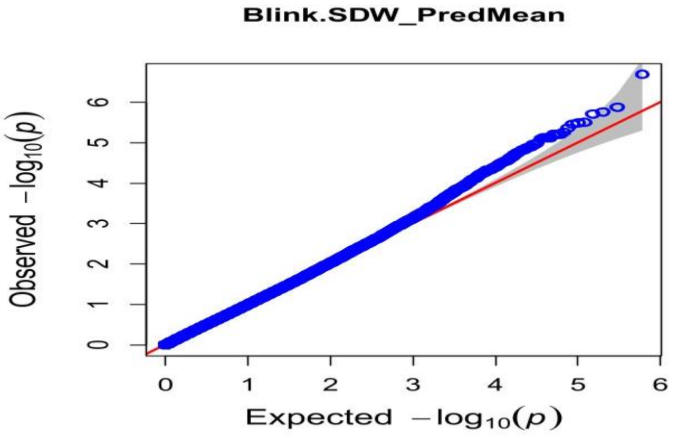

Supplementary Figure9h: Manhattan plot illustrating SNPs linked to r shoot dry weight with their corresponding statistical significance represented by Q-Q plot

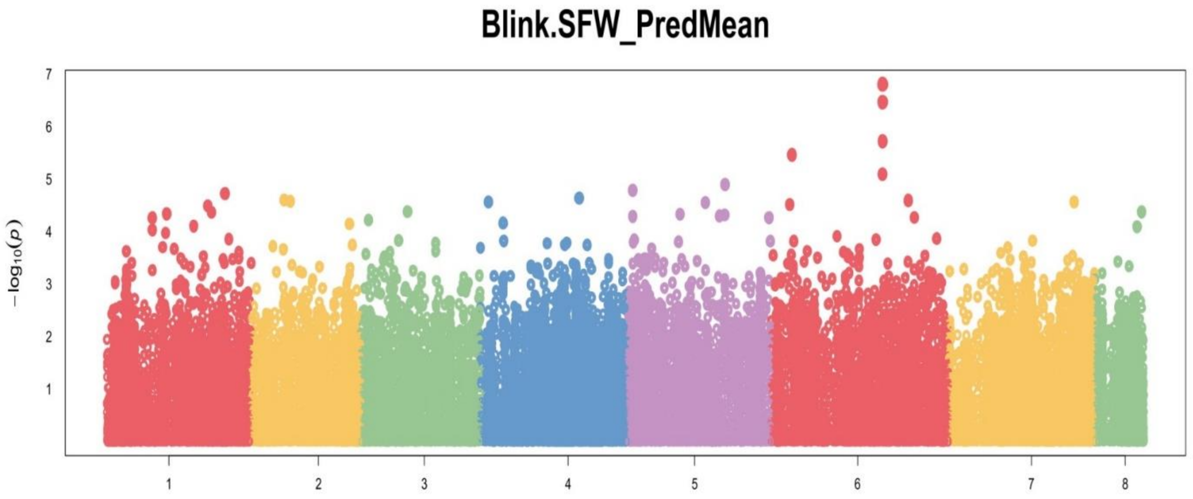

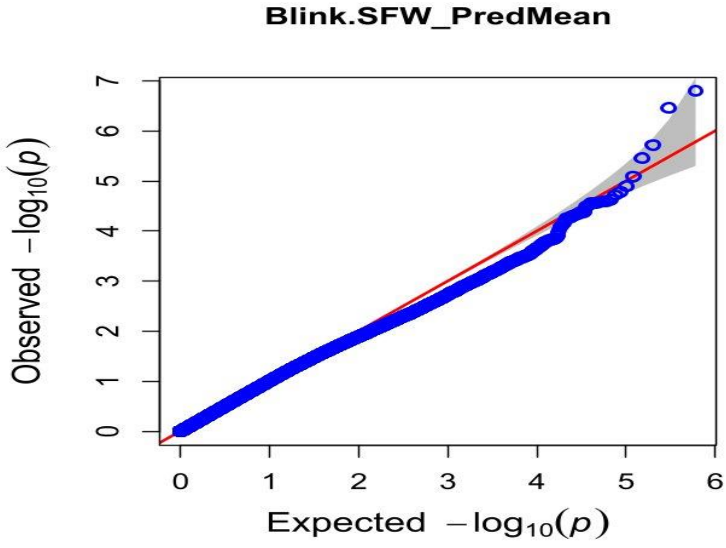

Supplementary Figure9i: Manhattan plot illustrating SNPs linked to shoot fresh weight with their corresponding statistical significance represented by Q-Q plot

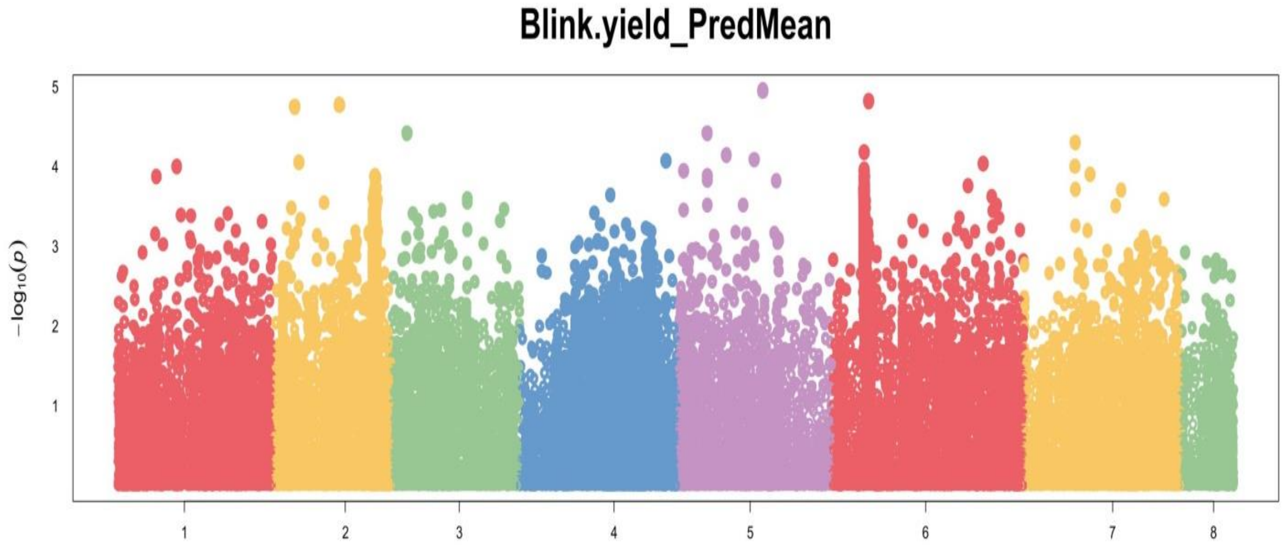

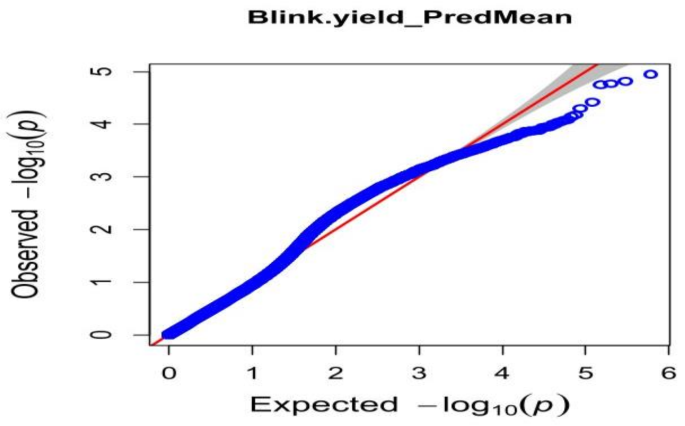

Supplementary Figure9j: Manhattan plot illustrating SNPs linked to yield with their corresponding statistical significance represented by Q-Q plot

Supplementary Figure9: Manhattan plot illustrating SNPs linked to specific chickpea traits, along with their corresponding statistical significance represented by Q-Q plot for location 4 identified from Blink model

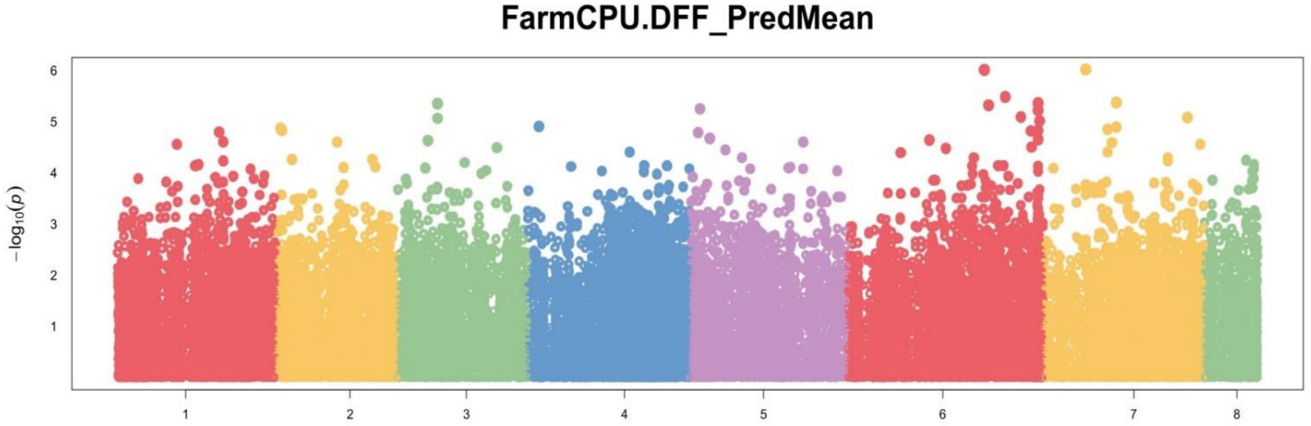

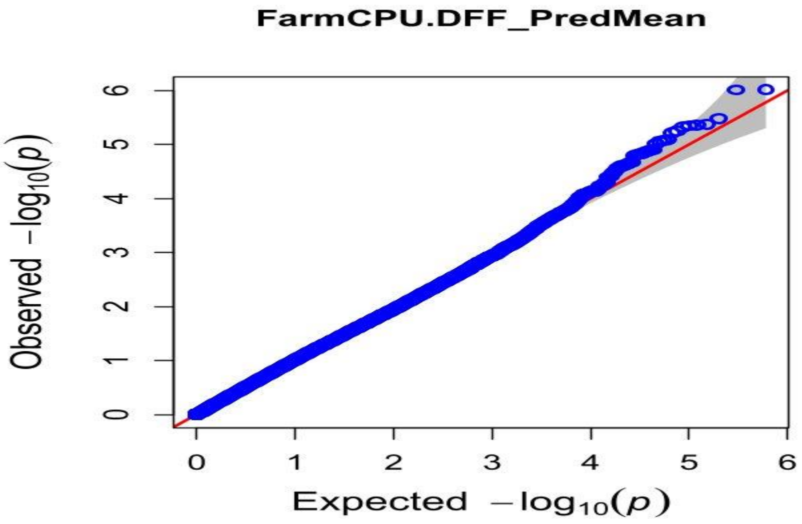

Supplementary Figure10a: Manhattan plot illustrating SNPs linked to Days fifty percentage flowering along with their corresponding statistical significance represented by Q-Q plot.

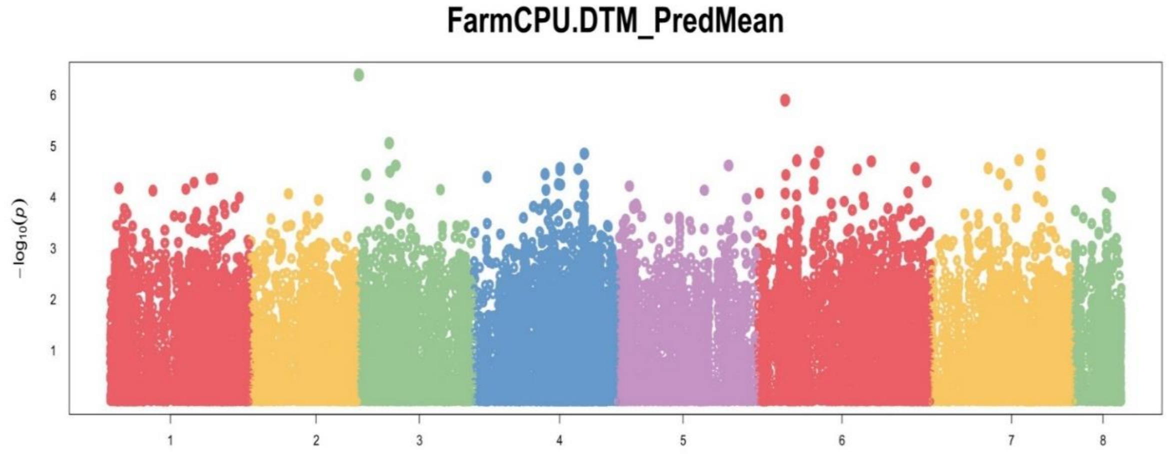

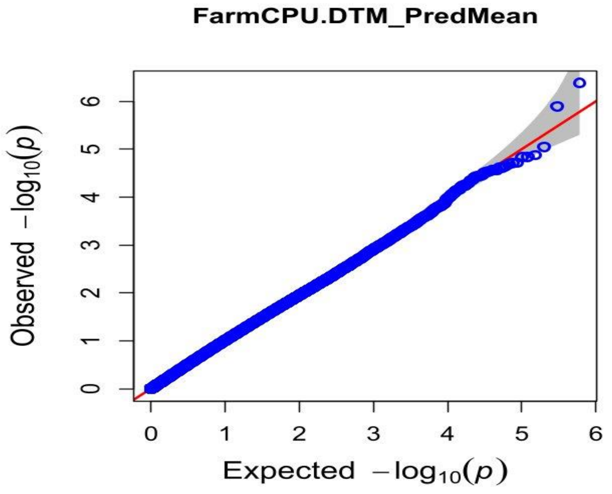

Supplementary Figure10b: Manhattan plot illustrating SNPs linked to Days to maturity along with their corresponding statistical significance represented by Q-Q plot

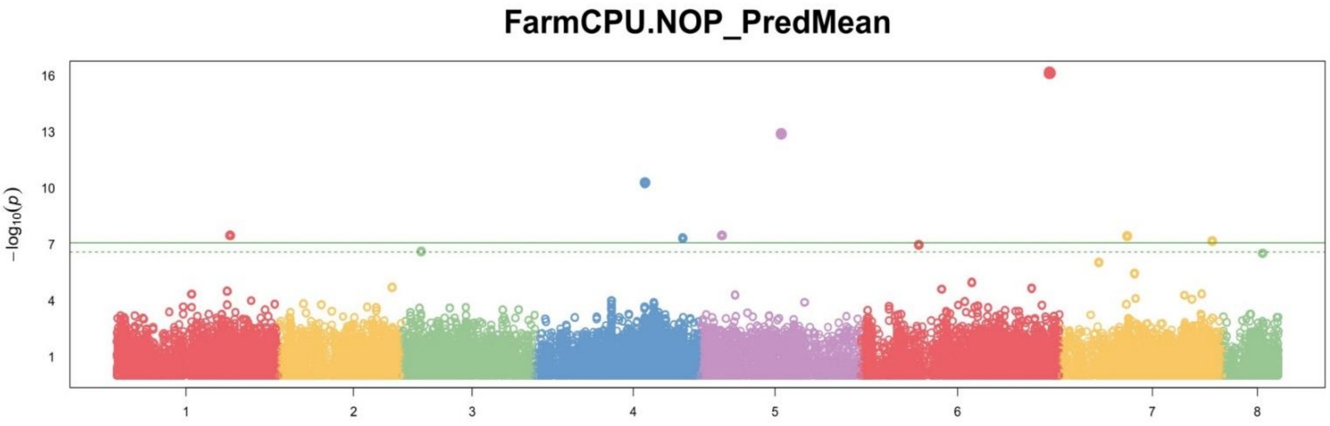

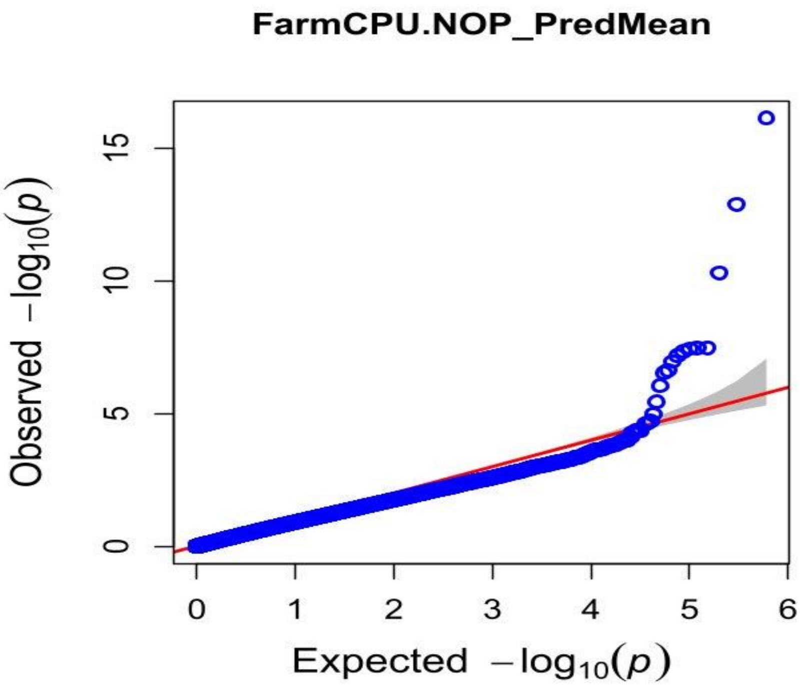

Supplementary Figure10b: Manhattan plot illustrating SNPs linked to number of along with their corresponding statistical significance represented by Q-Q plot

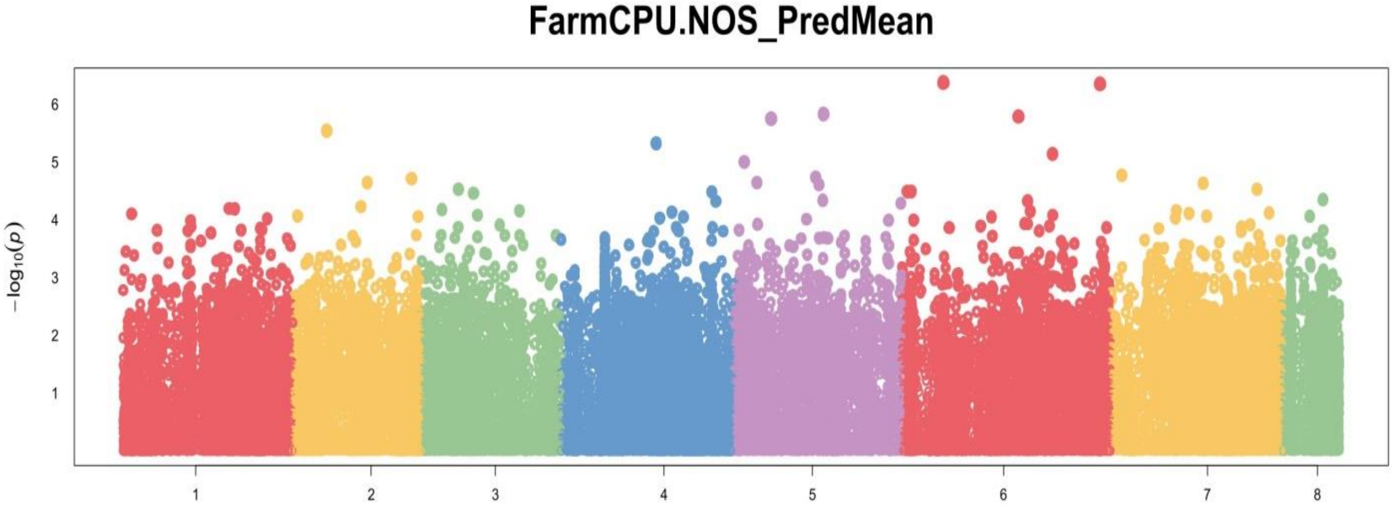

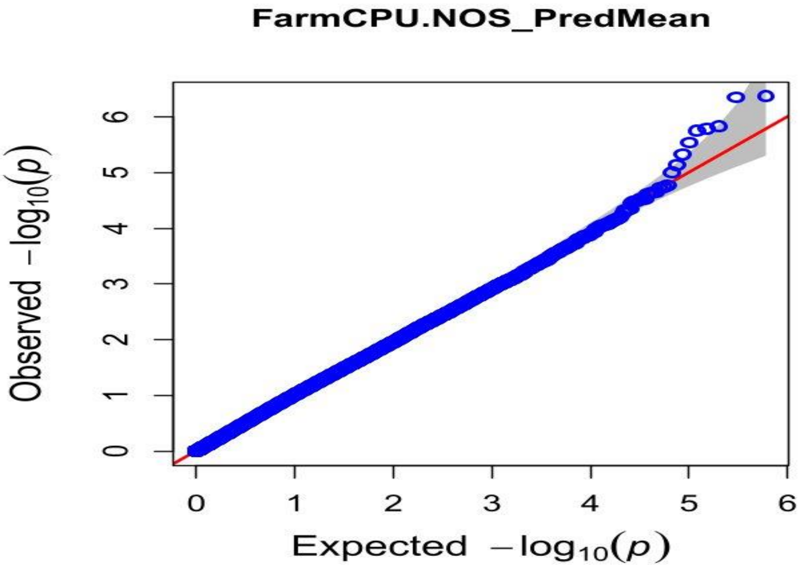

Supplementary Figure10d: Manhattan plot illustrating SNPs linked to number of seeds along with their corresponding statistical significance represented by Q-Q plot

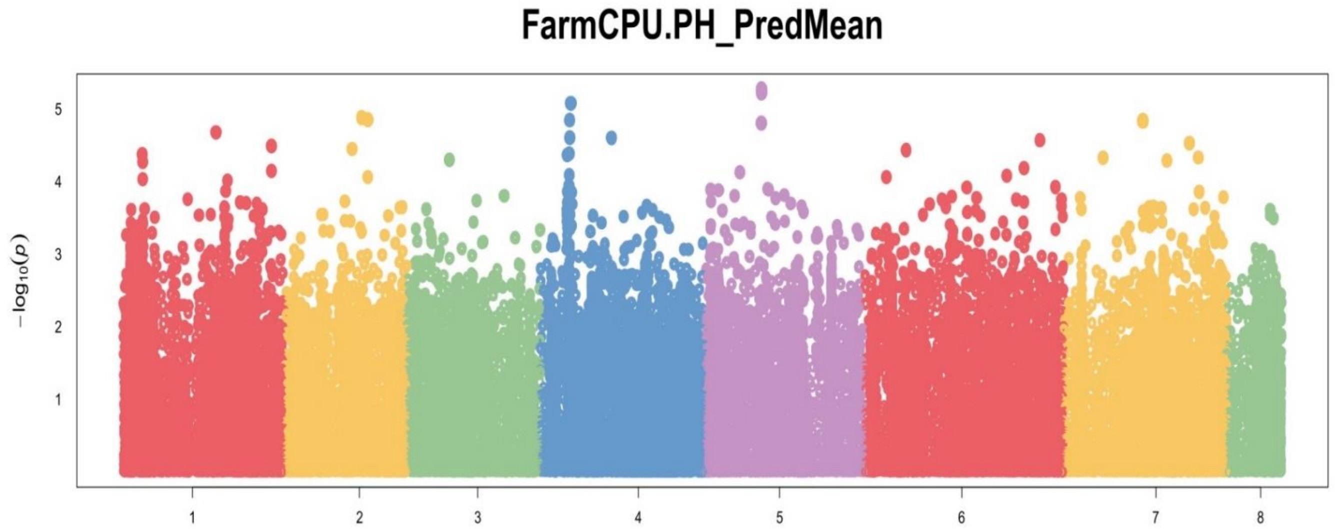

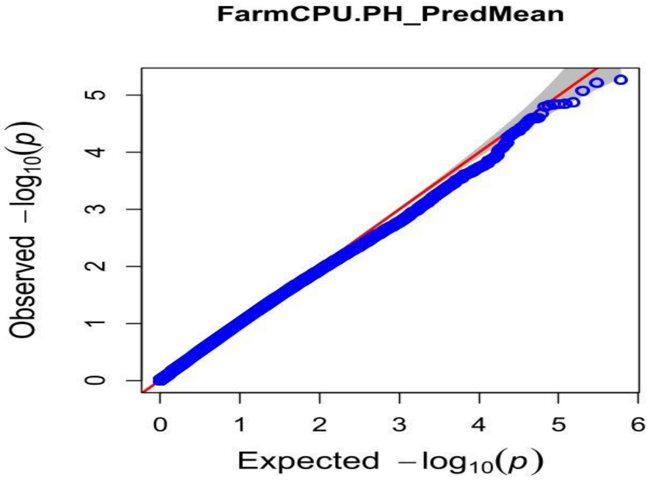

Supplementary Figure10e: Manhattan plot illustrating SNPs linked to plant height along with their corresponding statistical significance represented by Q-Q plot

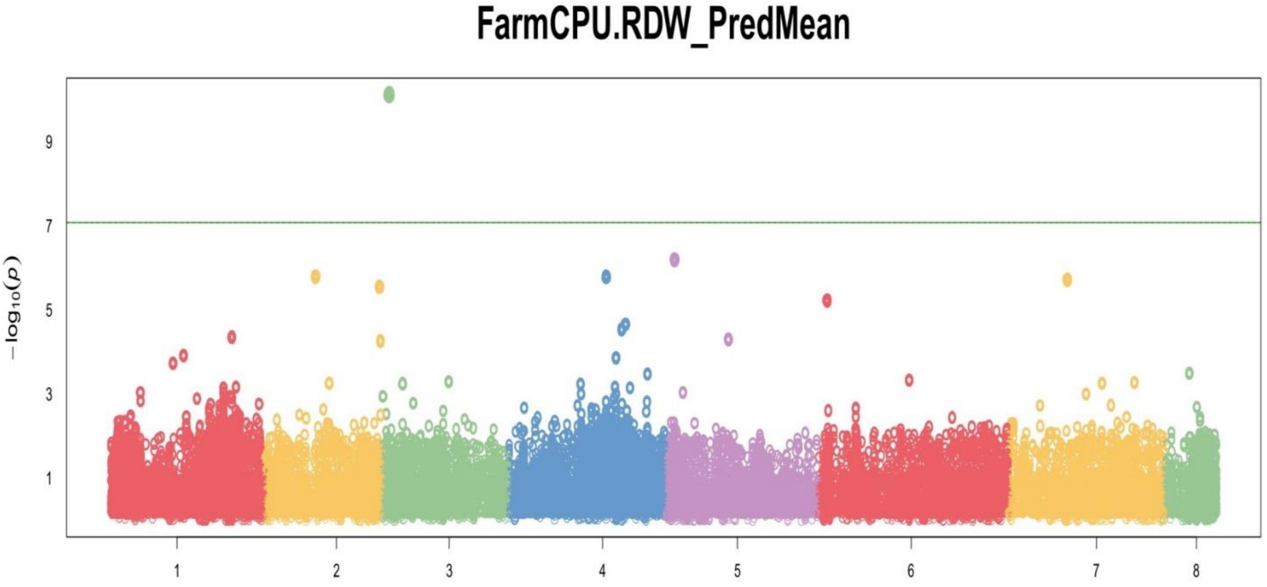

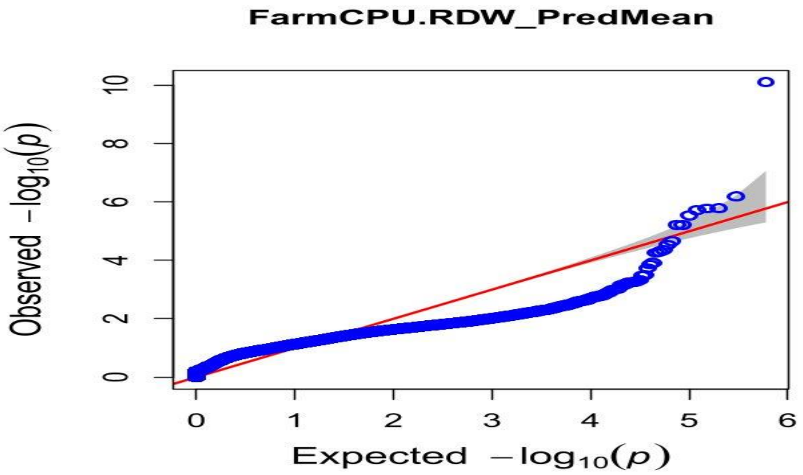

Supplementary Figure10f: Manhattan plot illustrating SNPs linked to root dry weight with their corresponding statistical significance represented by Q-Q plot

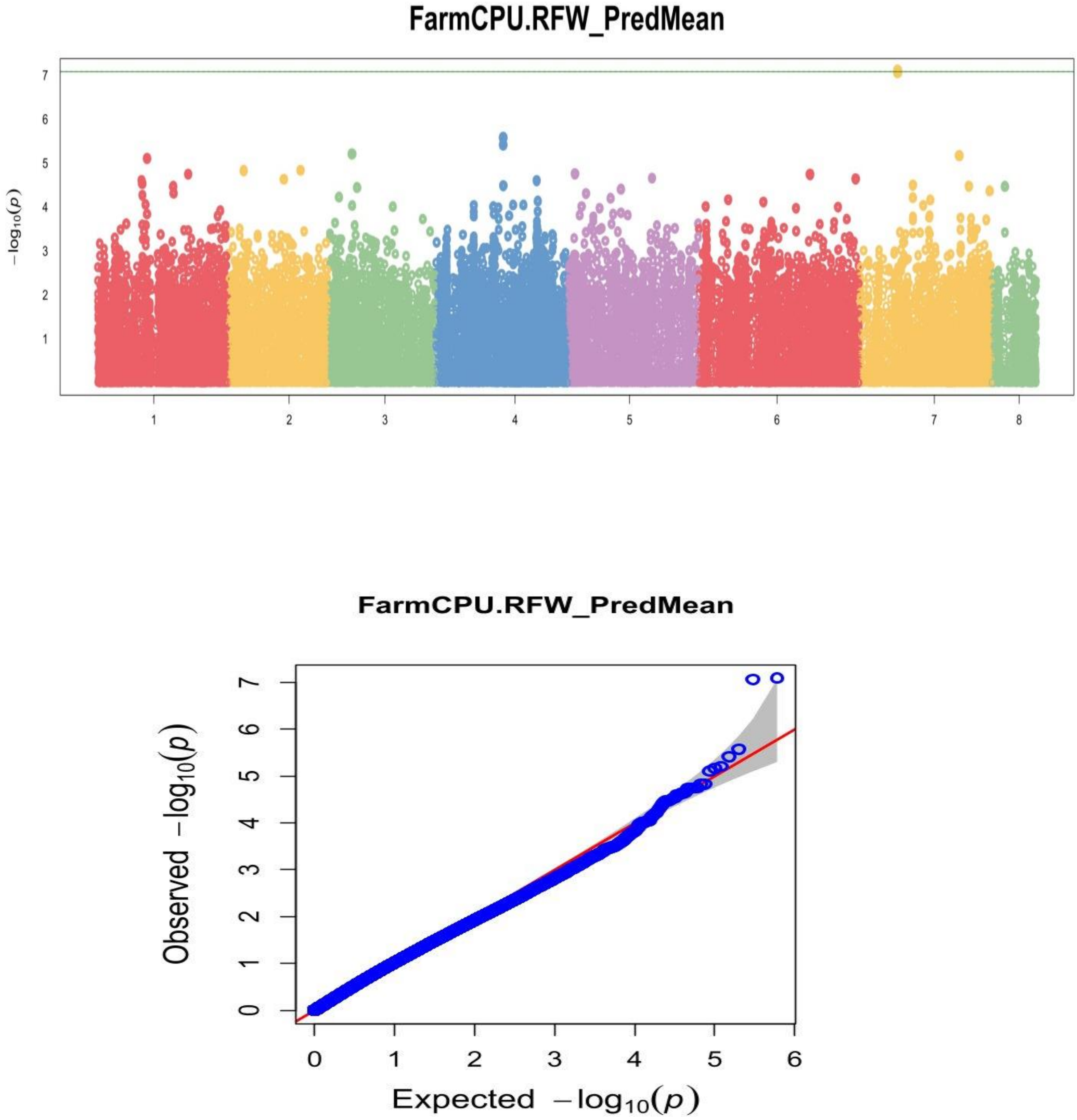

Supplementary Figure10g: Manhattan plot illustrating SNPs linked to root fresh weight with their corresponding statistical significance represented by Q-Q plot

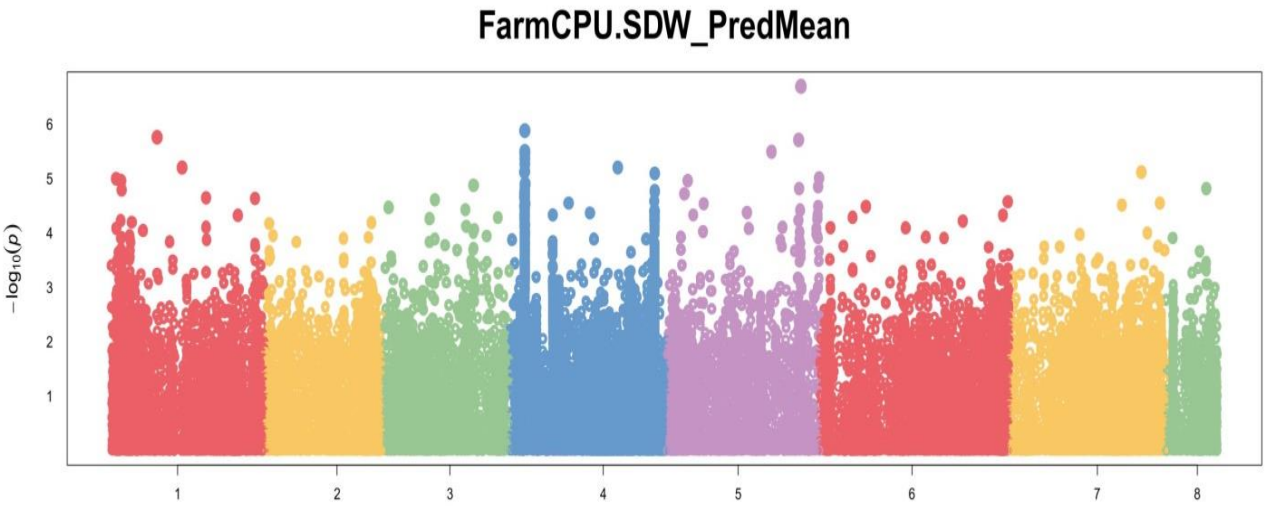

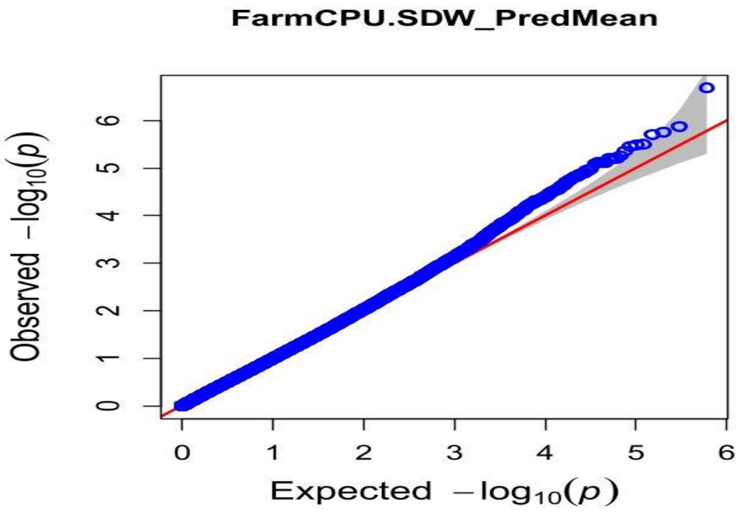

Supplementary Figure10h: Manhattan plot illustrating SNPs linked to shoot dry weight with their corresponding statistical significance represented by Q-Q plot

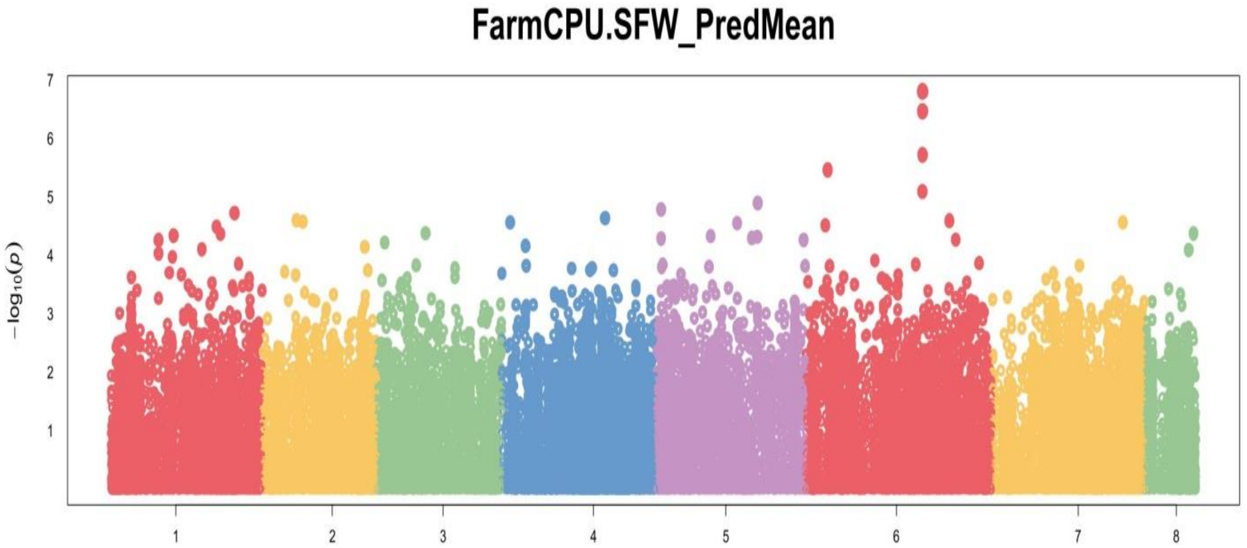

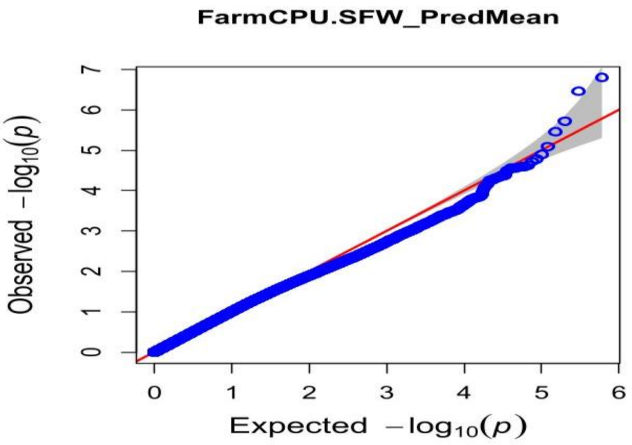

Supplementary Figure10i: Manhattan plot illustrating SNPs linked to shoot fresh weight with their corresponding statistical significance represented by Q-Q plot

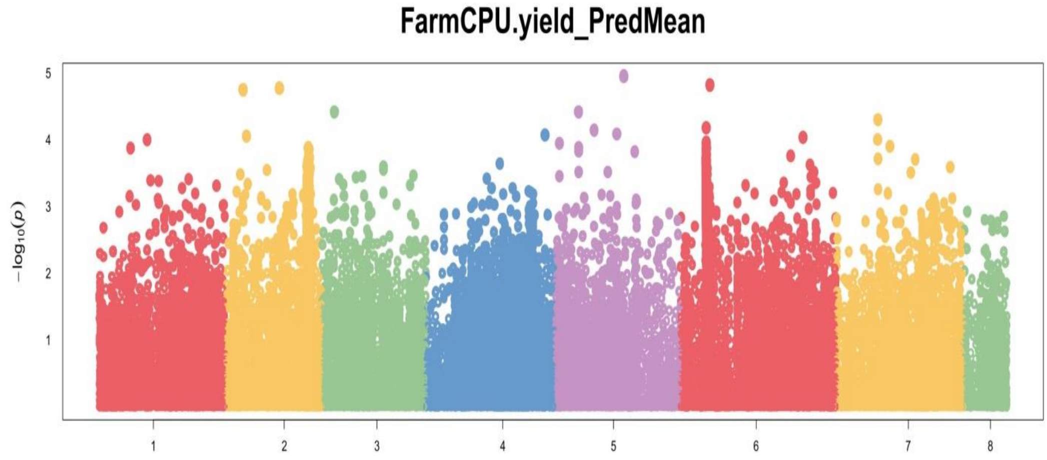

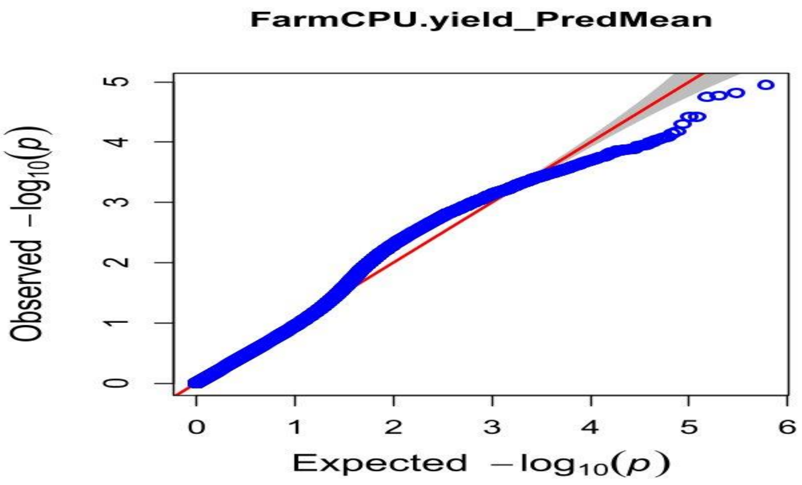

Supplementary Figure10j: Manhattan plot illustrating SNPs linked to yield with their corresponding statistical significance represented by Q-Q plot

Supplementary Figure10: Manhattan plot illustrating SNPs linked to specific chickpea traits, along with their corresponding statistical significance represented by Q-Q plot for location 4 identified from FarmCPU model
