# Supplementary material for: Genome-wide association mapping identifies novel SNPs for root nodulation and agronomic traits in chickpea
Source: Front Plant Sci. 2024 Oct 15;15:1395938. doi: 10.3389/fpls.2024.1395938 (PMC11518797; doi:10.3389/fpls.2024.1395938)
Supplement: Supplementary file 1 [file DataSheet1.zip › Supplementary Table-1.docx]

**Supplementary Table 1: List of the chickpea entries used in the present study**

| **S. No** | **Genotype Name** | **Origin** | **S. No** | **Genotype Name** | **Origin** | **S. No** | **Genotype Name** | **Origin** |
| --- | --- | --- | --- | --- | --- | --- | --- | --- |
| 1 | BG 372 | IARI, New Delhi, India | 51 | ICC1009 | Morocco | 101 | BG 1053 | IARI, New Delhi, India |
| 2 | ICC 1083 | Iran | 52 | ICC1013 | Morocco | 102 | BG 372 | IARI, New Delhi, India |
| 3 | ICC 1172 | India | 53 | ICC1026 | Iraq | 103 | ICC7185 | Turkey |
| 4 | ICC3093 | Iran | 54 | ICC1043 | India | 104 | ICC7200 | Egypt |
| 5 | ICC3631 | Iran | 55 | ICC1049 | India | 105 | ICC7235 | Lebanon |
| 6 | ICC6579 | Iran | 56 | ICC1052 | Pakistan | 106 | ICC7269 | Algeria |
| 7 | ICC6995 | Iran | 57 | ICC1059 | Iran | 107 | ICC7295 | Tunisia |
| 8 | ICC7167 | Turkey | 58 | ICC1069 | Russian federation | 108 | ICC7308 | Peru |
| 9 | ICC7305 | Afghanistan | 59 | ICC1070 | Russian federation | 109 | ICC7315 | Iran |
| 10 | BG 372 | IARI, New Delhi, India | 60 | ICC1092 | Iran | 110 | ICC7479 | India |
| 11 | ICC13185 | Iran | 61 | ICC 1093 | Iran | 111 | ICC7737 | Turkey |
| 12 | ICC14002 | Ethiopia | 62 | ICC 1098 | Iran | 112 | ICC7744 | Iran |
| 13 | ICC16069 | India | 63 | ICC 1118 | Iran | 113 | ICC7764 | Iran |
| 14 | ICC2 | India | 64 | ICC 1122 | Morocco | 114 | ICC7818 | Iran |
| 15 | ICC42 | India | 65 | ICC 1124 | Israel | 115 | ICC8265 | Turkey |
| 16 | ICC111 | India | 66 | ICC 1127 | India | 116 | ICC8718 | Afghanistan |
| 17 | ICC437 | India | 67 | ICC 1128 | India | 117 | ICC8752 | Afghanistan |
| 18 | ICC440 | India | 68 | ICC 1145 | India | 118 | ICC8759 | Afghanistan |
| 19 | ICC442 | India | 69 | ICC 1164 | Nigeria | 119 | BG 1053 | IARI, New Delhi, India |
| 20 | ICC448 | India | 70 | BG 372 |  | 120 | BG 372 | IARI, New Delhi, India |
| 21 | ICC482 | India | 71 | ICC 1180 | India | 121 | ICC8962 | Turkey |
| 22 | ICC506 | India | 72 | ICC 1181 | India | 122 | ICC9020 | Iran |
| 23 | ICC513 | India | 73 | ICC 1194 | India | 123 | ICC9049 | Iran |
| 24 | ICC515 | India | 74 | ICC 1220 | India | 124 | ICC9843 | Afghanistan |
| 25 | ICC531 | India | 75 | ICC 1228 | India | 125 | ICC10039 | Pakistan |
| 26 | ICC549 | India | 76 | ICC 1272 | Pakistan | 126 | ICC10199 | India |
| 27 | ICC562 | India | 77 | BG 3022 |  | 127 | ICC10232 | India |
| 28 | ICC571 | India | 78 | ICC1443 | India | 128 | ICC10258 | India |
| 29 | ICC591 | India | 79 | ICC2127 | Mexico | 129 | ICC10302 | Colombia |
| 30 | ICC619 | India | 80 | ICC2198 | India | 130 | ICC10314 | Turkey |
| 31 | ICC643 | India | 81 | ICC2202 | India | 131 | ICC10673 | Turkey |
| 32 | ICC668 | India | 82 | ICC2270 | Iran | 132 | ICC10685 | Turkey |
| 33 | ICC672 | India | 83 | ICC2277 | Iran | 133 | ICC11854 | Chile |
| 34 | ICC701 | India | 84 | ICC2342 | Iran | 134 | ICC12159 | Nepal |
| 35 | ICC729 | India | 85 | ICC2496 | Iran | 135 | ICC13077 | India |
| 36 | ICC738 | India | 86 | ICC2799 | Iran | 136 | ICC13085 | India |
| 37 | ICC752 | India | 87 | ICC3873 | Iran | 137 | ICC13590 | India |
| 38 | ICC839 | India | 88 | ICC3986 | Iran | 138 | ICC13696 | Iran |
| 39 | BG3022 | IARI, New Delhi, India | 89 | ICC4233 | Iran | 139 | ICC14098 | Ethiopia |
| 40 | ICC932 | India | 90 | ICC4234 | Iran | 140 | ICC14718 | India |
| 41 | ICC952 | India | 91 | ICC4254 | Iran | 141 | ICC14964 | India |
| 42 | ICC961 | India | 92 | ICC5047 | India | 142 | ICC15618 | India |
| 43 | ICC963 | India | 93 | ICC5119 | Israel | 143 | ICC16071 | India |
| 44 | ICC968 | India | 94 | ICC5123 | Israel | 144 | ICC16405 | Pakistan |
| 45 | ICC978 | India | 95 | ICC5135 | India | 145 | ICC 398 | India |
| 46 | ICC979 | Myanmar | 96 | ICC5178 | India | 146 | BG-1053 | IARI, New Delhi, India |
| 47 | ICC981 | Myanmar | 97 | ICC5923 | India | 147 | ICC 1205 | India |
| 48 | ICC987 | USA | 98 | ICC6537 | Iran | 148 | ICC2490 | India |
| 49 | ICC991 | Mexico | 99 | ICC7114 | Portugal | 149 | ICC7819 | Iran |
| 50 | ICC1000 | Mexico | 100 | BGM 547 | IARI, New Delhi, India | 150 | BG 1053 | IARI, New Delhi, India |
| 151 | ICC1852 | India | 201 | BG 372 | IARI, New Delhi, India | 251 | ICC12654 | Ethiopia |
| 152 | ICC1891 | India | 202 | ICC5752 | Bangladesh | 252 | ICC12847 | Ethiopia |
| 153 | ICC1896 | India | 203 | ICC6448 | Iran | 253 | ICC12959 | Unknown |
| 154 | ICC2083 | Mexico | 204 | ICC6608 | Iran | 254 | ICC13380 | Iran |
| 155 | ICC3696 | Iran | 205 | ICC6793 | Iran | 255 | ICC13450 | Iran |
| 156 | ICC4638 | India | 206 | ICC6802 | Iran | 256 | ICC13584 | India |
| 157 | ICC6661 | Iran | 207 | ICC6804 | Iran | 257 | ICC13593 | India |
| 158 | ICC9002 | Iran | 208 | ICC7560 | USA | 258 | ICC13712 | Iran |
| 159 | ICC9085 | Iran | 209 | ICC7949 | Iran | 259 | ICC13912 | Ethiopia |
| 160 | ICC9137 | Iran | 210 | ICC8052 | Iran | 260 | ICC14177 | Germany |
| 161 | BGM 547 | IARI, New Delhi, India | 211 | ICC8142 | Iran | 261 | ICC14220 | Kenya |
| 162 | ICC9499 | India | 212 | ICC8186 | Afghanistan | 262 | ICC14230 | India |
| 163 | ICC9954 | India | 213 | ICC8259 | Ethiopia | 263 | BG 372 | IARI, New Delhi, India |
| 164 | ICC10302 | Colombia | 214 | ICC8287 | India | 264 | ICC14316 | India |
| 165 | ICC10961 | India | 215 | ICC8314 | India | 265 | ICC14356 | India |
| 166 | ICC12422 | India | 216 | ICC9032 | Iran | 266 | BG 3022 | IARI, New Delhi, India |
| 167 | ICC13719 | Iran | 217 | ICC9175 | Iran | 267 | ICC14423 | India |
| 168 | BG 3022 | IARI, New Delhi, India | 218 | ICC9242 | Hungary | 268 | ICC14436 | India |
| 169 | ICC15844 | Syria | 219 | ICC9362 | Iran | 269 | ICC14452 | Iran |
| 170 | ICC16841 | India | 220 | ICC9510 | India | 270 | ICC14462 | India |
| 171 | BG 547 | IARI, New Delhi, India | 221 | ICC9636 | Afghanistan | 271 | ICC14469 | Bangladesh |
| 172 | ICC1594 | India | 222 | ICC9643 | Afghanistan | 272 | ICC14472 | Bangladesh |
| 173 | ICC1609 | India | 223 | ICC9676 | Afghanistan | 273 | ICC14484 | Bangladesh |
| 174 | ICC1836 | India | 224 | ICC9978 | India | 274 | ICC14489 | Bangladesh |
| 175 | ICC1837 | India | 225 | ICC9984 | India | 275 | ICC14490 | Bangladesh |
| 176 | ICC1867 | India | 226 | ICC10539 | India | 276 | ICC14515 | Bangladesh |
| 177 | ICC1950 | India | 227 | ICC10561 | India | 277 | ICC14564 | Bangladesh |
| 178 | ICC2072 | India | 228 | ICC10778 | Turkey | 278 | ICC14566 | Bangladesh |
| 179 | ICC2211 | India | 229 | ICC10829 | India | 279 | ICC14787 | India |
| 180 | ICC2220 | Mexico | 230 | ICC10947 | India | 280 | ICC14881 | Russian Federation |
| 181 | ICC2223 | India | 231 | ICC11007 | India | 281 | ICC15014 | India |
| 182 | ICC2514 | Iran | 232 | BG 3022 | IARI, New Delhi, India | 282 | ICC15061 | India |
| 183 | ICC2577 | Iran | 233 | ICC11091 | India | 283 | ICC15103 | India |
| 184 | ICC2698 | Iran | 234 | ICC11180 | India | 284 | ICC15186 | India |
| 185 | ICC2938 | Iran | 235 | ICC11201 | India | 285 | ICC15452 | Morocco |
| 186 | ICC3410 | Iran | 236 | ICC11316 | India | 286 | ICC15657 | India |
| 187 | ICC3571 | Pakistan | 237 | ICC11781 | Chile | 287 | ICC15717 | Syria |
| 188 | ICC3684 | Iran | 238 | ICC12291 | Nepal | 288 | ICC15823 | Syria |
| 189 | BG 547 | IARI, New Delhi, India | 239 | ICC12296 | Nepal | 289 | ICC15825 | Syria |
| 190 | ICC4573 | India | 240 | ICC12299 | Nepal | 290 | ICC15851 | India |
| 191 | ICC5022 | India | 241 | ICC12307 | Myanmar | 291 | BG 372 | IARI, New Delhi, India |
| 192 | ICC4055 | Iran | 242 | BGM 547 | IARI, New Delhi, India | 292 | BG 1053 | IARI, New Delhi, India |
| 193 | ICC5092 | Egypt | 243 | ICC12321 | Unknown | 293 | ICC16569 | Pakistan |
| 194 | ICC5122 | Israel | 244 | ICC12337 | India | 294 | ICC16853 | India |
| 195 | ICC5383 | India | 245 | ICC12433 | India | 295 | ICC9628 | Afghanistan |
| 196 | ICC5449 | India | 246 | ICC12434 | India | 296 | ICC14569 | Bangladesh |
| 197 | ICC5472 | Mexico | 247 | ICC12458 | India | 297 | ICC11378 | India |
| 198 | ICC5581 | Mexico | 248 | ICC12461 | India | 298 | ICC14334 | India |
| 199 | ICC5697 | India | 249 | ICC12463 | India | 299 | ICC16579 | Pakistan |
| 200 | ICC5710 | India | 250 | ICC12470 | India | 300 | ICC9778 | Afghanistan |

Note : List of the entries mentioned above consisting of 300 entries including repeated checks
