# Supplementary material for: Genome-wide association mapping identifies novel SNPs for root nodulation and agronomic traits in chickpea
Source: Front Plant Sci. 2024 Oct 15;15:1395938. doi: 10.3389/fpls.2024.1395938 (PMC11518797; doi:10.3389/fpls.2024.1395938)
Supplement: Supplementary file 1 [file DataSheet1.zip › Supplementary figures 1-5.pdf]

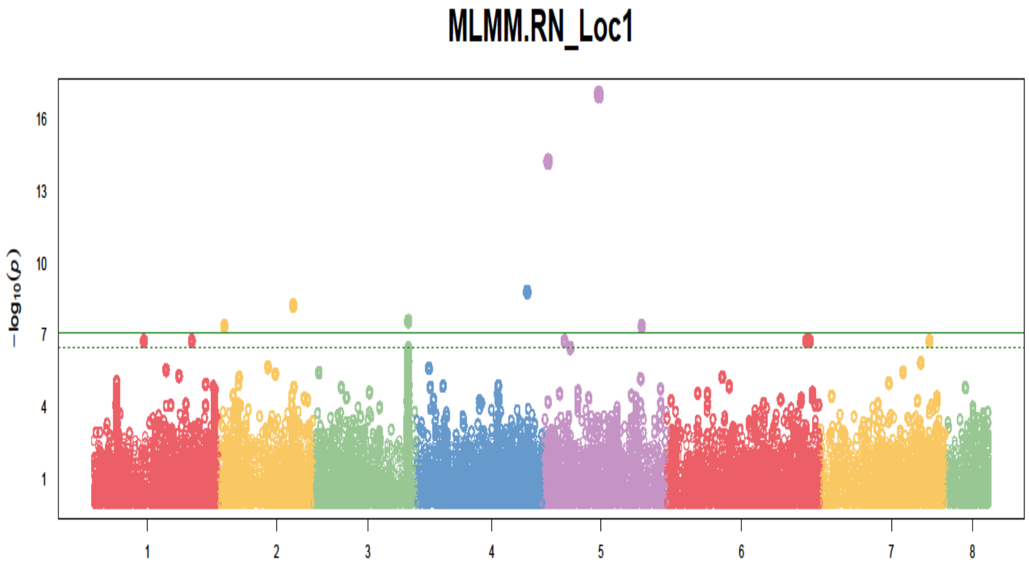

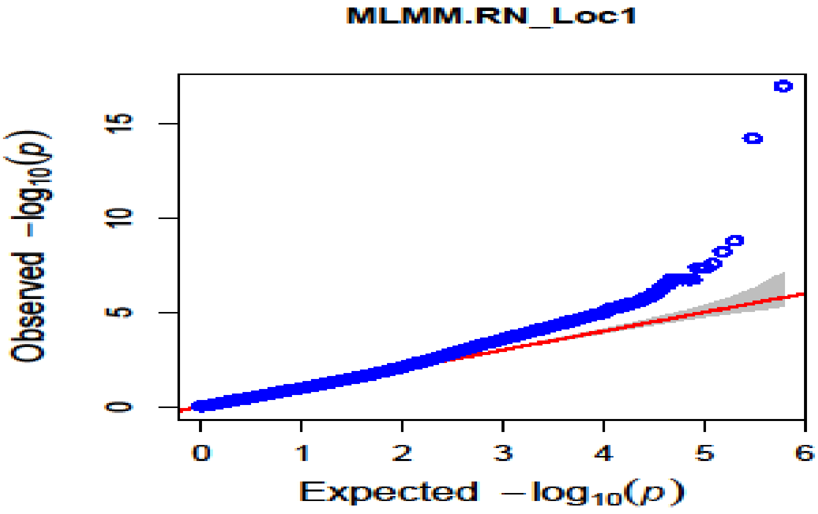

Supplementary figure1a: Manhattan plot illustrating SNPs linked to number of nodule along with their corresponding statistical significance represented by Q-Q plot for location 1-FarmCPU model

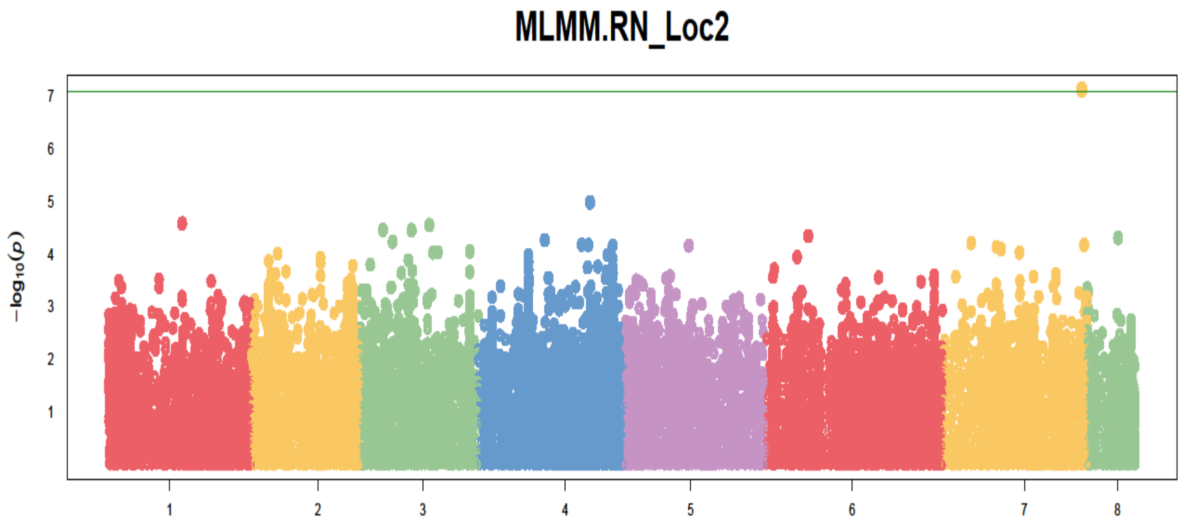

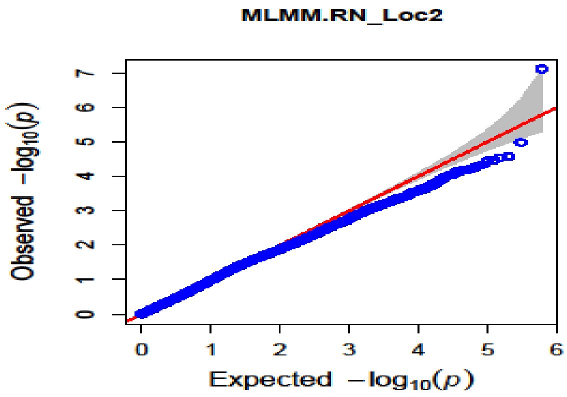

Supplementary figure1b: Manhattan plot illustrating SNPs linked to number of nodule along with their corresponding statistical significance represented by Q-Q plot for location 2-FarmCPU model

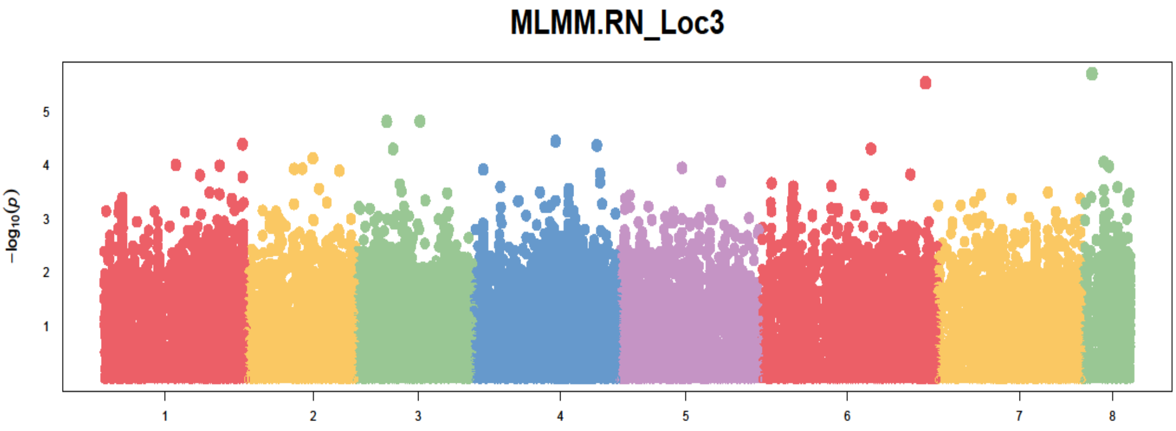

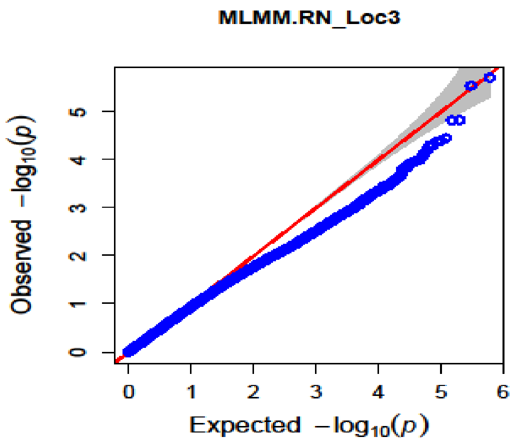

Supplementary figure1c: Manhattan plot illustrating SNPs linked to number of nodule along with their corresponding statistical significance represented by Q-Q plot for location 3-FarmCPU model

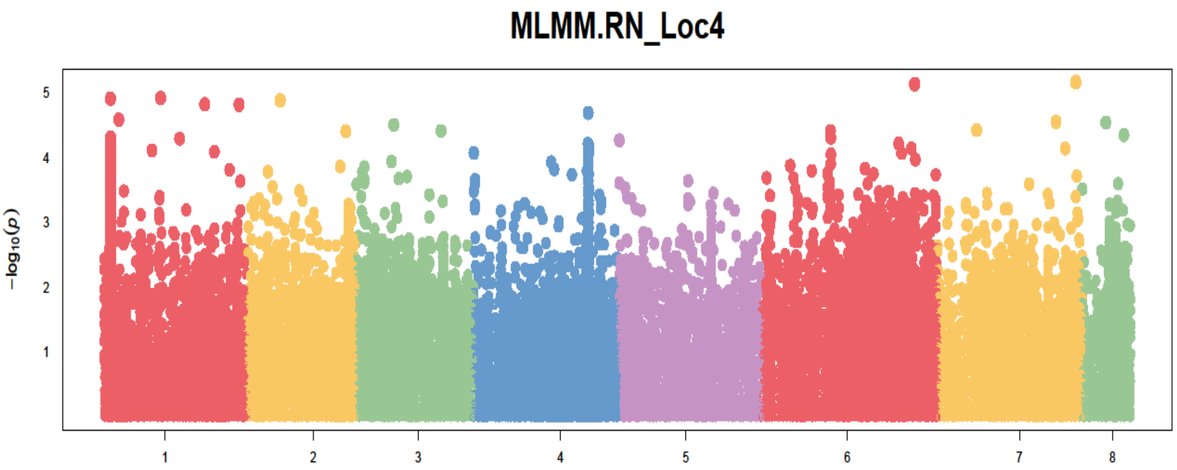

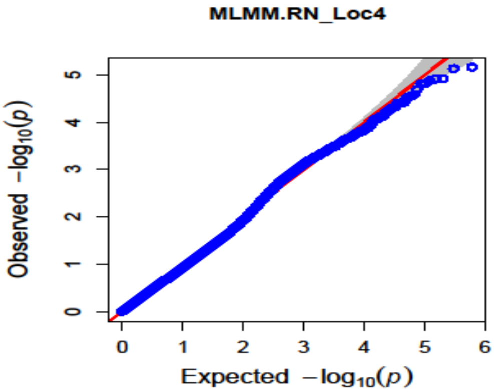

Supplementary figure1c: Manhattan plot illustrating SNPs linked to number of nodule along with their corresponding statistical significance represented by Q-Q plot for location 4-FarmCPU model

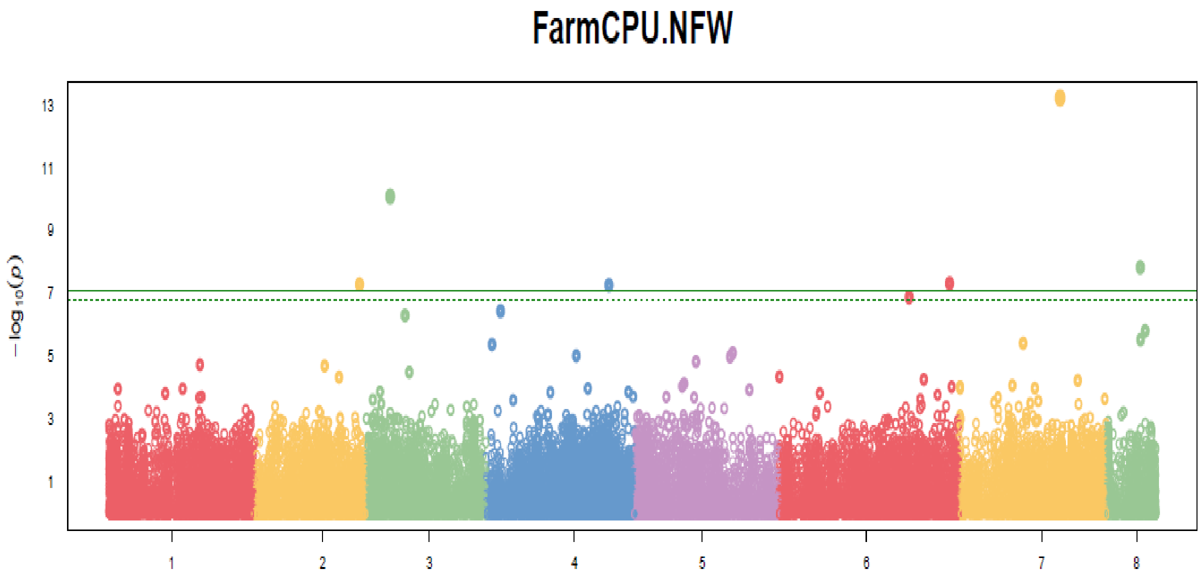

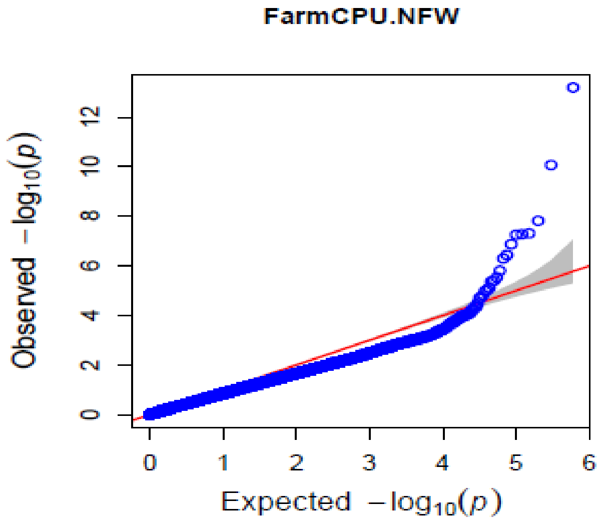

Supplementary figure2a: Manhattan plot illustrating SNPs linked to nodule fresh weight along with their corresponding statistical significance represented by Q-Q plot for location 1-FarmCPU model.

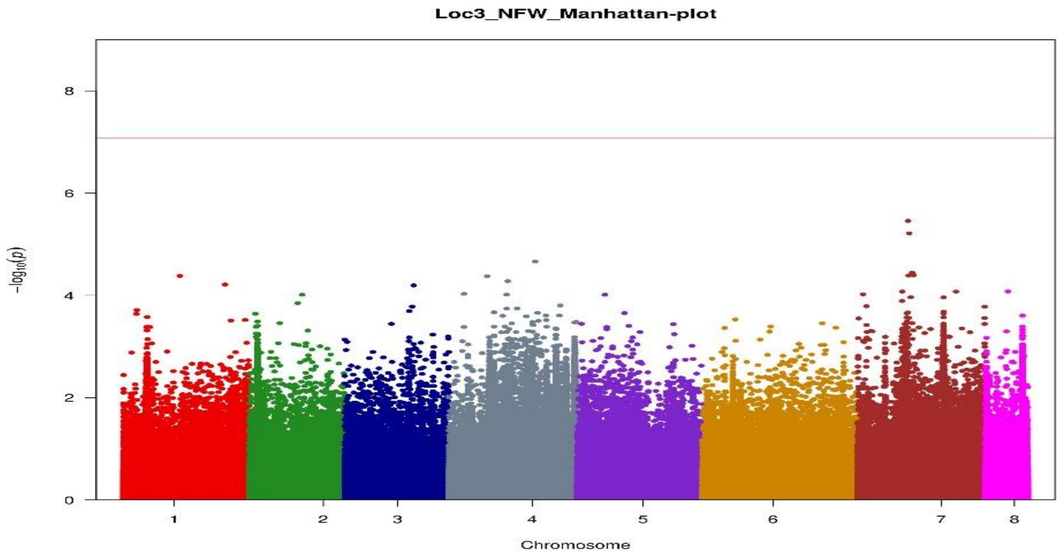

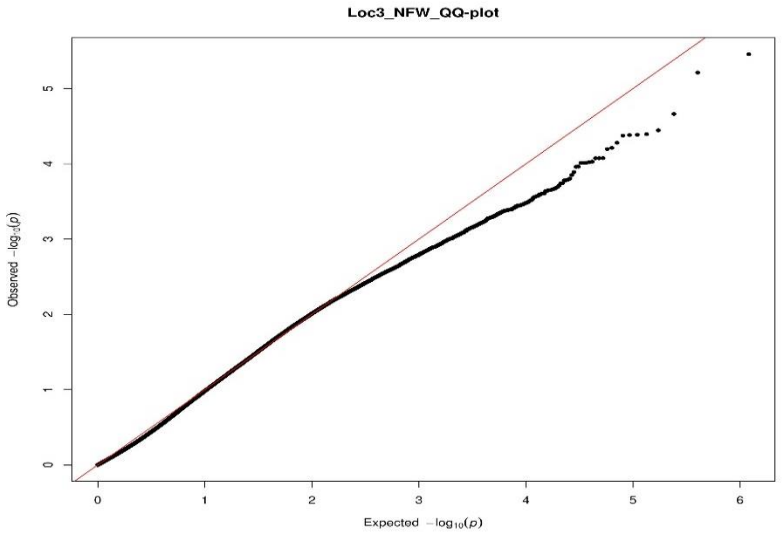

Supplementary figure2b: Manhattan plot illustrating SNPs linked to nodule fresh weight along with their corresponding statistical significance represented by Q-Q plot for location 3-FarmCPU model

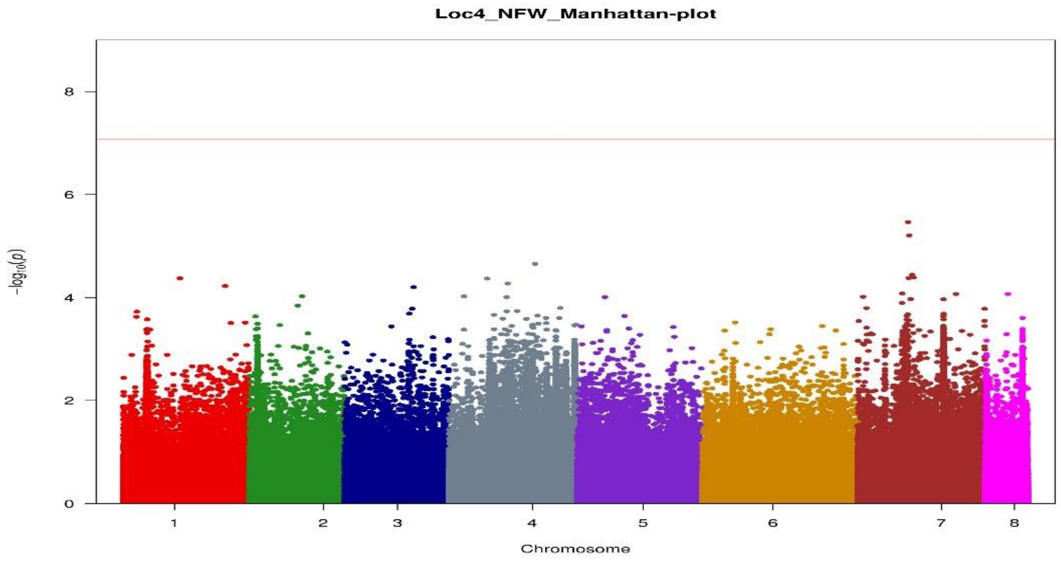

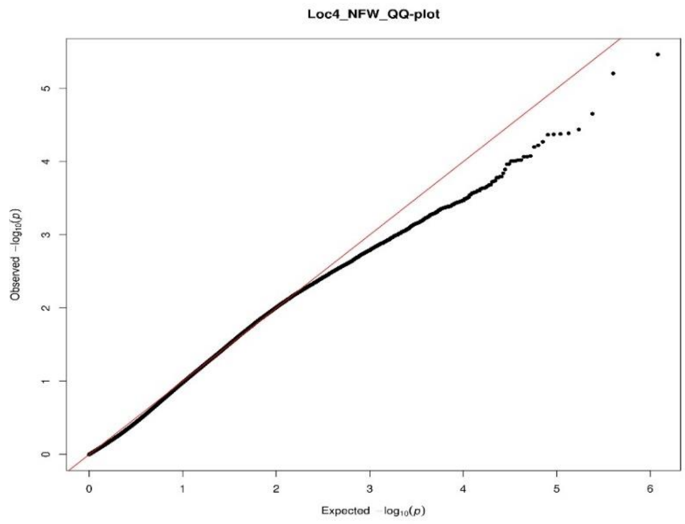

Supplementary figure2c: Manhattan plot illustrating SNPs linked to nodule fresh weight along with their corresponding statistical significance represented by Q-Q plot for location 4-FarmCPU model

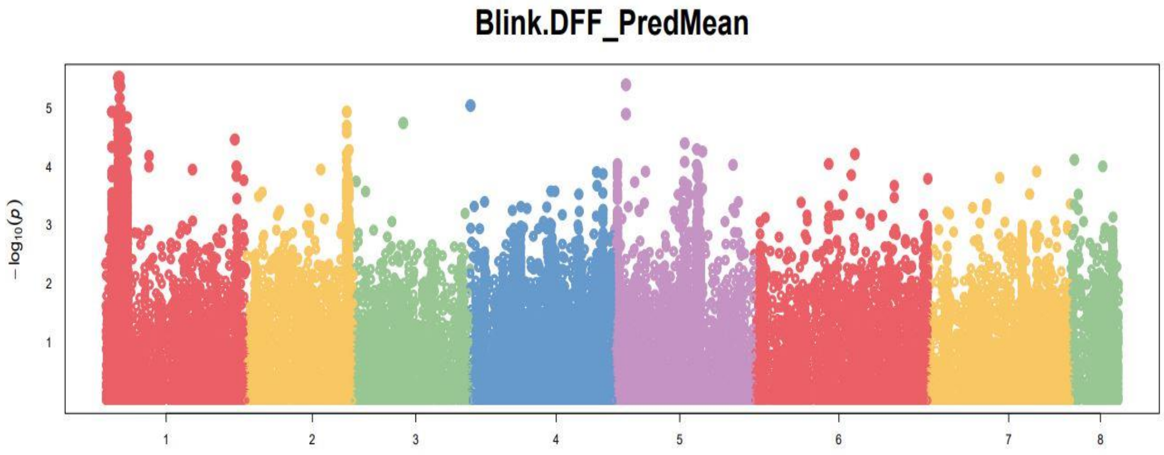

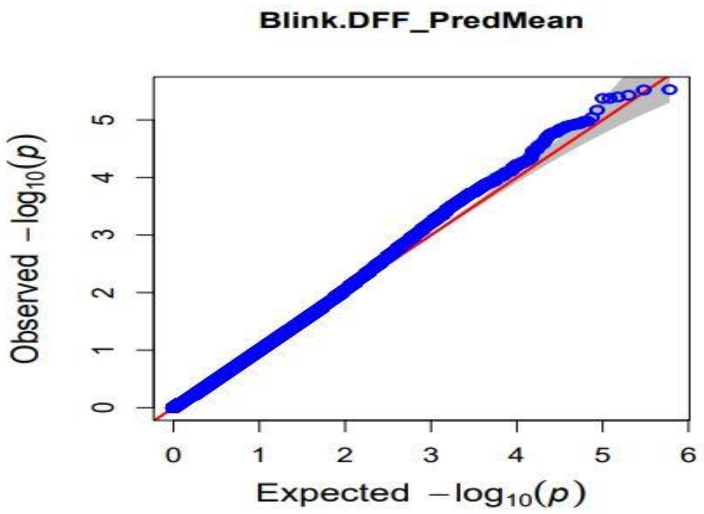

Supplementary Figure3a: Manhattan plot illustrating SNPs linked to Days fifty percentage flowering along with their corresponding statistical significance represented by Q-Q plot .

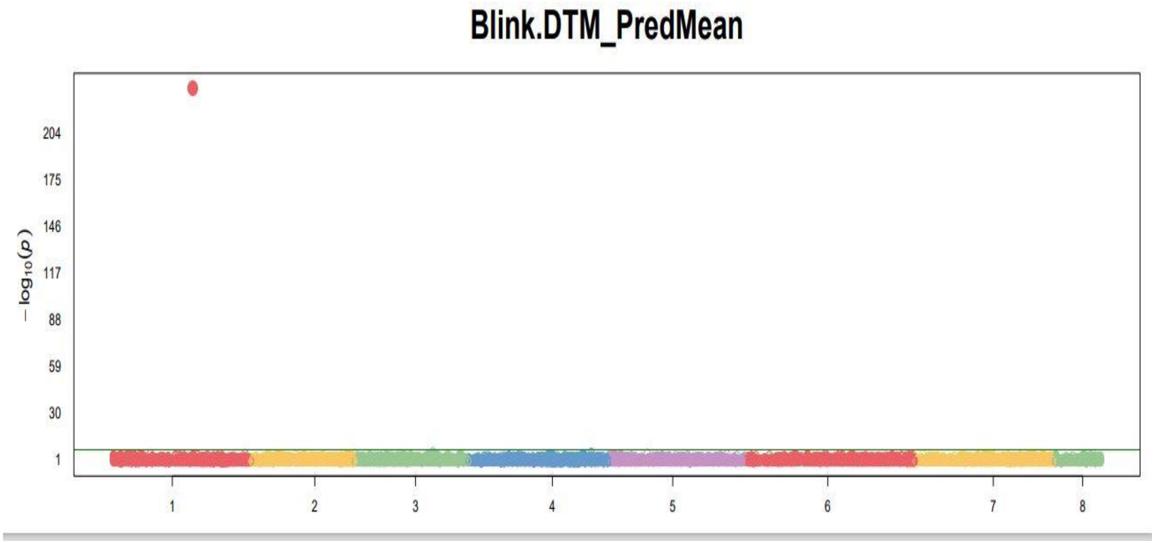

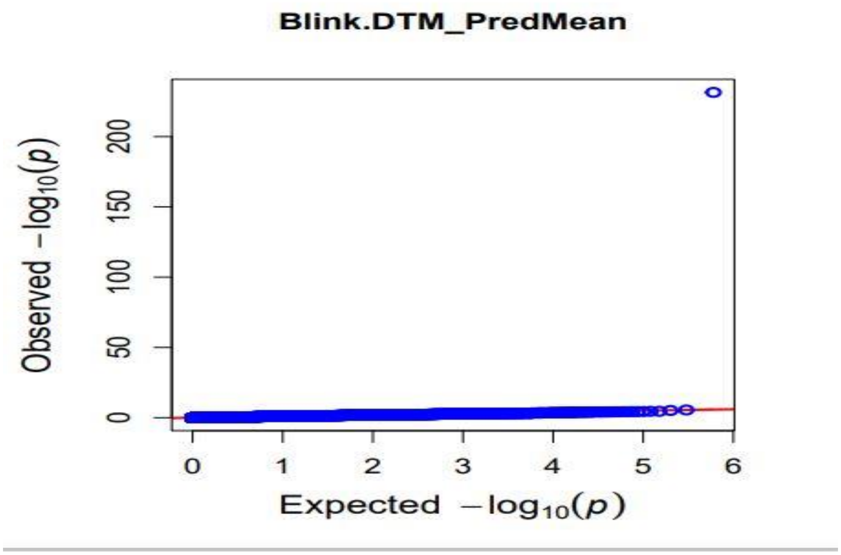

Supplementary Figure3b: Manhattan plot illustrating SNPs linked to Days to maturity along with their corresponding statistical significance represented by Q-Q plot

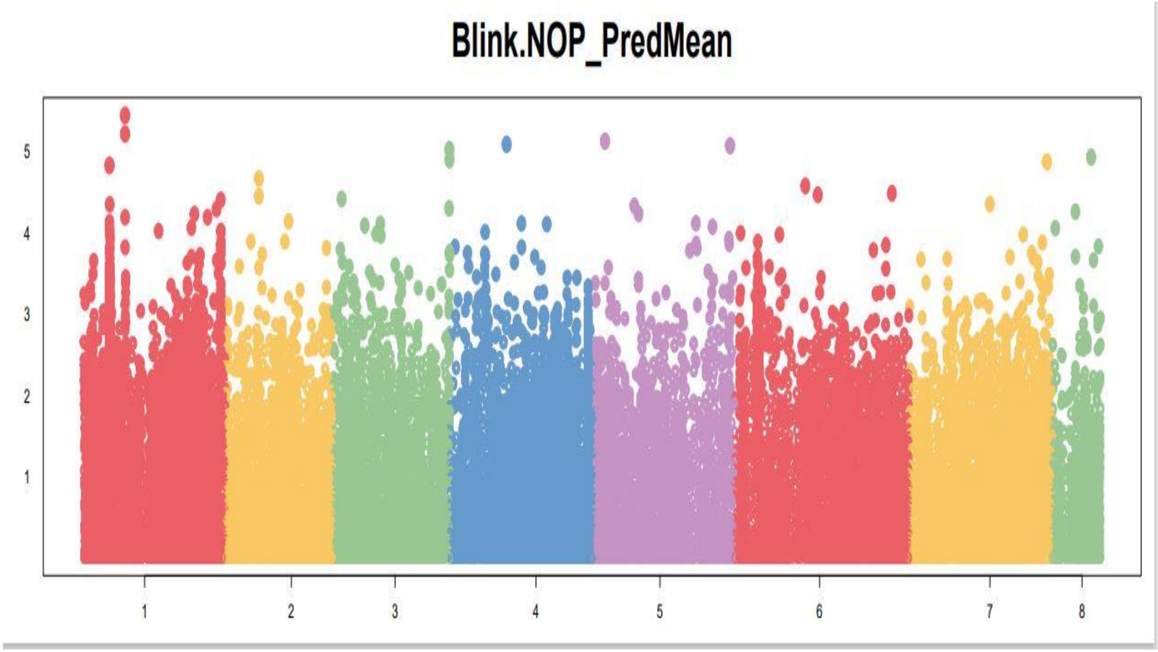

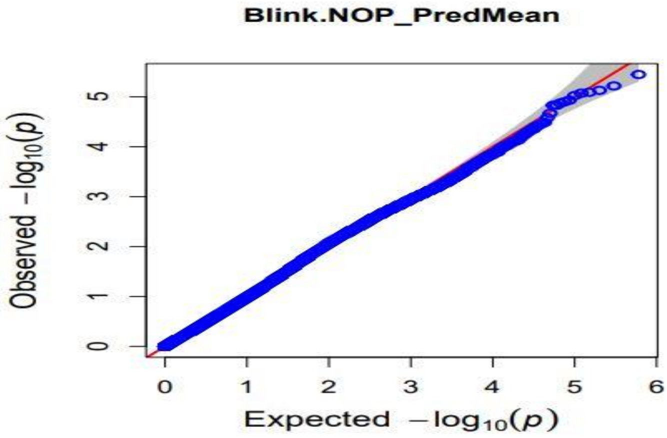

Supplementary Figure3c: Manhattan plot illustrating SNPs linked to number of pods along with their corresponding statistical significance represented by Q-Q plot

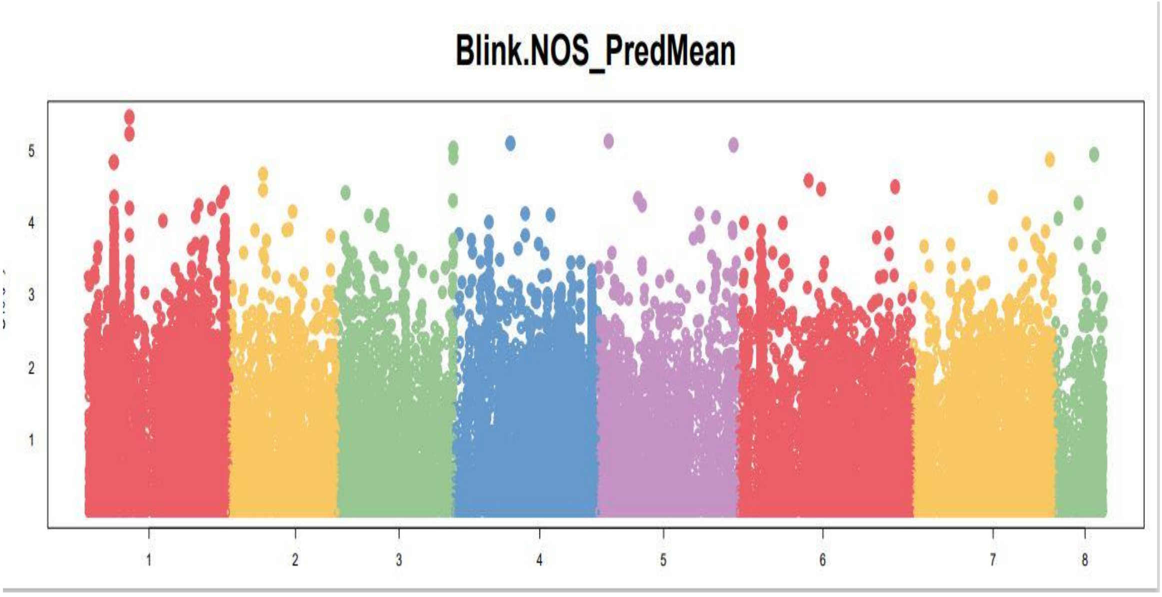

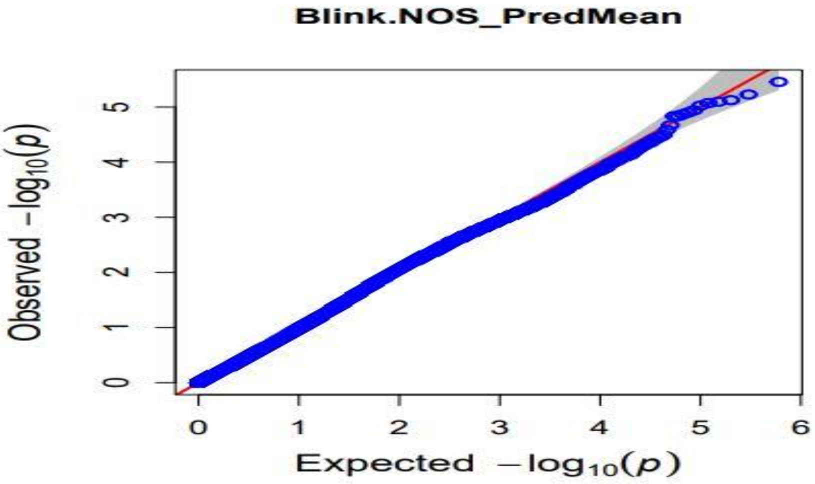

Supplementary Figure3d: Manhattan plot illustrating SNPs linked to number of seeds along with their corresponding statistical significance represented by Q-Q plot

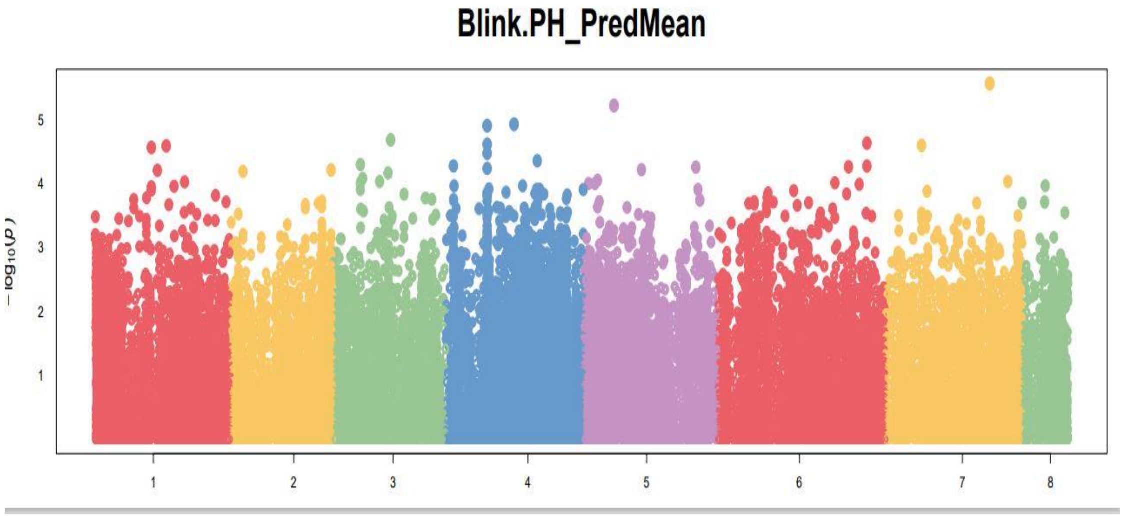

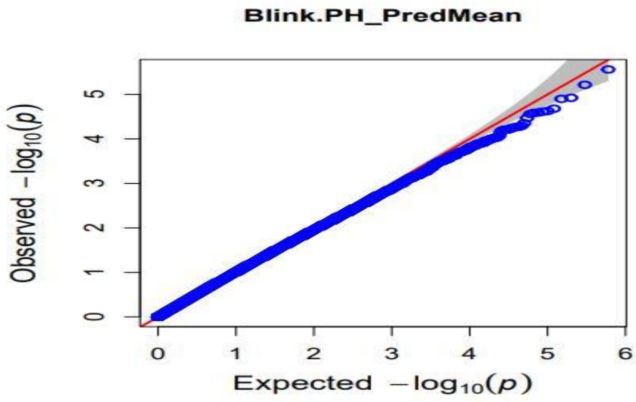

Supplementary Figure3e: Manhattan plot illustrating SNPs linked to plant height along with their corresponding statistical significance represented by Q-Q plot

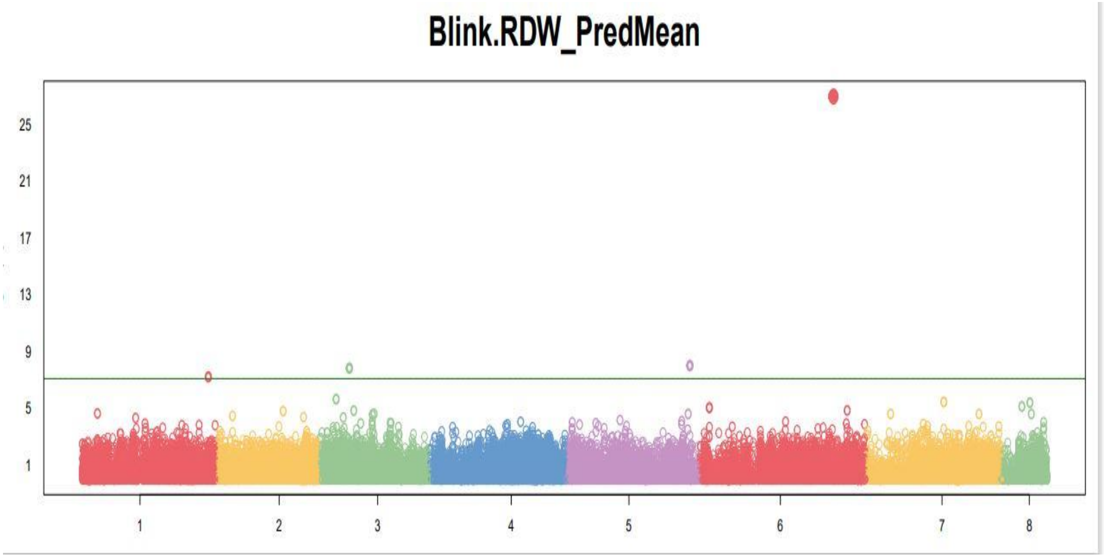

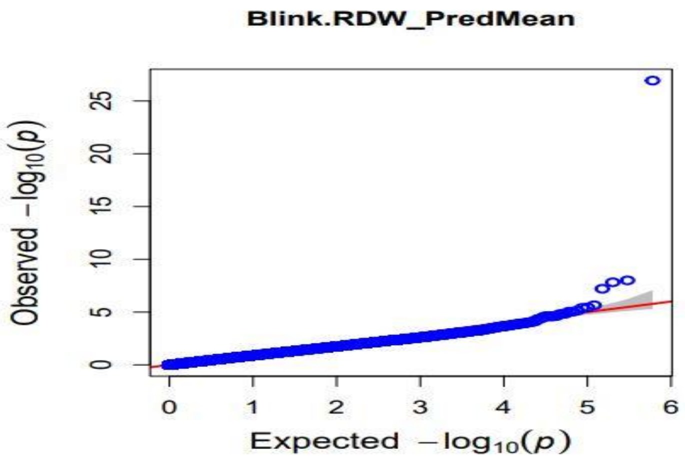

Supplementary Figure3f: Manhattan plot illustrating SNPs linked to root dry weight with their corresponding statistical significance represented by Q-Q plot

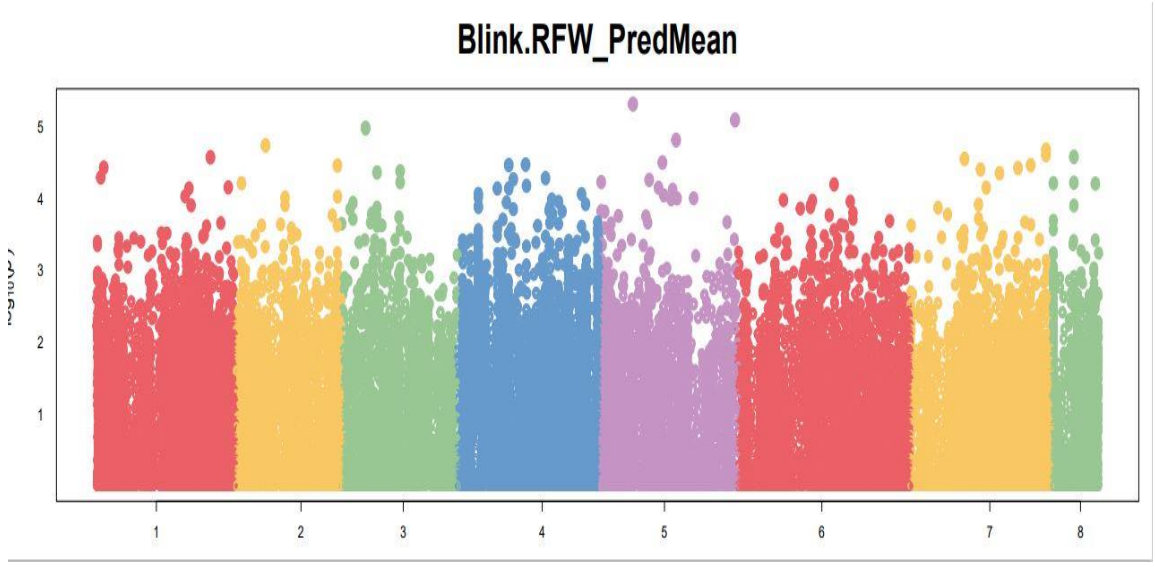

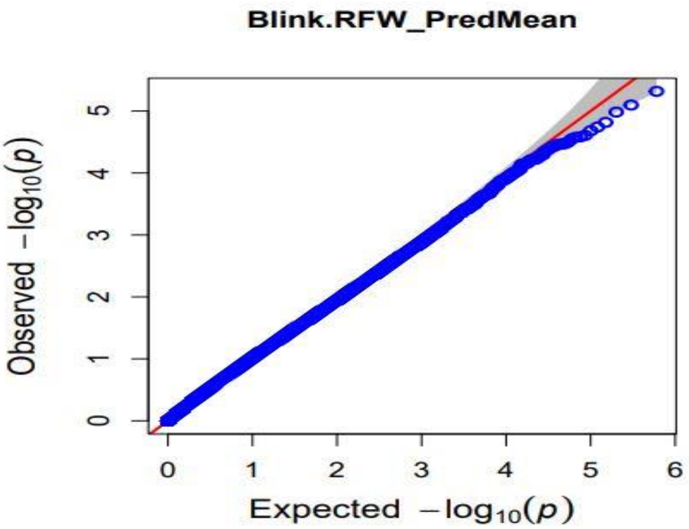

Supplementary Figure3g: Manhattan plot illustrating SNPs linked to root fresh weight

with their corresponding statistical significance represented by Q-Q plot

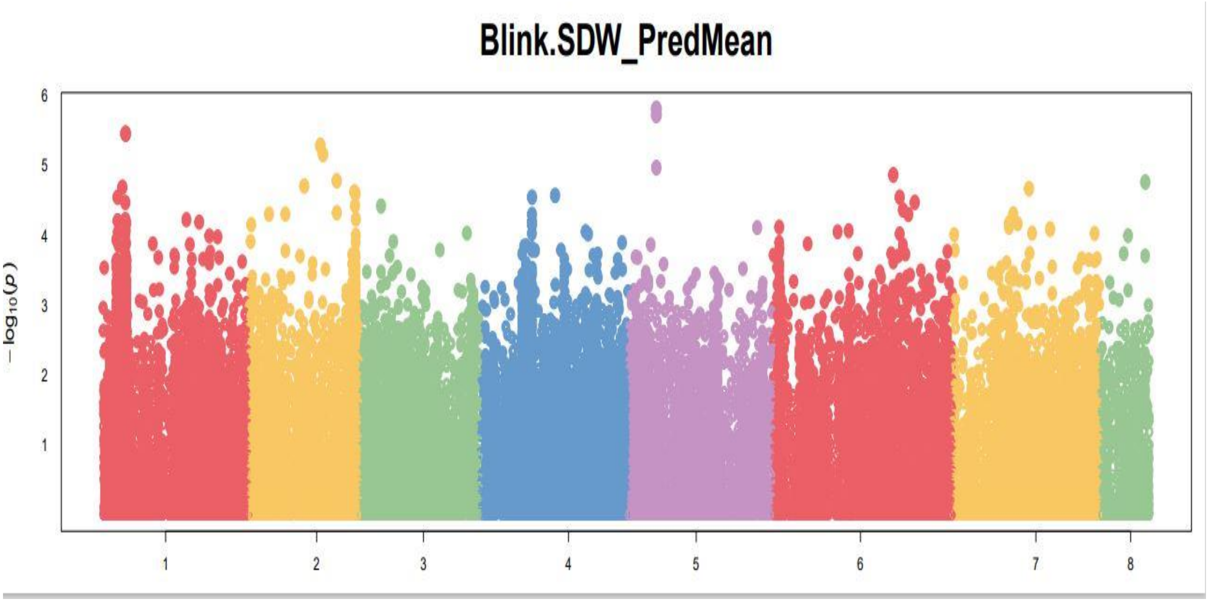

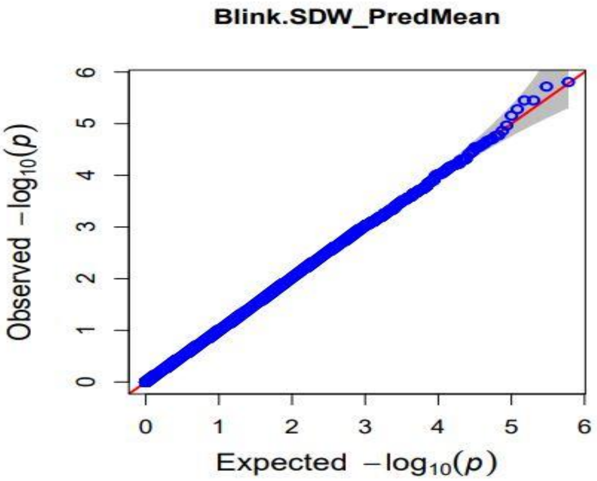

Supplementary Figure3h: Manhattan plot illustrating SNPs linked to r shoot dry weight with their corresponding statistical significance represented by Q-Q plot

H

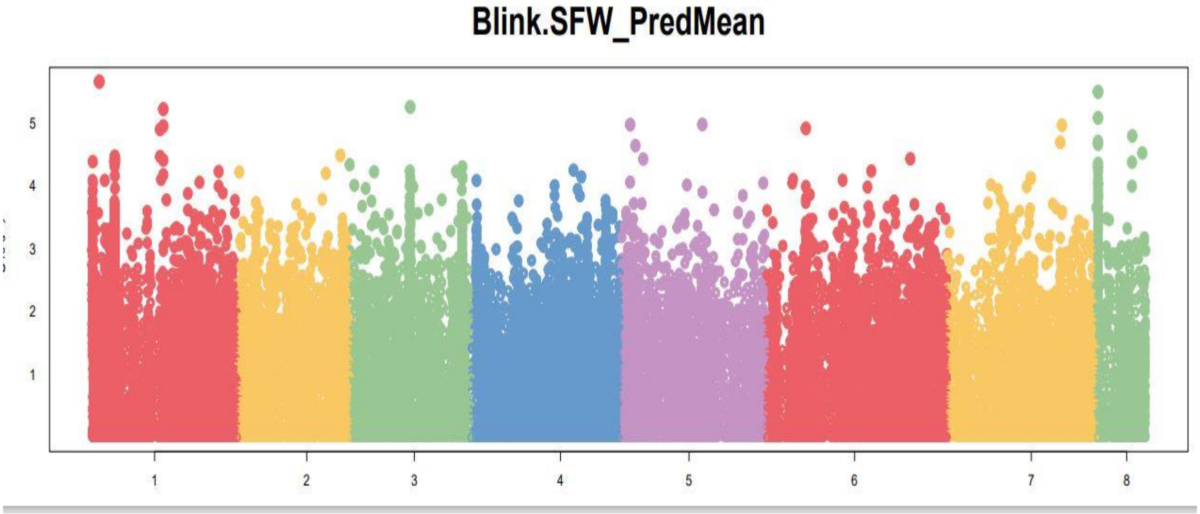

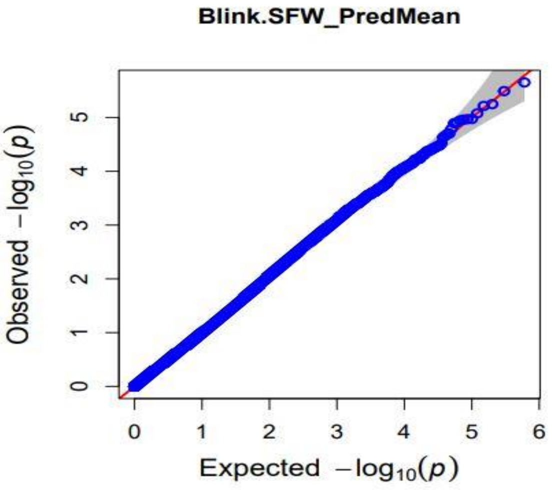

Supplementary Figure3i: Manhattan plot illustrating SNPs linked to shoot fresh weight with their corresponding statistical significance represented by Q-Q plot

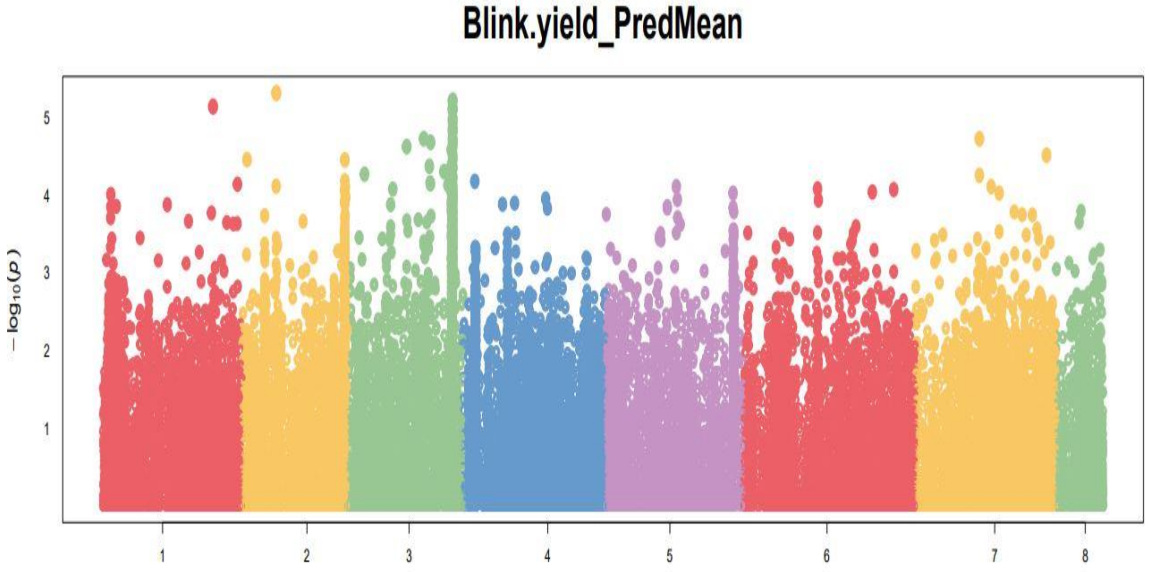

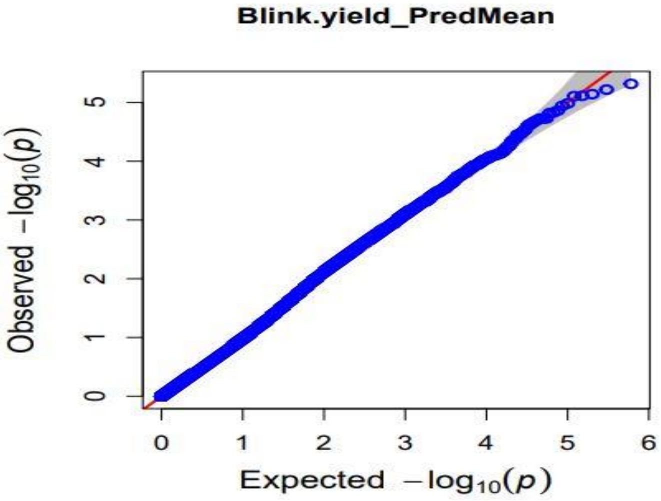

Supplementary Figure3j: Manhattan plot illustrating SNPs linked to yield with their corresponding statistical significance represented by Q-Q plot

Supplementary Figure3: Manhattan plot illustrating SNPs linked to specific chickpea traits, along with their corresponding statistical significance represented by Q-Q plot for location 1 identified from Blink model.

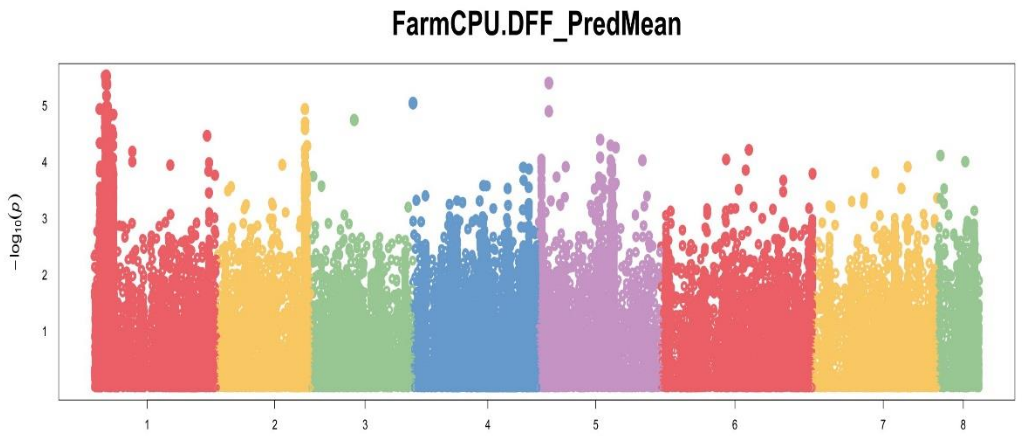

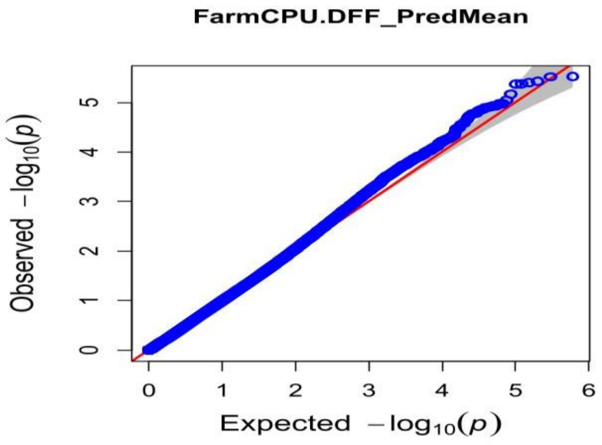

Supplementary Figure4a: Manhattan plot illustrating SNPs linked to Days fifty percentage

flowering along with their corresponding statistical significance represented by Q-Q plot.

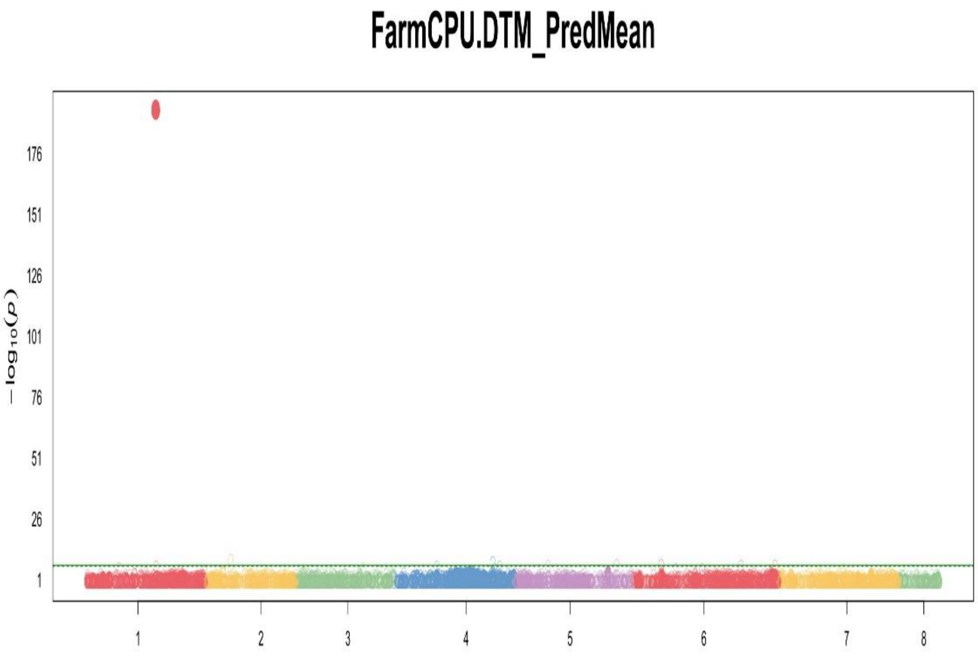

FarmCPU.DTM_PredMean
176
151
126
101
76
51
26
1
$-\log_{10}(p)$
1
2
3
4
5
6
7
8

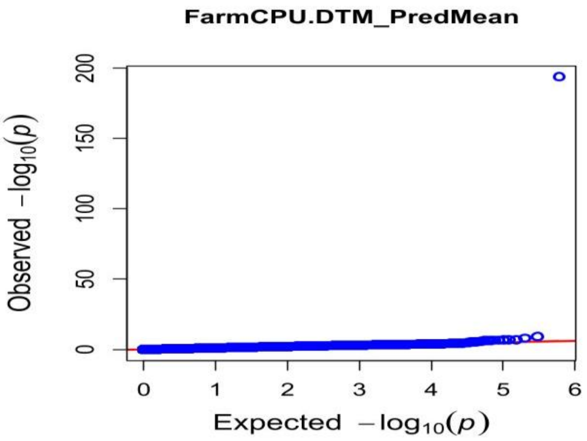

Supplementary Figure4b: Manhattan plot illustrating SNPs linked to Days to maturity along with their corresponding statistical significance represented by Q-Q plot

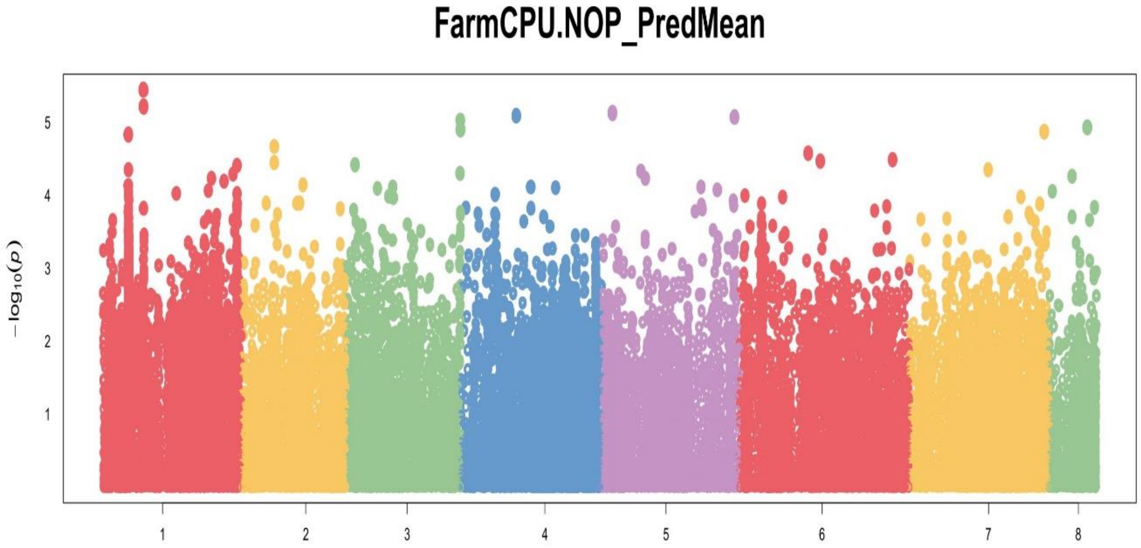

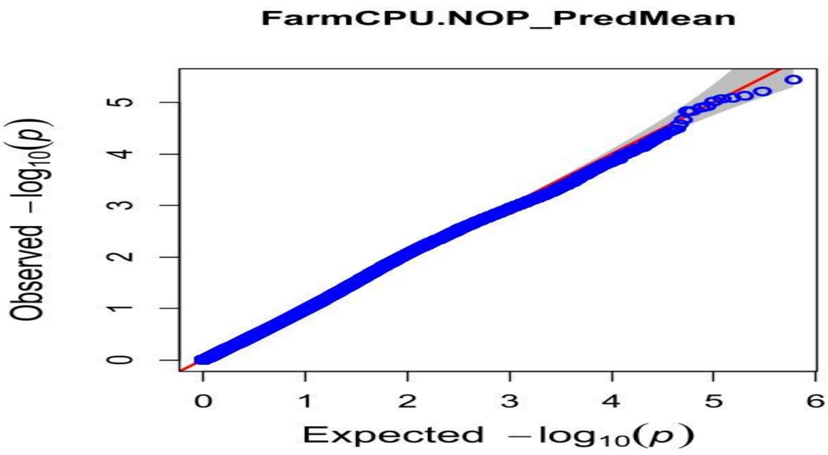

Supplementary Figure4c: Manhattan plot illustrating SNPs linked to number pods of along with their corresponding statistical significance represented by Q-Q plot

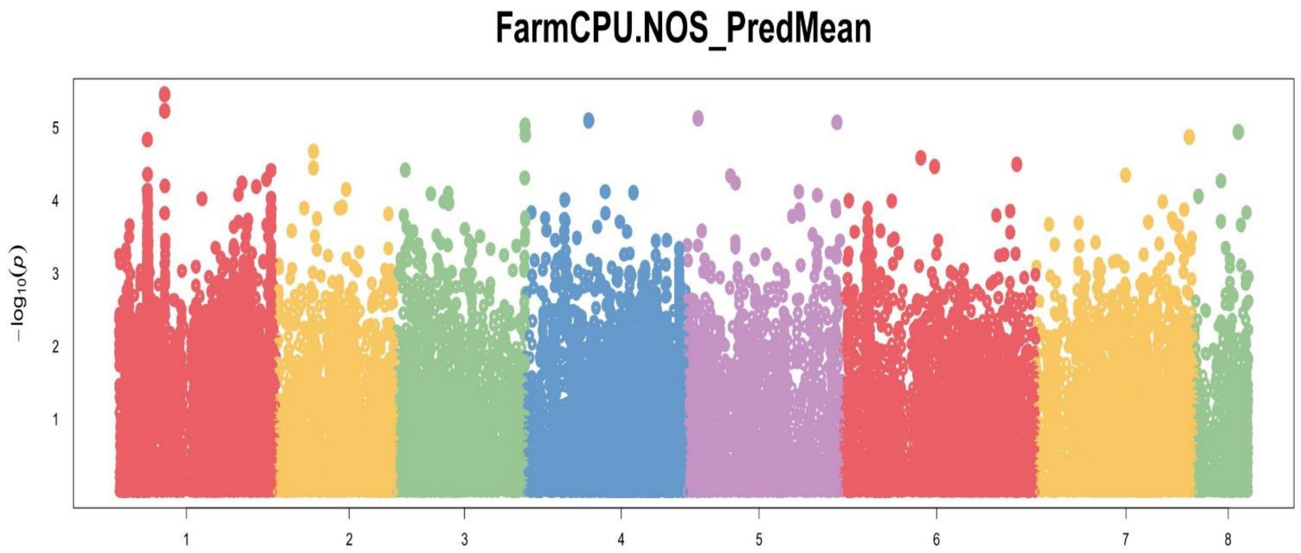

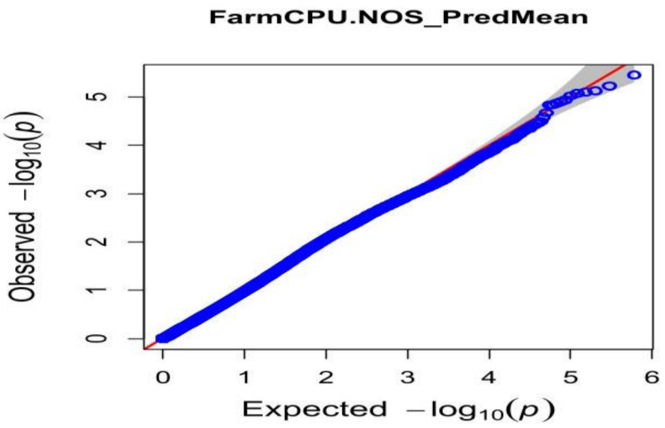

Supplementary Figure4d: Manhattan plot illustrating SNPs linked to number of seeds along with their corresponding statistical significance represented by Q-Q plot

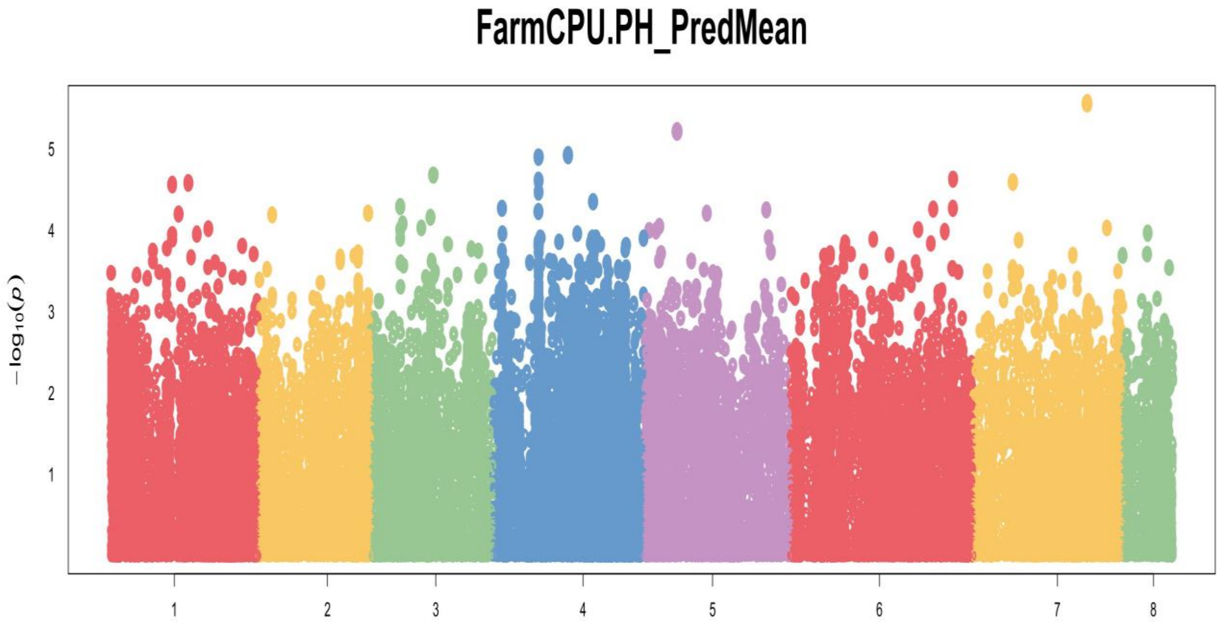

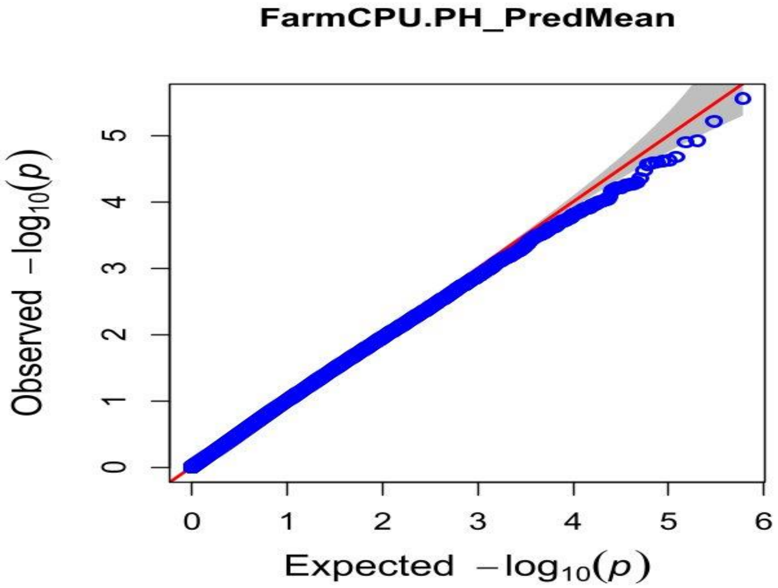

Supplementary Figure4e: Manhattan plot illustrating SNPs linked to plant height along with their corresponding statistical significance represented by Q-Q plot

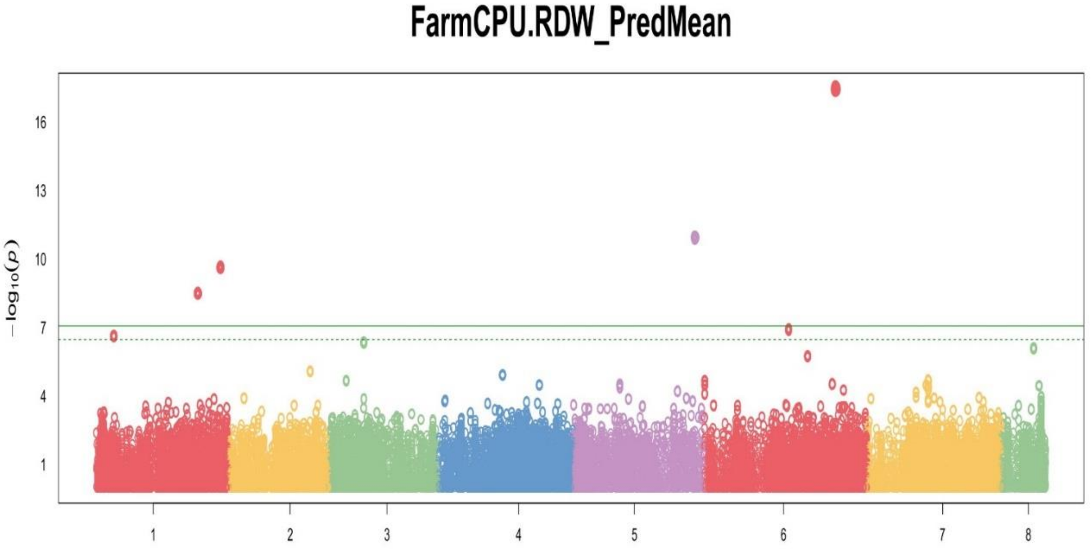

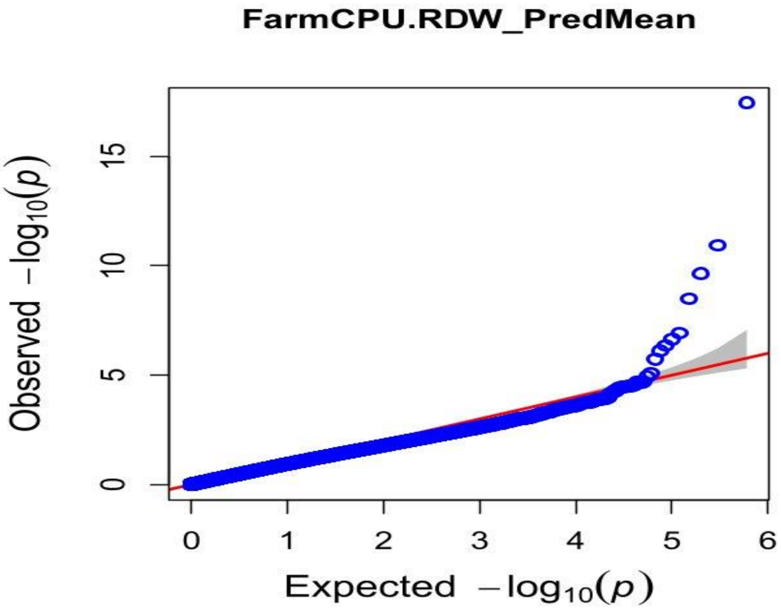

Supplementary Figure4f: Manhattan plot illustrating SNPs linked to root dry weight with their corresponding statistical significance represented by Q-Q plot

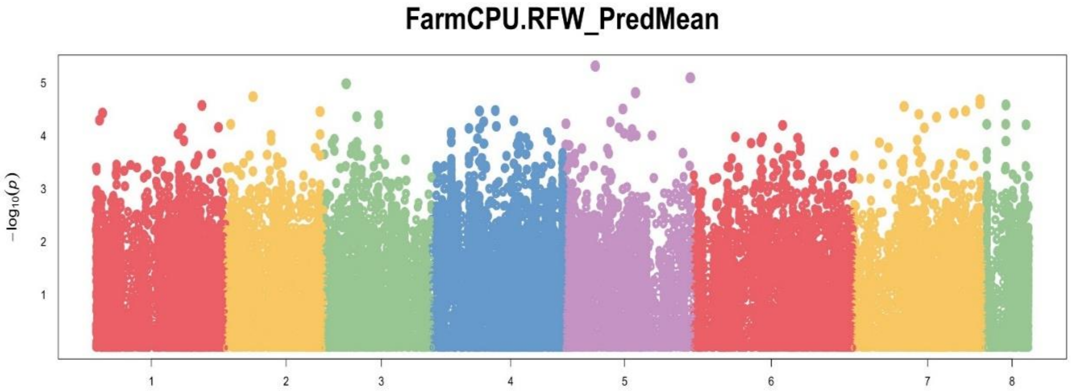

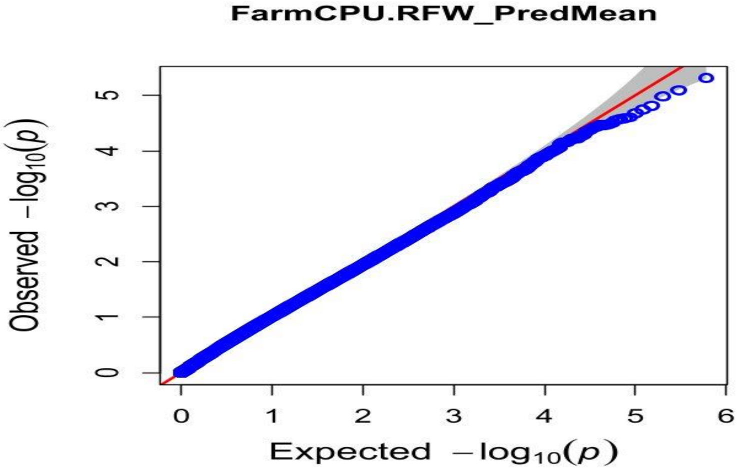

Supplementary Figure4g: Manhattan plot illustrating SNPs linked to root fresh weight with

their corresponding statistical significance represented by Q-Q plot

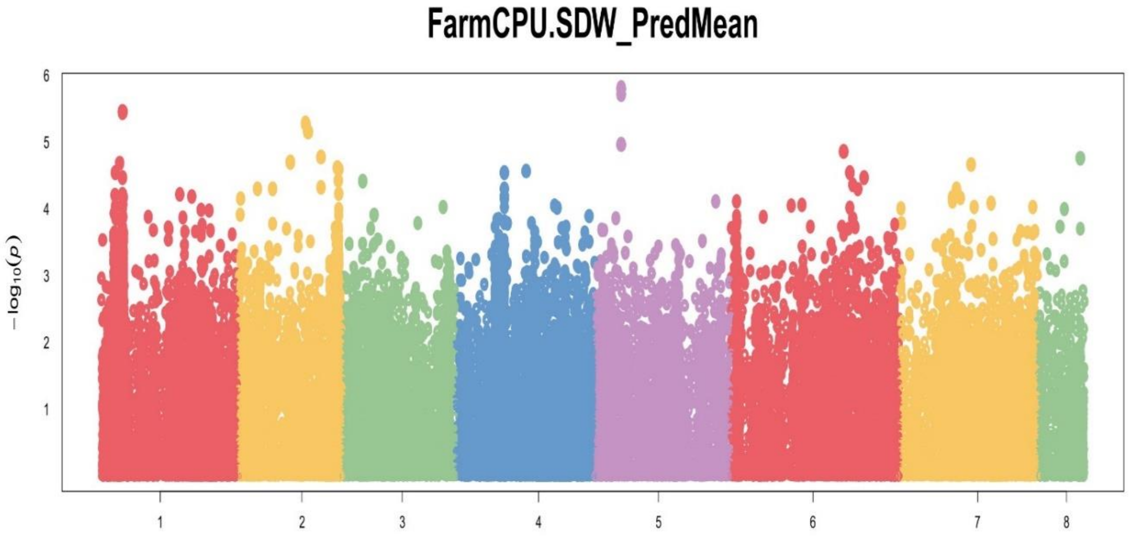

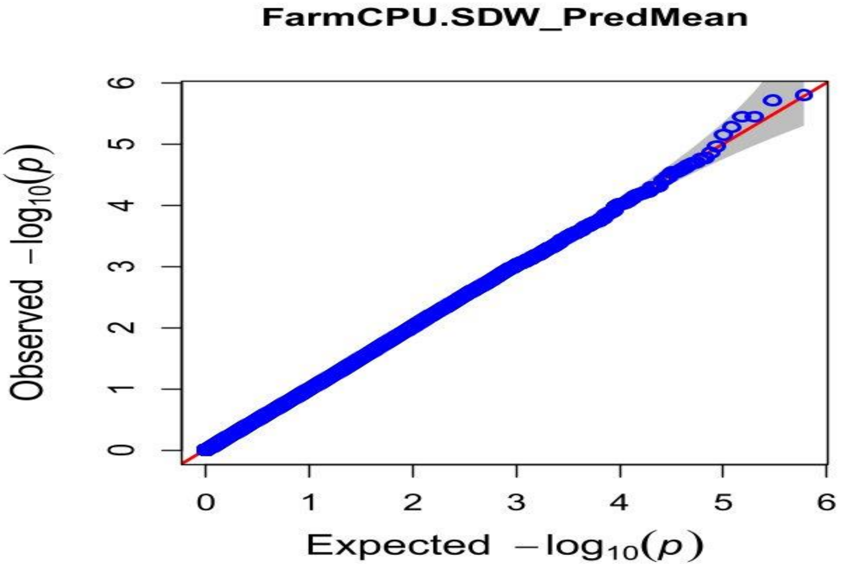

Supplementary Figure4h: Manhattan plot illustrating SNPs linked to shoot dry weight with their corresponding statistical significance represented by Q-Q plot

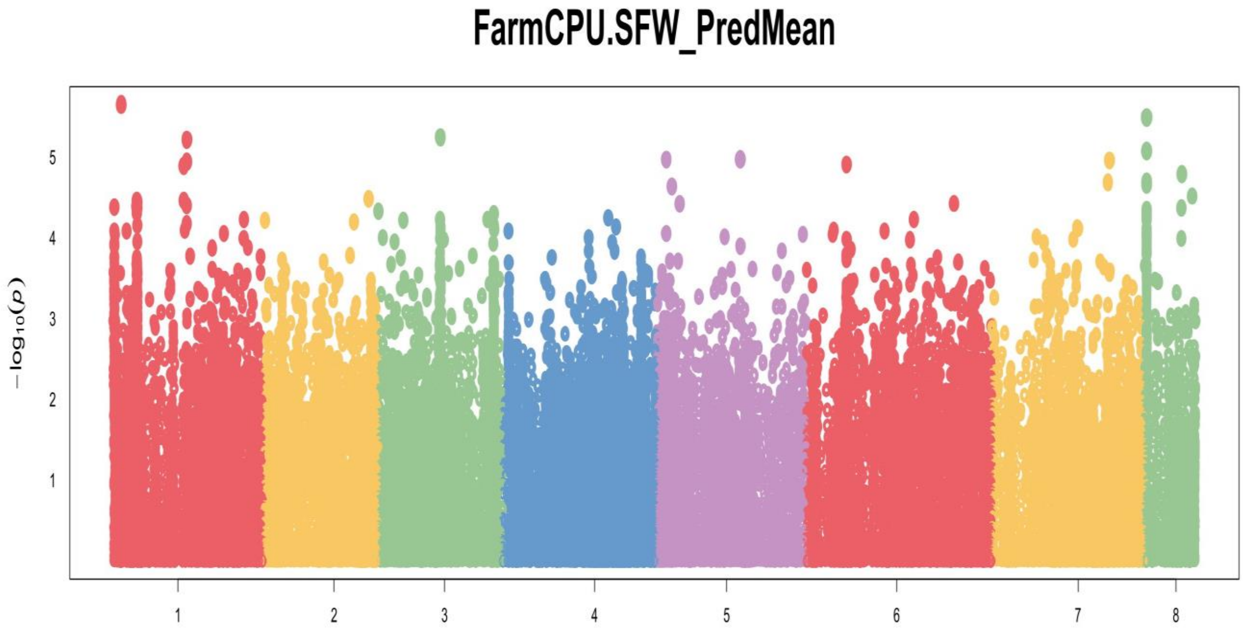

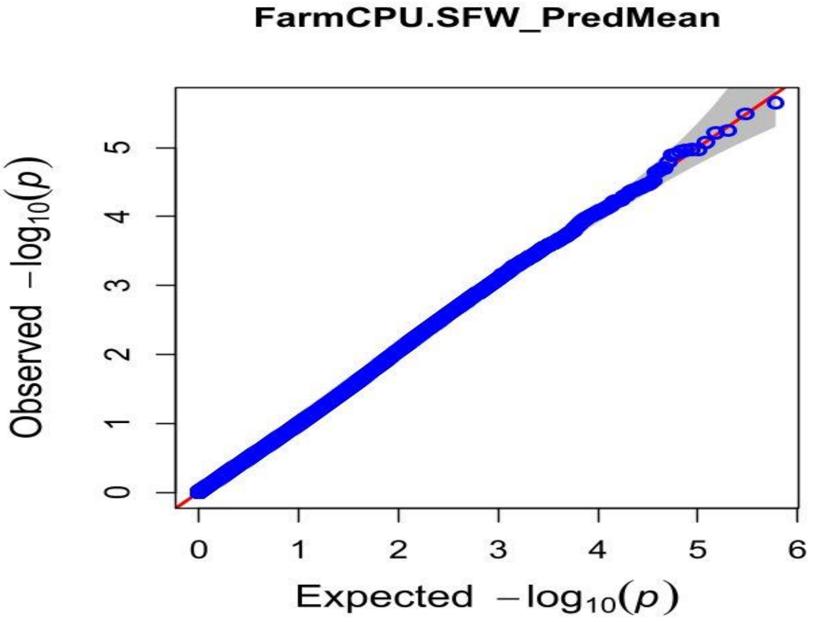

Supplementary Figure4i: Manhattan plot illustrating SNPs linked to shoot fresh weight with their corresponding statistical significance represented by Q-Q plot

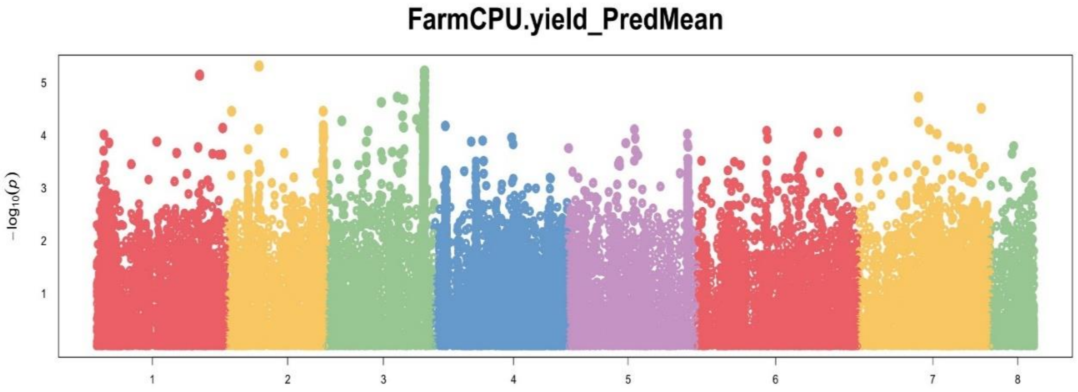

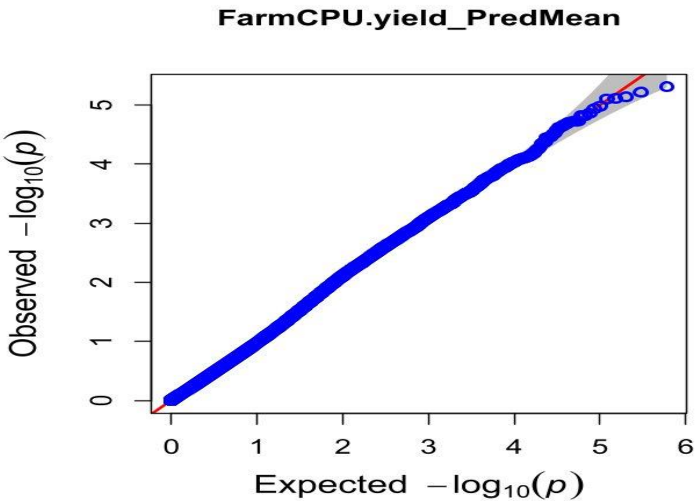

Supplementary Figure4j: Manhattan plot illustrating SNPs linked to yield with their corresponding statistical significance represented by Q-Q plot

Supplementary Figure4: Manhattan plot illustrating SNPs linked to specific chickpea traits, along with their 4orresponding statistical significance represented by Q-Q plot for location 2 identified from FarmCPU model

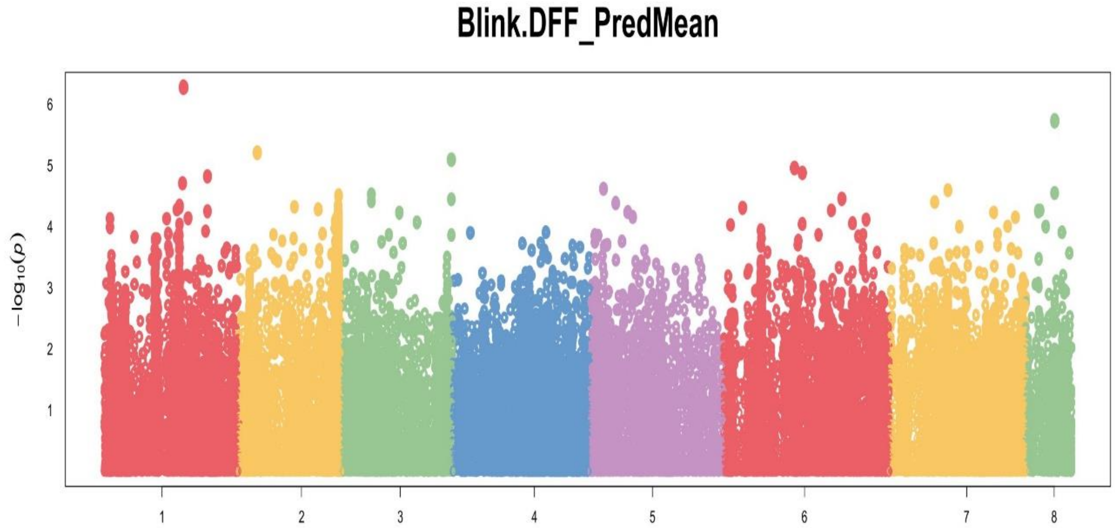

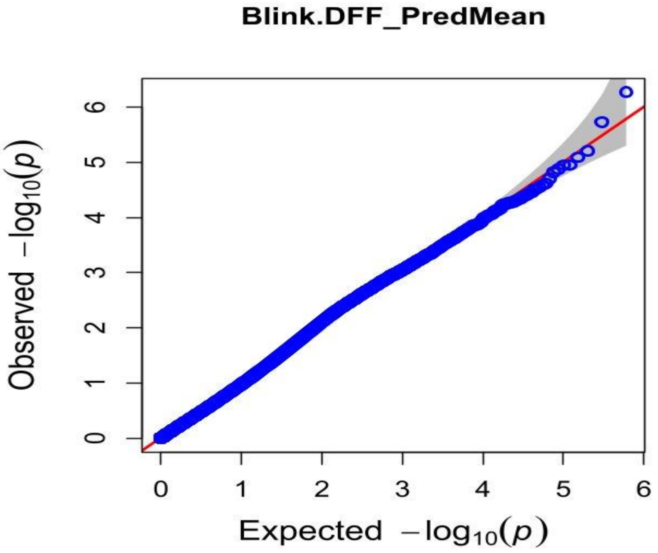

Supplementary Figure5a: Manhattan plot illustrating SNPs linked to Days fifty percentage flowering along with their corresponding statistical significance represented by Q-Q plot.

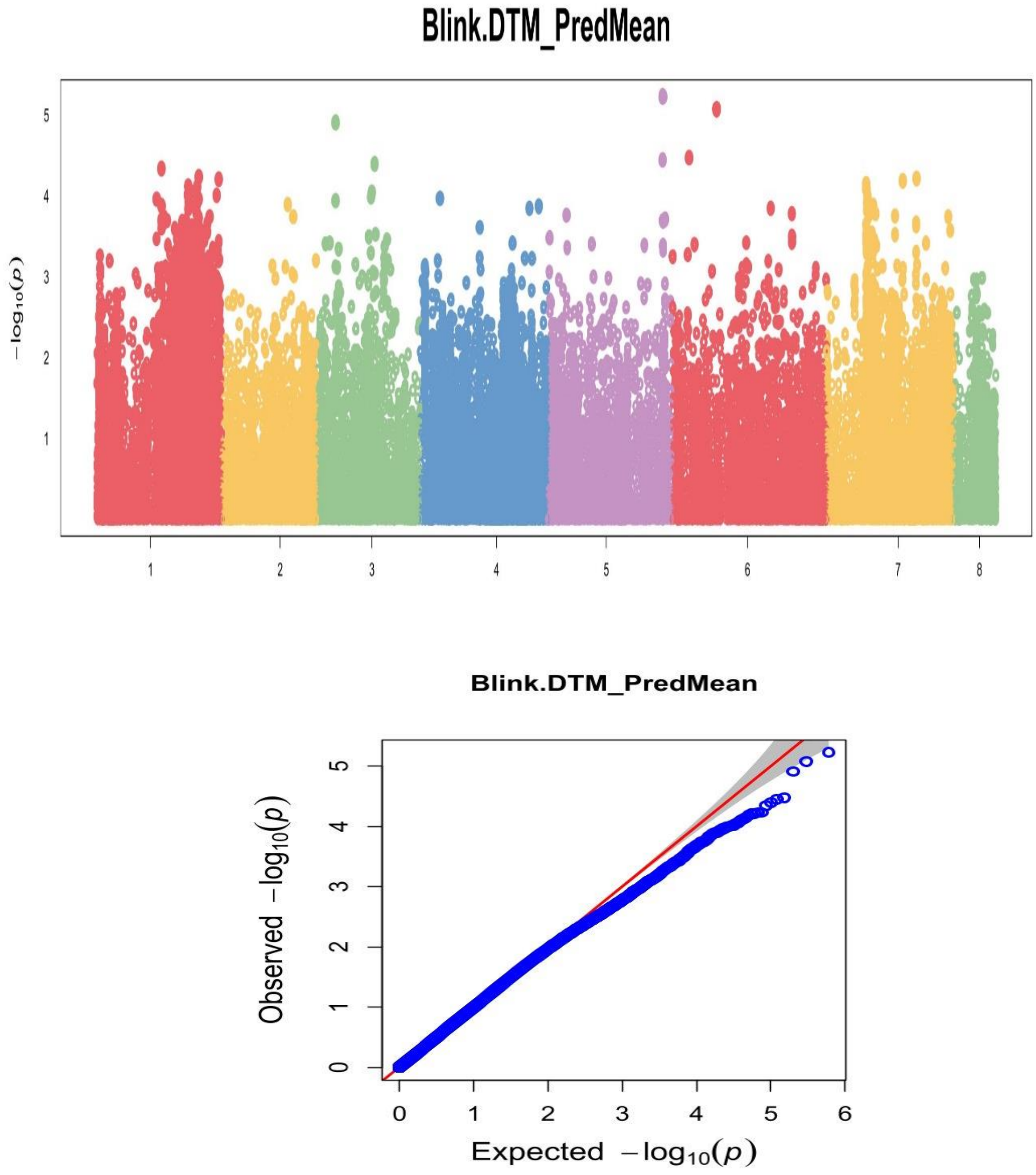

Supplementary Figure5b: Manhattan plot illustrating SNPs linked to Days to maturity along with their corresponding statistical significance represented by Q-Q plot.

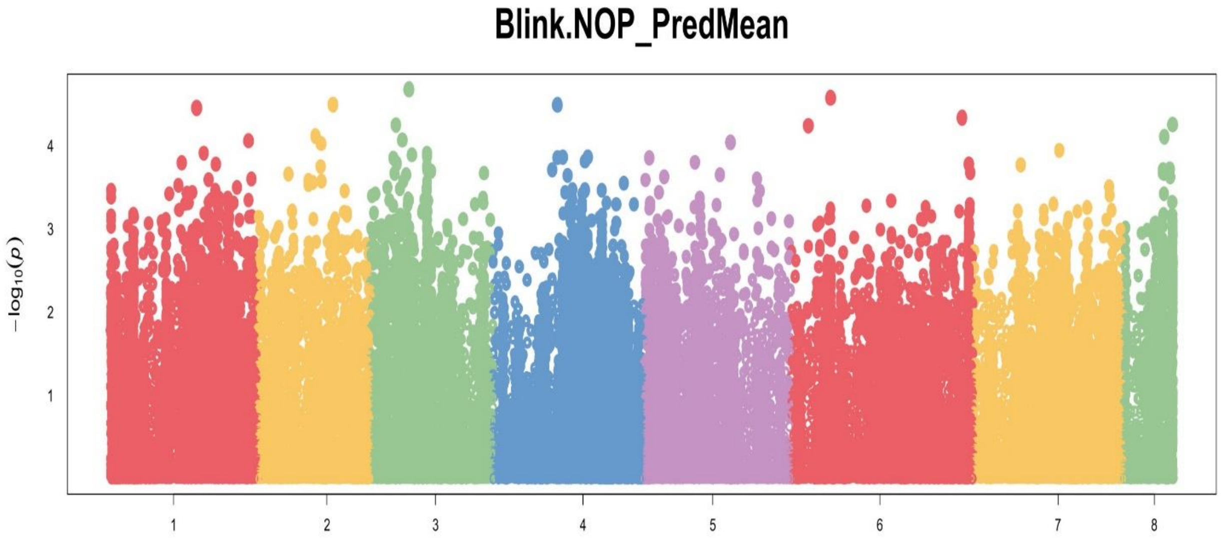

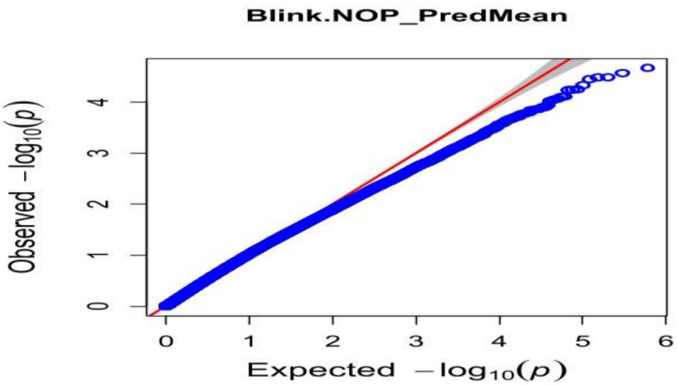

Supplementary Figure5c: Manhattan plot illustrating SNPs linked to number of pods along with their corresponding statistical significance represented by Q-Q plot.

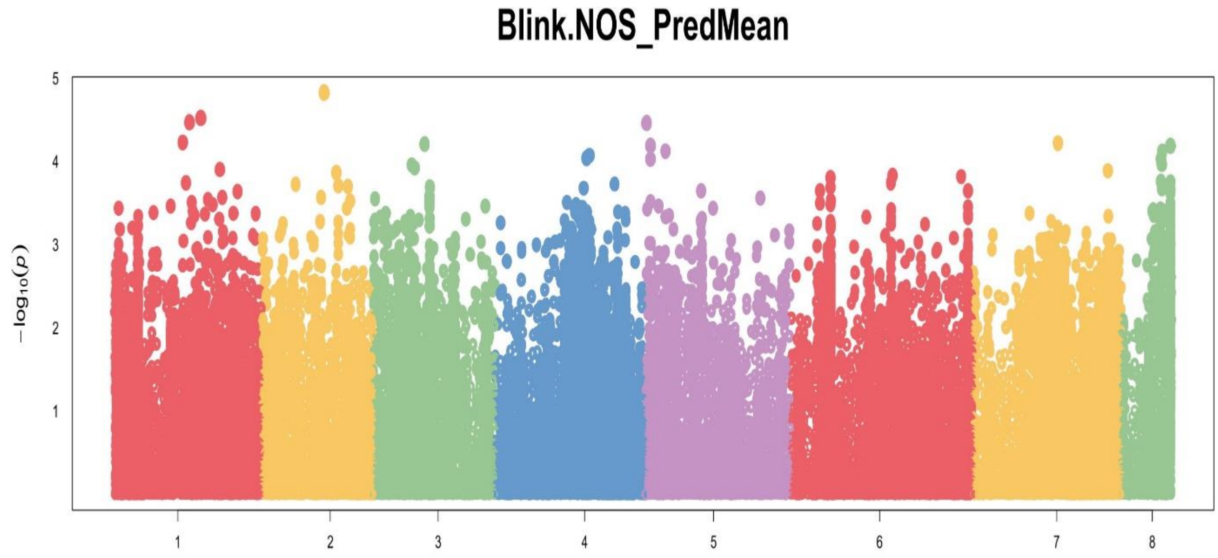

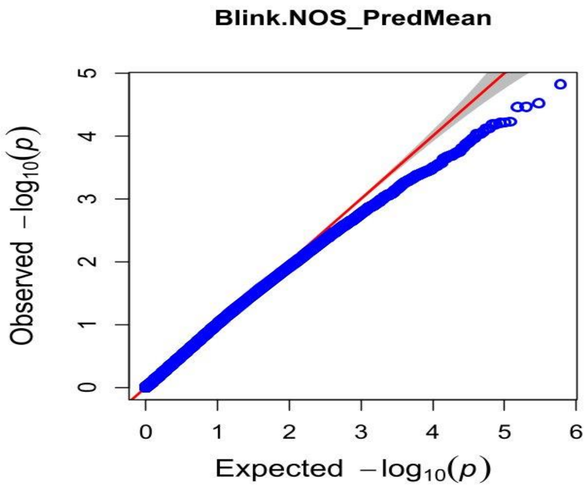

Supplementary Figure5d: Manhattan plot illustrating SNPs linked to number of seeds along with their corresponding statistical significance represented by Q-Q plot.

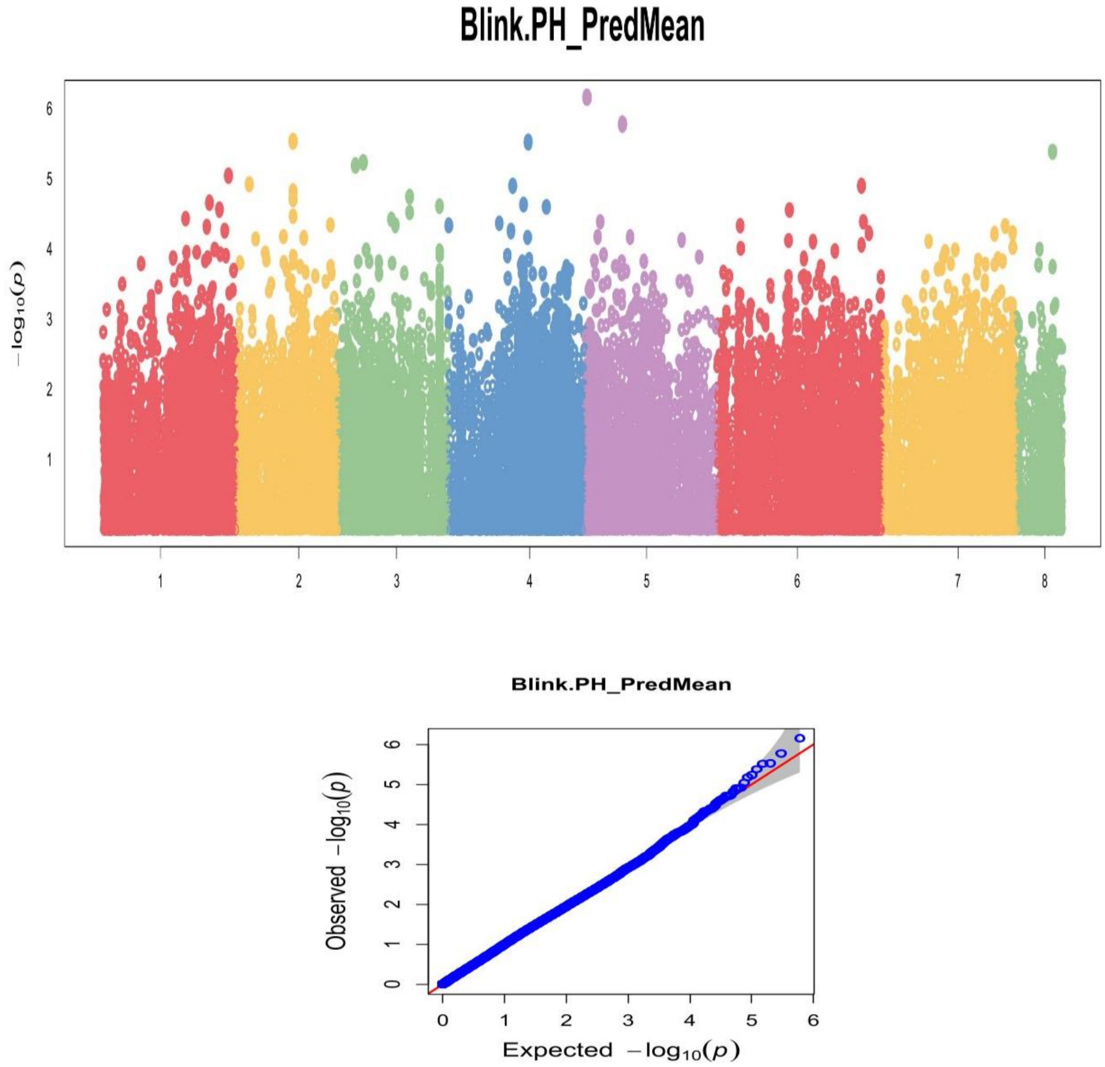

Supplementary Figure5e: Manhattan plot illustrating SNPs linked to plant height along with their corresponding statistical significance represented by Q-Q plot

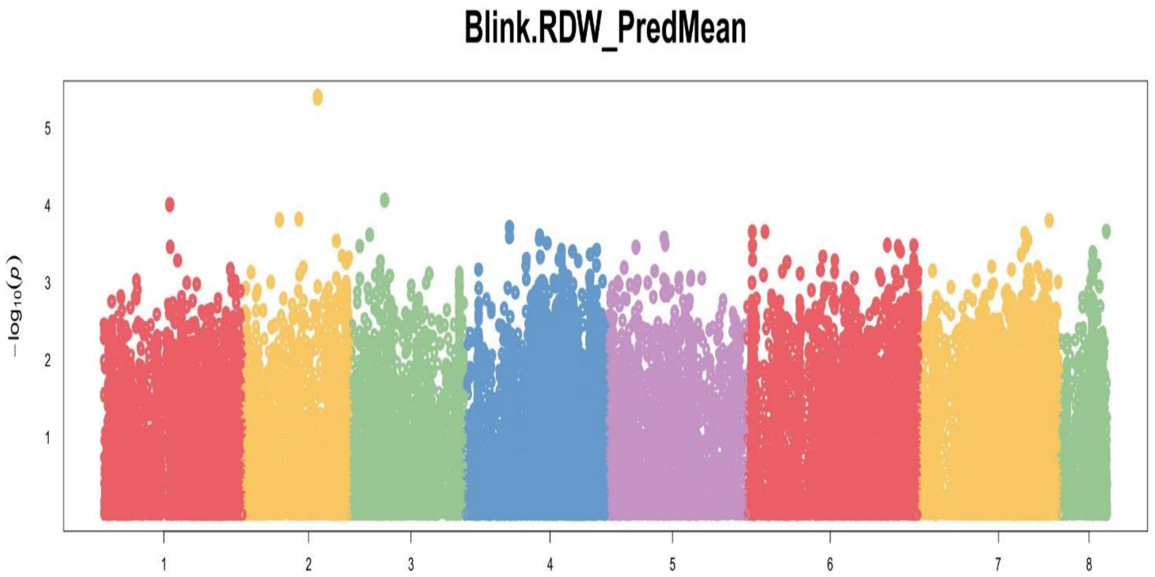

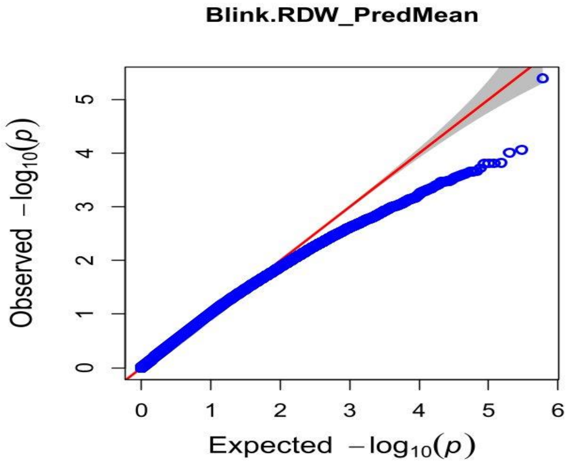

Supplementary Figure5f: Manhattan plot illustrating SNPs linked to root dry weight with their corresponding statistical significance represented by Q-Q plot

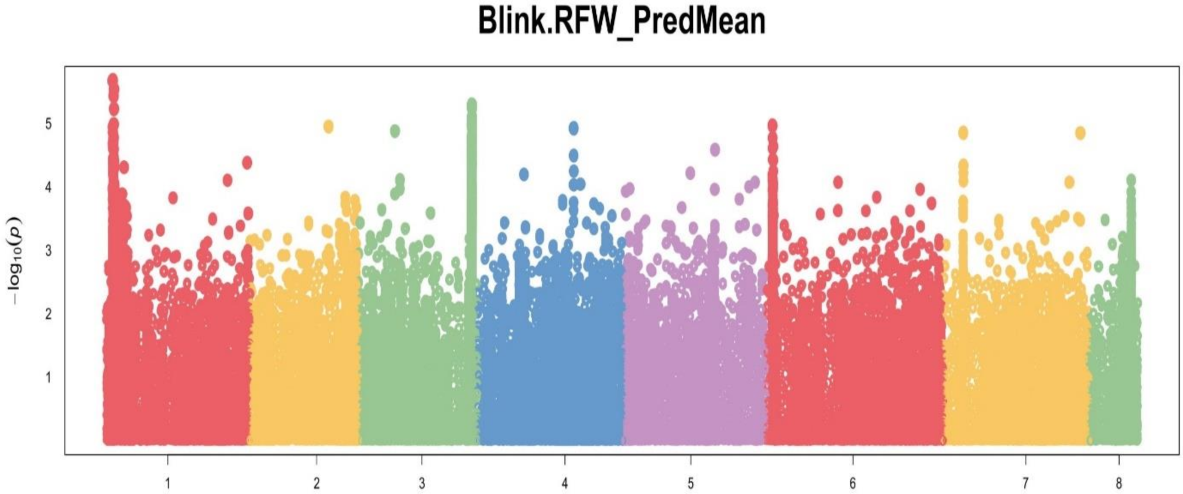

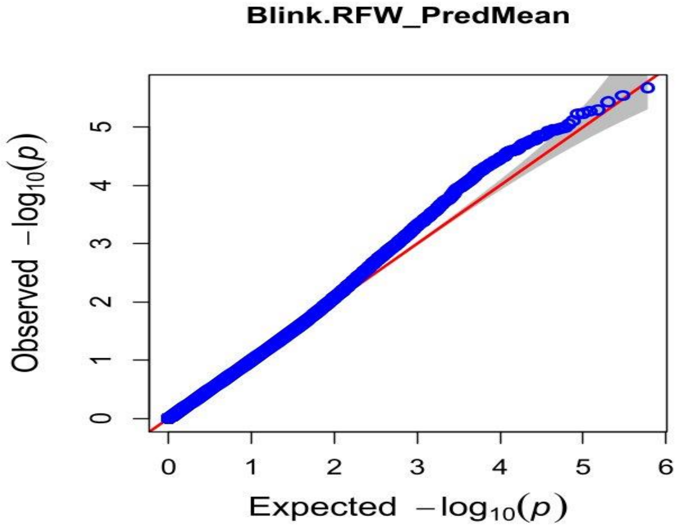

Supplementary Figure5g: Manhattan plot illustrating SNPs linked to root fresh weight with their corresponding statistical significance represented by Q-Q plot

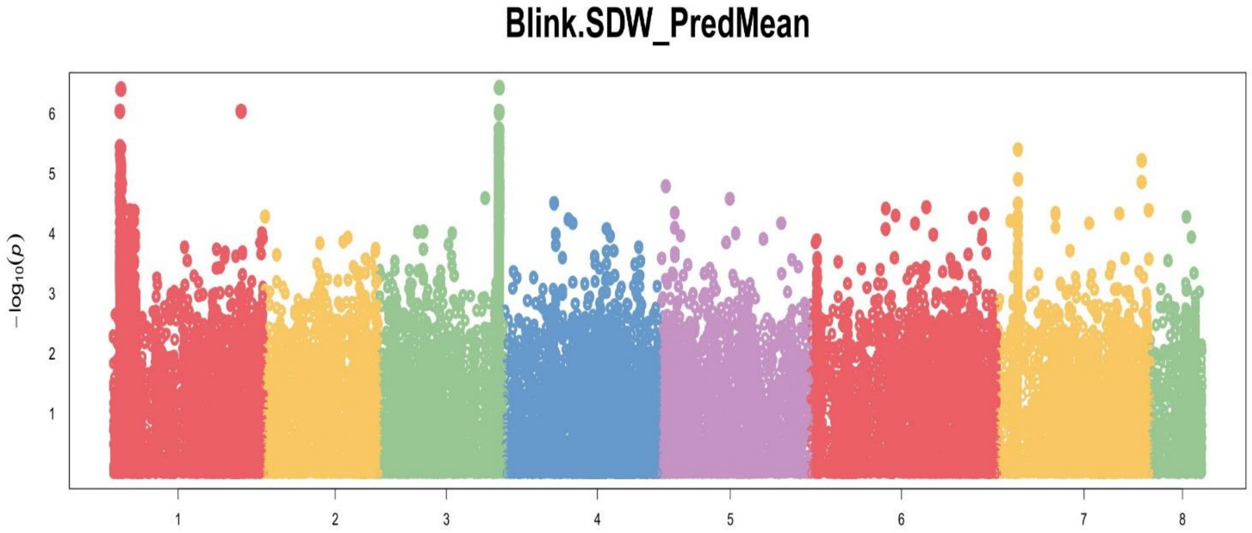

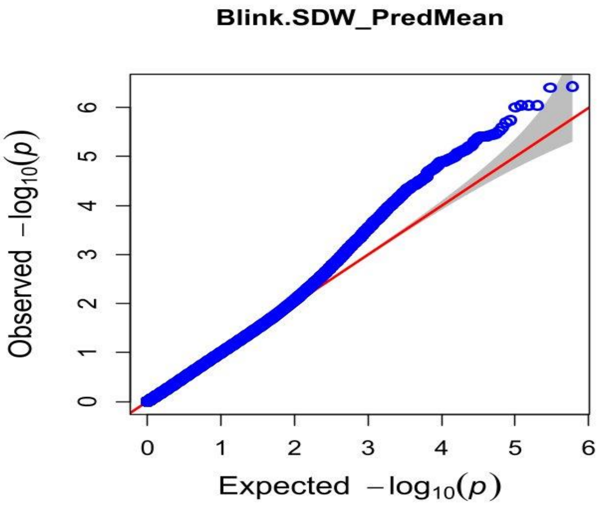

Supplementary Figure5h: Manhattan plot illustrating SNPs linked to r shoot dry weight with their corresponding statistical significance represented by Q-Q plot

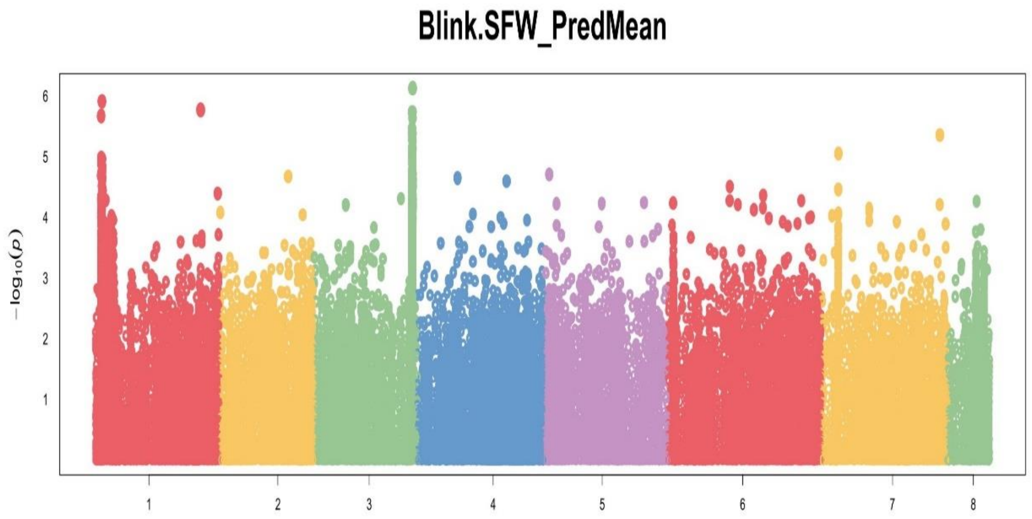

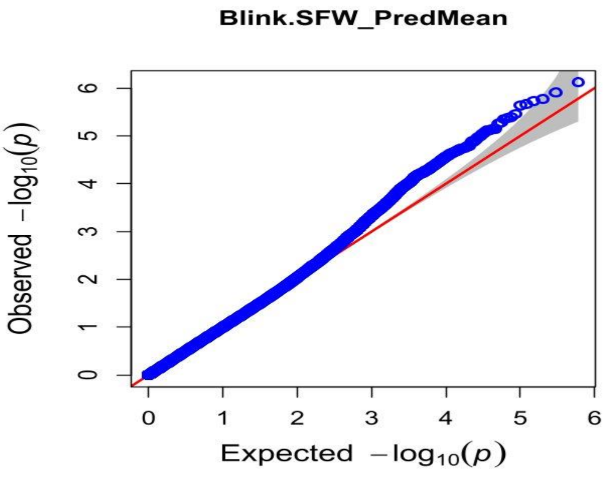

Supplementary Figure5i: Manhattan plot illustrating SNPs linked to shoot fresh weight with their corresponding statistical significance represented by Q-Q plot

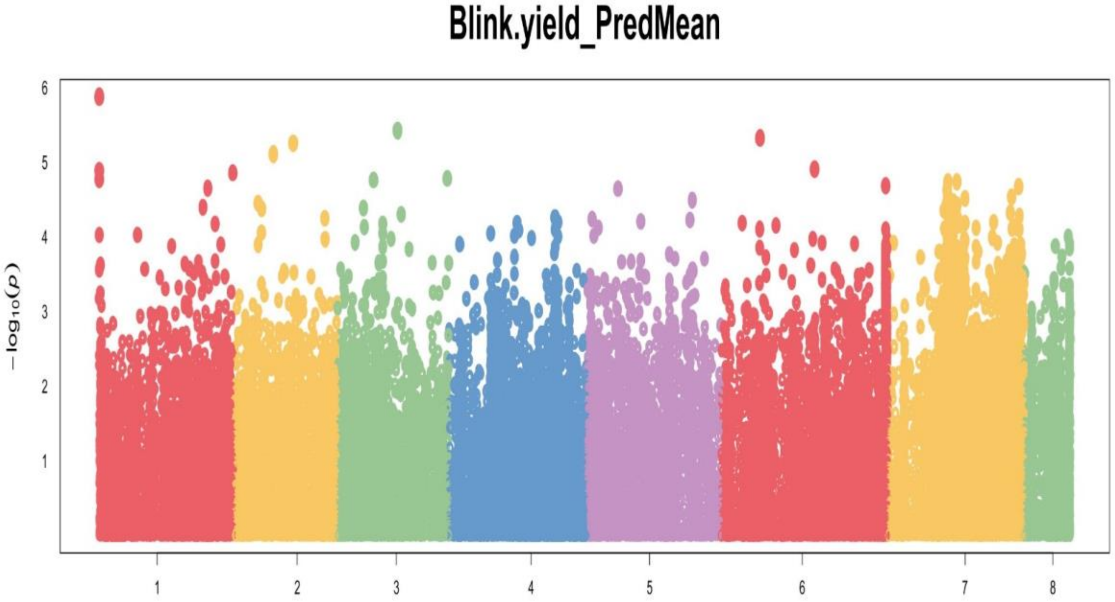

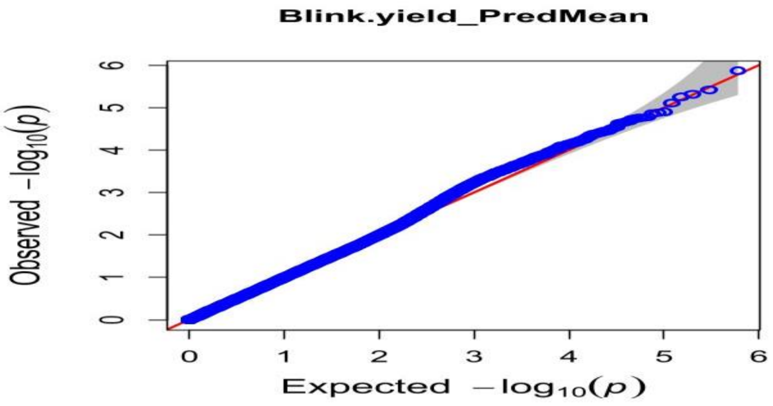

Supplementary Figure5j: Manhattan plot illustrating SNPs linked to yield with their corresponding statistical significance represented by Q-Q plot

Supplementary Figure5: Manhattan plot illustrating SNPs linked to specific chickpea traits, along with their corresponding statistical significance represented by Q-Q plot for location 2 identified from Blink model.
